# The impact of HIV infection on the frequencies, function, spatial localization and heterogeneity of T follicular regulatory cells (TFRs) within human lymph nodes

**DOI:** 10.1186/s12865-022-00508-1

**Published:** 2022-07-01

**Authors:** Bongiwe Mahlobo, Faatima Laher, Werner Smidt, Funsho Ogunshola, Trevor Khaba, Thandeka Nkosi, Anele Mbatha, Thandekile Ngubane, Krista Dong, Ismail Jajbhay, Johan Pansegrouw, Zaza M. Ndhlovu

**Affiliations:** 1 Africa Health Research Institute (AHRI), Nelson R. Mandela School of Medicine, University of KwaZulu-Natal, Durban, South Africa; 2 HIV Pathogenesis Programme, Doris Duke Medical Research Institute, Nelson R. Mandela School of Medicine, University of KwaZulu-Natal, Durban, South Africa; 3 KwaZulu-Natal Research Innovation and Sequencing Platform (KRISP), School of Laboratory Medicine and Medical Sciences, College of Health Sciences, University of KwaZulu-Natal, Durban, South Africa; 4 Ragon Institute of Massachusetts General Hospital, Massachusetts Institute of Technology, Harvard University, Cambridge, MA USA; 5 KwaZulu-Natal Department of Health, Prince Mshiyeni Memorial Hospital, Durban, South Africa

**Keywords:** Follicular regulatory T cells (TFRs), Follicular helper T (TFH) cells, Lymph node, Biomarkers, HIV

## Abstract

**Background:**

HIV eradication efforts have been unsuccessful partly due to virus persistence in immune sanctuary sites such as germinal centres within lymph node (LN) tissues. Recent evidence suggests that LNs harbour a novel subset of regulatory T cells, termed follicular regulatory T cells (TFRs), but their role in HIV pathogenesis is not fully elucidated.

**Results:**

Paired excisional LN and peripheral blood samples obtained from 20 HIV-uninfected and 31 HIV-infected treated and 7 chronic untreated, were used to determine if and how HIV infection modulate frequencies, function and spatial localization of TFRs within LN tissues. Imaging studies showed that most TFRs are localized in extra-follicular regions. Co-culture assays showed TFRs suppression of TFH help to B cells. Importantly, epigenetic and transcriptional studies identified DPP4 and FCRL3 as novel phenotypic markers that define four functionally distinct TFR subpopulations in human LNs regardless of HIV status. Imaging studies confirmed the regulatory phenotype of DPP4^+^TFRs.

**Conclusion:**

Together these studies describe TFRs dynamic changes during HIV infection and reveal previously underappreciated TFR heterogeneity within human LNs.

**Supplementary Information:**

The online version contains supplementary material available at 10.1186/s12865-022-00508-1.

## Background

The hallmark of HIV infection is progressive, multifactorial impairment of the immune system eventually leading to acquired immunodeficiency syndrome (AIDS). In most instances antiretroviral therapy (ART) results in rapid HIV suppression in peripheral blood with significant levels of normalization of immune parameters, such as decreased immune activation and restoration of CD4 counts. However, even with ART, the virus persists in secondary lymphoid tissues (LTs), particularly in immune privileged sites such as B cell follicles [1]. LTs are the principal sites of adaptive immune responses and harbour high concentrations of CD4^+^ T cells, the main target of HIV infection [2]. Thus, understanding host immune responses that underpin HIV persistence in LTs such as suboptimal immune functioning and immune regulation is needed to achieve novel cure strategies.

T follicular helper (TFH) cells in immune-privileged lymphoid tissues represent an important reservoir during chronic HIV infection. But their physiological function is to provide help to B cells within geminal centres (GCs) by selectively stimulating B cell clones with high affinity towards pathogenic antigens to promote robust humoral responses while preventing selection of self-reactive B cell clones [3]. However, dysregulated TFH function has deleterious consequences such as the development of several autoimmune diseases [4], and HIV associated hypergammaglobulinemia [5], hence the need to better understand how TFH functions are regulated.

Recent work described a novel subset of regulatory T cells (Tregs) in LNs, termed T follicular regulatory cells (TFRs). TFRs regulate TFH frequencies and helper function [6–11], though the cellular targets and molecular mechanism that underpin regulatory processes are unclear. A number of different mechanisms have been proposed, including suppression of IL-21 and IL-4 production and downregulation of ICOS expression by TFH [12]. Other studies suggests that TFRs suppression is mediated via CTLA-4 binding to CD80/CD86 [9, 10]. A more recent study suggests that TFRs suppressive function is principally modulated by B cell receptor (BCR) signalling and CD40-CD40L interactions [13]. Clearly, more work is needed to understand the mechanism of action and function of TFRs.

Progress on elucidating the role of TFRs in human health and diseases has been hampered by lack of suitable human tissue samples for research. Though first described in human tonsils, most of the data on TFRs to date have been derived from small animal models [6, 8, 11, 14, 15]. Furthermore, TFRs studies are particularly challenging because of significant phenotypic overlap between TFRs and TFH cells. Both TFRs and TFH cells express CXCR5, Bcl6 and PD-1 [8, 11, 16, 17]. TFRs also express forkhead box P3 (FOXP3) and IL2RA (CD25) which are also canonical markers for conventional regulatory T cells [6, 8]. Consequently, the precise phenotype, function and topology of TFRs within human LTs remain ill-defined. Thus, the identification of well-defined markers of TFRs is critical for progress in the field. Furthermore, the effects of HIV infection on TFH frequencies and function are well-described in both human and non-human primate (NHP) models [12, 18–20], but the effect of HIV on TFRs frequencies and function remain poorly defined.

Here, we used excisional LN and blood samples from 31 treated and 7 untreated HIV-infected individuals and 20 healthy controls to investigate the spatial distribution, function, epigenetic and transcriptional programs of TFRs. Our data show that TFRs predominately localize outside the GCs. Transcriptional analysis of sorted TFRs and TFH cells identified DPP4 and FCRL3 as novel phenotypic markers that delineates TFRs into four distinct subsets, each functionally distinguishable from other subsets and TFH and conventional Tregs. The new surface markers can be used to sort TFRs for downstream omics analysis, allowing for increased opportunities to advance the field of TFRs biology in HIV pathogenesis and other infectious diseases.

## Results

### TFRs frequencies in peripheral blood and their topological distribution within lymph node tissues

**Fig. 1:**
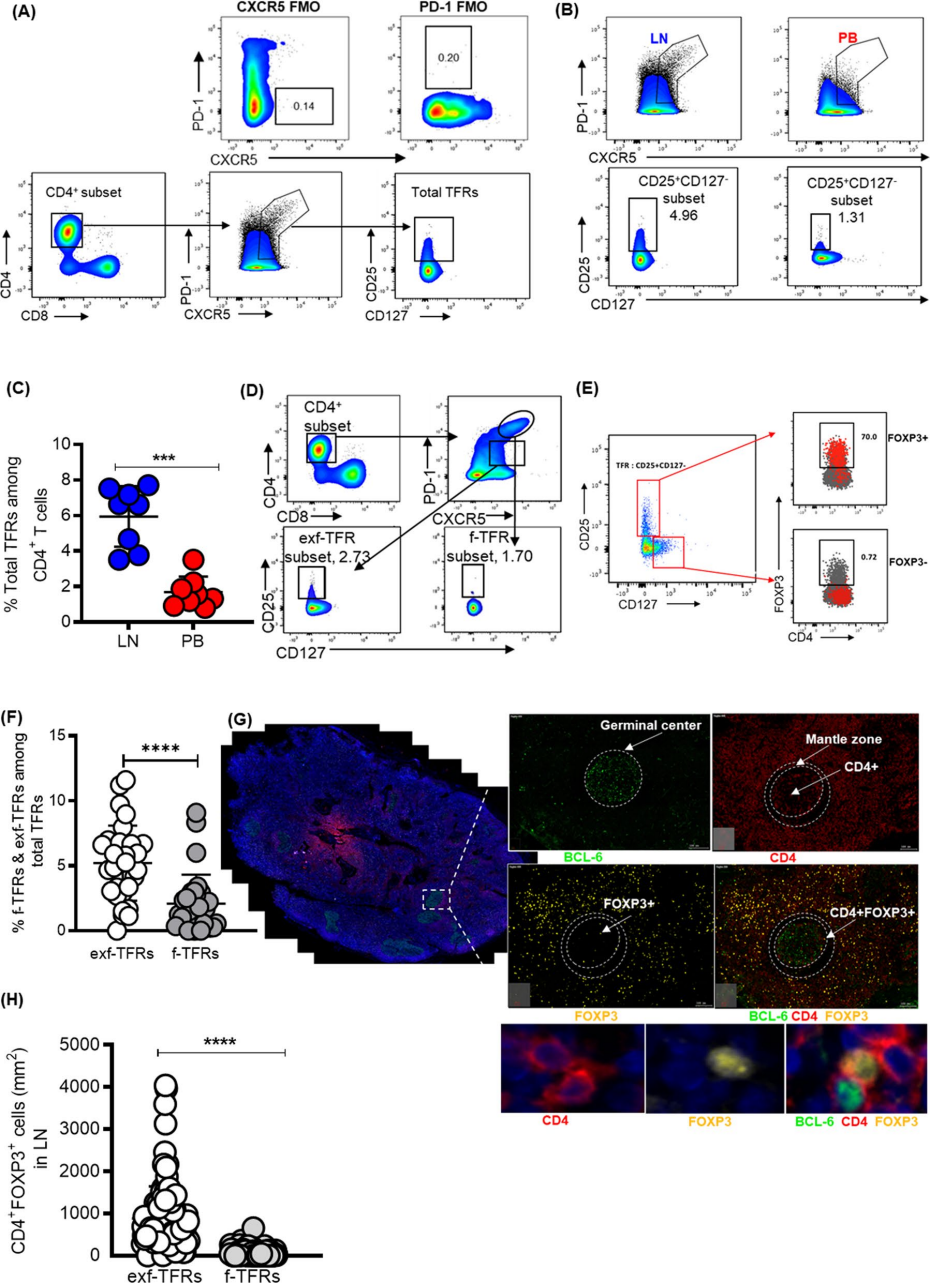
Majority of TFRs localize outside of the germinal center. **A** Gating strategy for total TFRs (CXCR5^hi/+^PD1^hi/+^CD25^+^CD127^−^) within CD4^+^ T cells in paired PB and LN samples and PD-1 and CXCR5 FMOs. **B** Representative flow cytometry plot showing total TFR frequencies in LN vs PB. **C** Summary plot showing the frequency of total TFR in LN PB samples of healthy controls **D** Representative flow cytometry plots showing proportion of f-TFR (PD-1^hi^CXCR5^hi^CD25^+^CD127^−^) and exf-TFR (PD1^+^CXCR5^+^CD25^+^CD127^−^) within CD4^+^ T cells in LNs. **E** Representative plot demonstrating overlay of CD25^+^ CD127^−^ populations and FOXP3^+^ (red) populations together with CD25^−^ CD127^+^ and FOXP3^−^ (grey) populations. **F** Summary plots comparing proportion of f-TFR and exf-TFR. **G** Representative image of immunoflouresently stained LN sections from an HIV infected subject with zoomed-in images below, LNs were stained with antibodies to BCL-6 (green), CD4 (red) and FOXP3 (yellow). Images were scanned at × 20 magnification and scale bars equal 100 μm. **H** CD4^+^FOXP3^+^ cells were quantified in the entire LN cross-section and within GCs of LNs from 6 HIV uninfected and 28 HIV-infected subjects. TissueQuest (TissueGnostics, Vienna) was used to compute CD4^+^FOXP3^+^ density in each tissue section. P values were determined using Mann–Whitney U test

We first used flow cytometry to determine normal baseline levels of total TFRs in matched LN and PBMC samples obtained from 8 healthy HIV-uninfected participants. We defined total TFRs as CD4^+^CXCR5^+/hi^PD1^+/hi^CD25^+^CD127^−^(Fig. 1A). Extremely low frequencies of TFRs were detected in peripheral blood compared to paired LN tissues (Fig. 1B, C; *p* = 0.0003). Given the paucity of peripheral TFRs, we focused the rest of the study on LN TFRs. We next assessed the microanatomical localization and define cellular neighbourhoods around TFRs within LNs. TFRs localized outside GCs, termed extra-follicular TFRs (exf-TFRs), were phenotypically defined as CXCR5^+^PD-1^+^CD25^+^CD127^−^ whereas, follicular TFRs (f-TFRs) were defined as CXCR5^hi^PD-1^hi^CD25^+^CD127^−^ (Fig. 1D). Flow cytometry analysis of LN TFRs identified significantly greater frequencies of exf-TFRs compared to f-TFRs (*p* < 0.0001; Fig. 1D, F). Results showed that CD25^+^ CD127^−^ CD4^+^ express FOXP3^+^ which is a canonical marker for regulatory CD4^+^ T cells (Fig. 1E). These results were verified by multi-colour immunofluorescence (IF) imaging, in which FOXP3 co-staining with CD4 was used to identify TFRs [12] and BCL-6, a marker for active GCs was used to demarcate follicular from non-follicular regions of the LNs. Representative images (Fig. 1G) and aggregate data (Fig. 1H) corroborated flow data showing significantly greater frequencies of exf-TFRs compared to f-TFRs (*p* = 0.0001). These data are consistent with previous reports in human mesenteric LNs showing that TFRs predominantly localise outside the GC [21].

### TFRs function and cytokine production profile

**Fig. 2:**
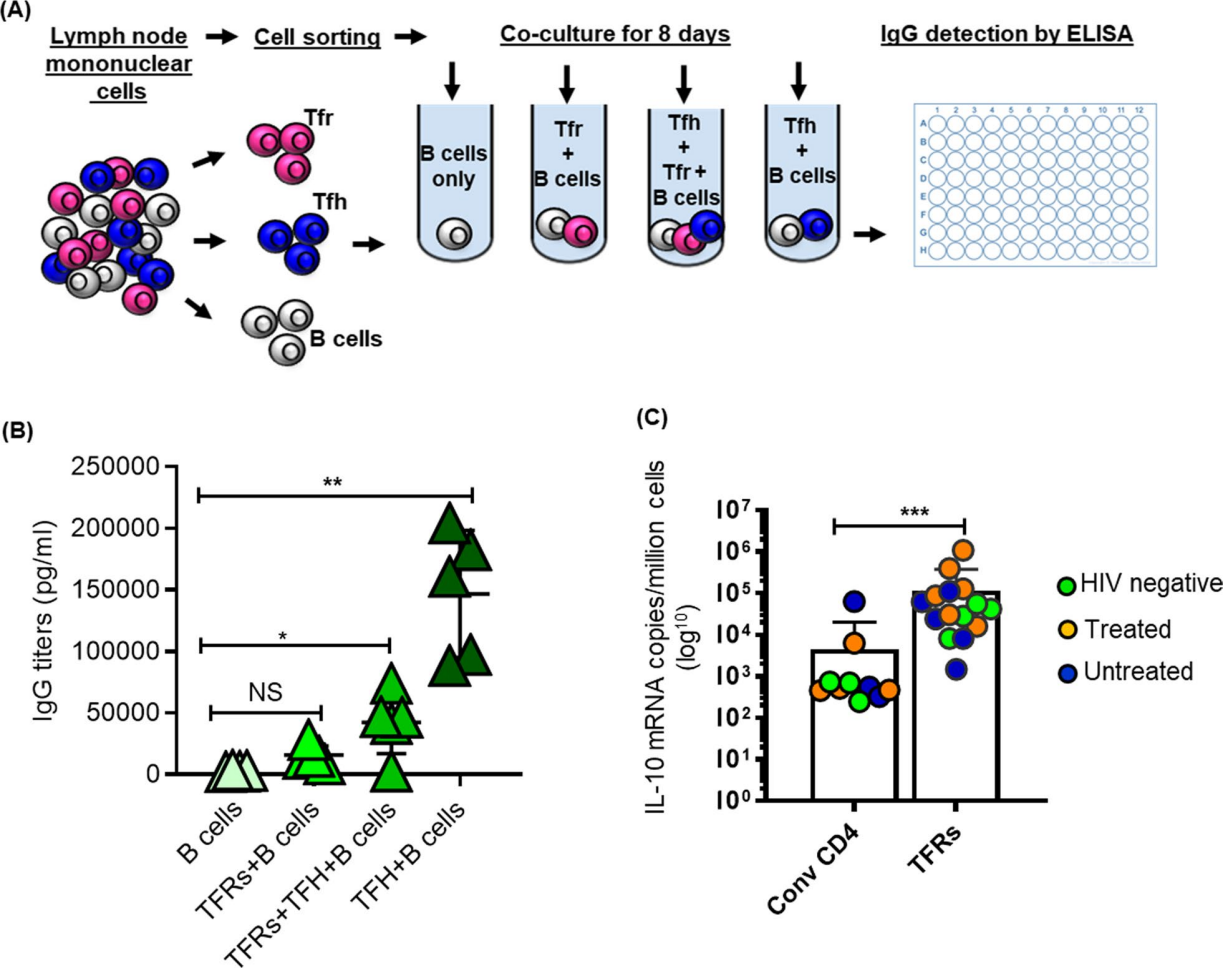
TFRs function and cytokine production profile. **A** Schematic of experimental design for co-culture assays. To increase cell numbers for the assay lymph node mononuclear cells (LMCs) were sorted for naïve B cells and enriched for TFRs (CD4^+^CD25^−^CD127^+^) and TFH cells (CD4^+^CD25^+^CD127). The TFH and naïve B cell cells were co-cultured for 8 days in presence or absence TFRs. As negative control B cells were cultured alone. IgG in the supernatant was detected by an ELISA. **B** Summary plot of IgG antibody detection by ELISA. **C** mRNA expression of IL-10 detected by ddPCR in HIV negative (n = 4), HIV-infected treated (n = 8) and HIV-infected untreated (n = 5) subjects. P values were determined using Mann–Whitney U test

TFRs have been associated with both suppressor and helper functions [9, 17, 22–25]. Having established that most TFRs reside outside GCs, we next interrogated cell-to-cell suppression of TFH helper function on B cells. TFH cells (5 × 10^4^) were co-cultured with autologous naïve B cells (5 × 10^4^) in the presence or absence of TFRs (5 × 10^4^) as illustrated in Fig. 2A. Consistent with previous reports [13], addition of TFRs into TFH-B cell co-cultures significantly decreased total IgG secretion compared to co-cultures without TFRs (Fig. 2B). These data demonstrate the capacity of TFRs to directly suppress TFH help to B cells.

We then assessed TFRs capacity to produce a suppressor cytokine IL-10 using digital droplet PCR (ddPCR). Samples from 17 individuals (4 HIV negative, 8 HIV-infected treated and 5 HIV-infected chronic untreated) (Additional file 1) were used for these studies, selected based on sample availability. TFRs were sorted based on CD4^+^CXCR5^+^PD-1^+^CD25^+^CD127^−^ phenotype. Purified conventional CD4^+^ T cells (CD4^+^CXCR5^−^PD-1^−^) were used as a control condition. B2M was used to normalize expression levels of IL-10 and determine input cell number. TFRs expressed high levels of IL-10 mRNA compared to bulk CD4^+^ T cells (*p* = 0.0002; Fig. 2C), again highlighting the potential of TFRs exert suppressive activity even at sites distal to their localization.

Antigen-specificity of TFRs is a contested concept. Some murine studies suggest that TFRs are not antigen-specific [26], while other studies have demonstrated TFRs antigen specificity in the context of immunization with myelin oligodendrocyte glycoprotein (MOG35-55) [27]. Therefore, to determine if HIV infection induces virus-specific TFRs, we next used activation-induced marker (AIM) assay which identifies antigen specificity based on the expression of activation markers following stimulation with cognate antigens [28]. LMCs from 3 healthy controls and 5 chronic HIV-infected individuals on ART were stimulated with HIV-1 peptide pools spanning entire Gag, Nef and Env proteins for 18 h and compared to unstimulated negative controls. Staphylococcal enterotoxin B (SEB) stimulation was used as a positive control. AIM^+^ TFRs were identified as OX40^+^ and programmed death ligand (PD-L1)^+^ after background subtraction based on the unstimulated control condition [28]. HIV-specific TFRs were readily detectable in three HIV-positive donors following stimulation with HIV-Gag peptide pools (*p* = 0.04; Additional file 2A, 2B). No significant responses were observed following stimulation with Env and Nef peptide pools. Healthy controls had no detectable responses (Additional file 2A, 2B). These studies suggests that antigen specificity may be a true phenomenon of TFRs in the setting of HIV infection, but more data is needed to substantiate this finding.

### Frequencies of TFRs relative to TFH cells following ART initiation

**Fig. 3:**
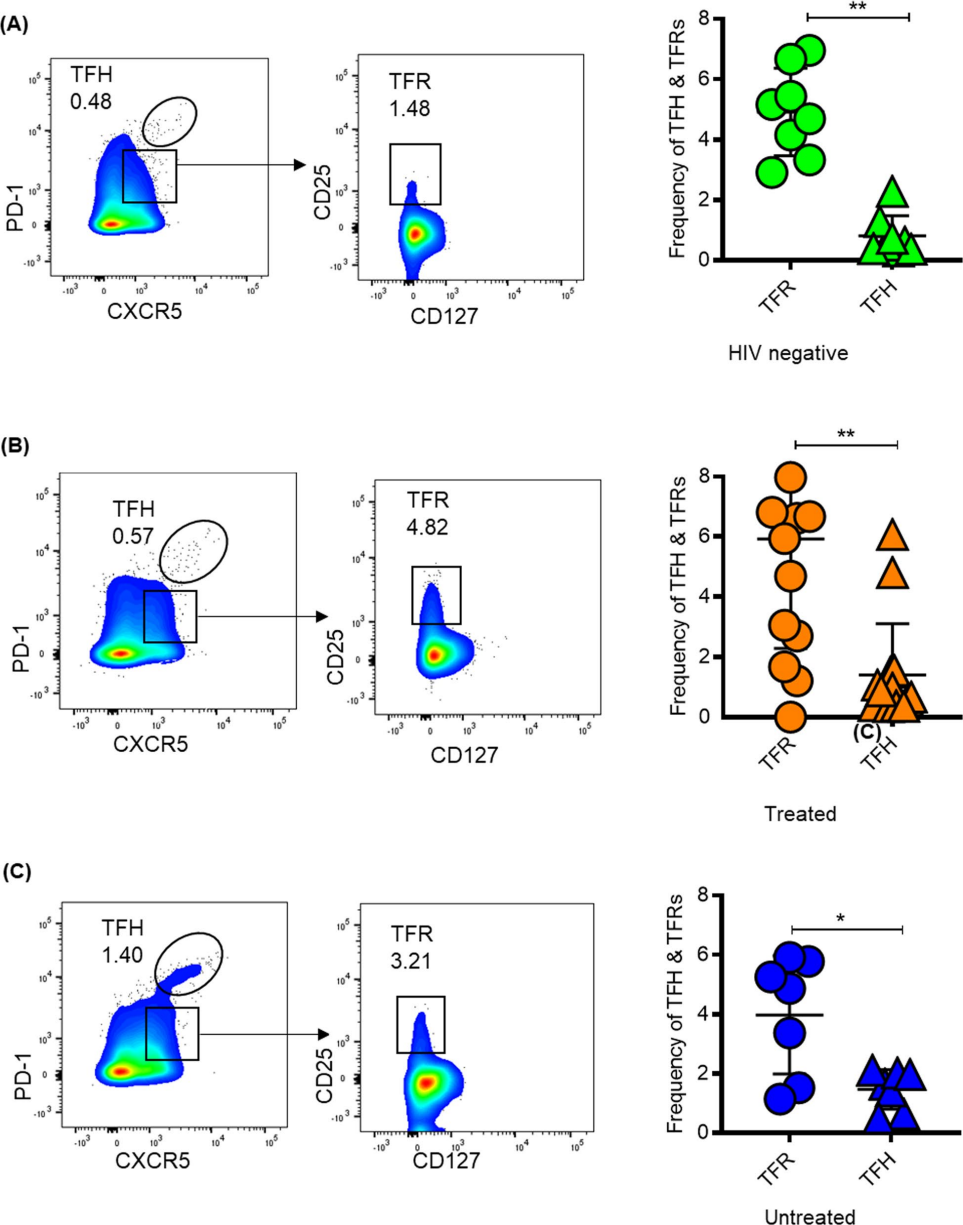
Frequencies of TFRs and TFH cells according to HIV and treatment status. Representative Flow plots and aggregate data showing frequencies of TFRs relative TFH according to disease and treatment status in (**A**) HIV negative donors, (**B**) treated and (**C**) chronic untreated. *P* values were determined using Mann–Whitney U test. *P* values were determined using Mann–Whitney U test

High frequencies of TFRs have been associated with lower TFH frequency, suggesting that the expansion of TFRs diminishes TFH frequencies and downstream B cell antibody production [12, 19, 29]. Therefore, we next investigated if HIV infection and ART initiation alters the frequencies of TFRs relative to TFH cells. Flow cytometry was used to compare TFRs and TFH cells frequencies in healthy controls (n = 8), treated (n = 15) and chronic untreated (n = 7) individuals (Additional file 3). TFH and TFRs were defined using the gating strategy shown in Addition file 4. Treatment duration varied widely ranging from 60–926 days following detection of HIV RNA. The percentage of TFRs among LN CD4^+^ T cells were greater than the proportion of TFH cells in HIV negative (*p* = 0.007, Fig. 3A), treated donors (*p* = 0.002, Fig. 3B) and chronic untreated donors (*p* = 0.01, Fig. 3C). Increased TFR/TFH ratio has been shown to down-modulate antibody responses [9, 16], thus, we speculate that increased TFR frequencies may down-modulate GC responses.

### Transcriptomic definition of TFRs and TFH cells

**Fig. 4:**
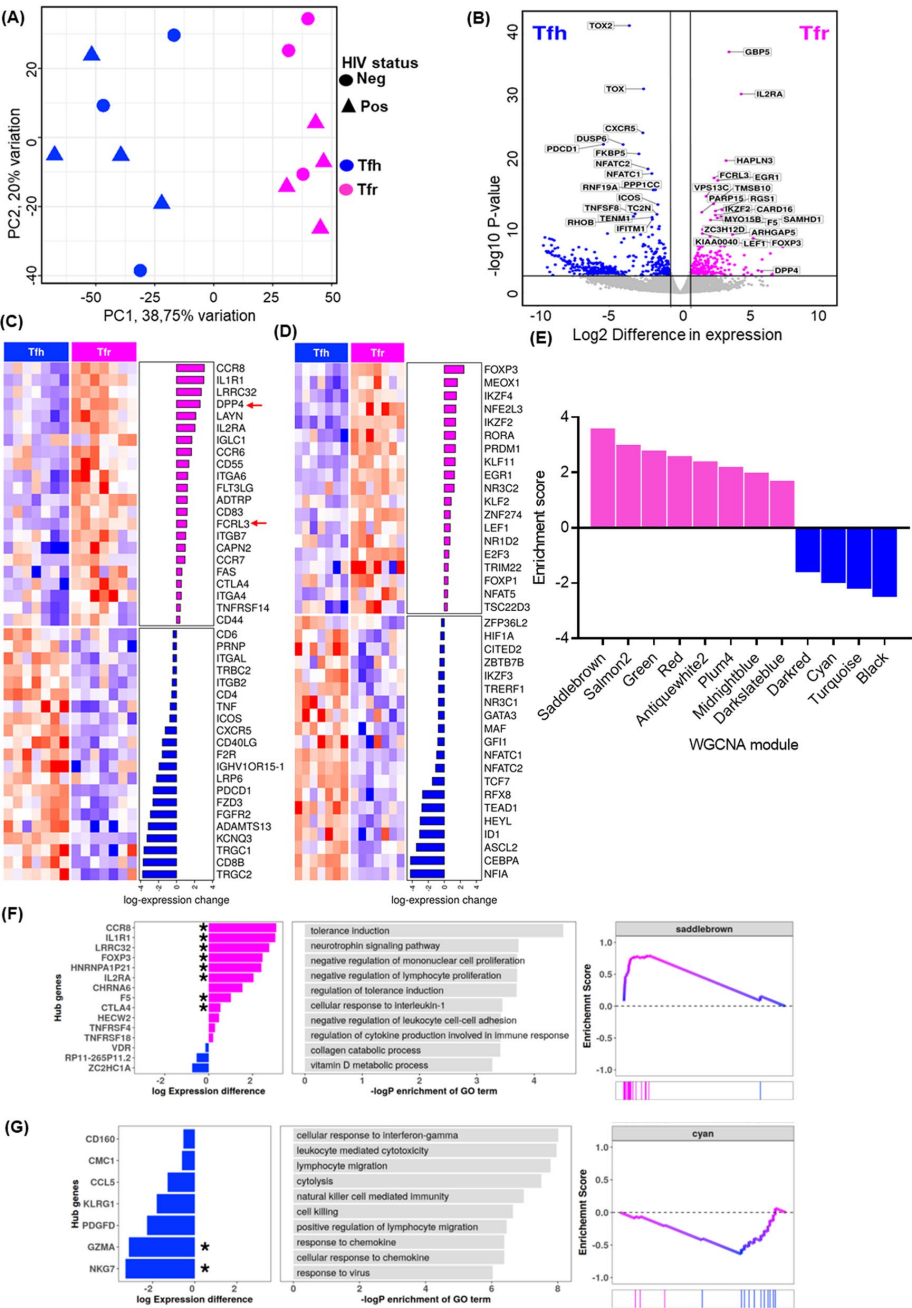
Transcriptomic definition of TFRs and TFH cells. **A** PCA plot of gene expression data of TFR and TFH cells. **B** Volcano plot depicting genes differentially expressed genes between TFRs and TFH cells, highlighting top 40 genes. Each colored dot denotes an individual gene passing our *p* value and fold difference thresholds, grey dots represent the genes below the selected threshold (0.05). Heatmap of TFR and TFH cells representing differentially expressed **C** surface molecules and **D** Transcription factors. **E** RNA-Seq WGCNA modules. The colors represent each module. WGCNA functional enrichment with hub genes for TFRs **F** and **G** TFH cells

**Fig. 5:**
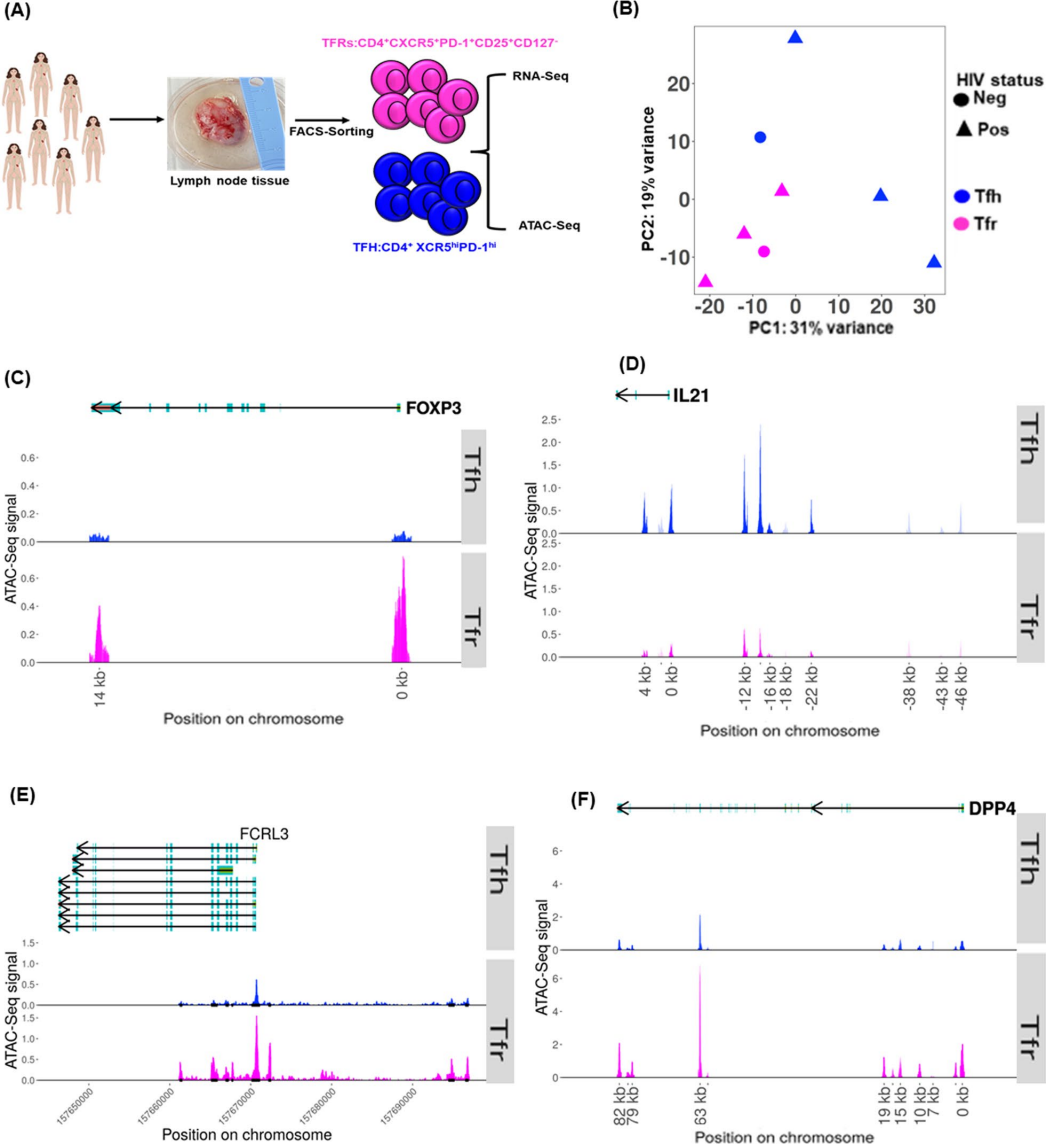
Epigenomic definition of TFRs and TFH cells. **A** Experimental design for ATAC-Seq and RNA-Seq experiments. **B** PCA plot of ATAC-Seq signal in TFRs and TFH cells. The top 10% of ATAC-Seq peaks (merged between subsets) by variance were used to create the PCA plot. Differential accessibility of canonical TFRs and TFH genes **C** FOXP3 **D** IL-21 and novel genes **E** FRL3 **F** DPP4

Given the reported overlap between TFR and TFH in terms of phenotype and functions [30–32], we next investigated their transcriptomic differences. We performed RNA-seq on 7 biological replicates of FACs-sorted TFRs and TFH cells obtained from 4 HIV-infected and 3 healthy donors (Additional file 5). RNA was extracted from sorted TFRs and TFH cells and subjected to high-throughput sequencing. Principal component analysis (PCA) segregated TFRs from TFH cells showing 38.75% of the variance in principal component 1 [PC1] (Fig. 4A). Next, we generated a list of differentially expressed (DE) genes between TFRs and TFH cells. Differential expression analysis was performed using Sleuth [33] and genes with false discovery rate (FDR) < 0.05 and 1.5-fold change were considered to be differentially expressed. We identified 904 DE genes, of which 229 genes were up-regulated and 675 down-regulated in TFRs relative to TFH cells (Additional file 6). We next generated a volcano plot highlighting the top 40 TFRs up-regulated and top 40 TFH up-regulated (TFR down-regulated) genes (Fig. 4B). GBP5 was the most DE gene between TFH and TFRs (Fig. 5B), followed by IL2RA, a signature marker of regulatory T cells, HAPLN3 and FCLR3 (Fig. 4B). The DE genes were further grouped into two major groups: surface molecules (Fig. 4C) and transcription factors (Fig. 4D). We then looked at the expression of lineage-defining markers and found both TFHs and TFRs displayed the expected expression pattern of signature markers such as high CXCR5 and IL2RA, respectively. As expected, costimulatory molecules such as CD40L and ICOS involved in B-T cell cooperation were highly expressed by TFH cells.

To identify dominant signalling pathways for each subset, we performed Weighted Gene Correlated Network Analysis (WGCNA) [34] on our RNA-Seq data set. WGCNA is a network analysis that is used to identify modules (clusters) of highly co-expressed genes. It assigns colours to each module as an identification mark. TFR genes were highly enriched in the saddlebrown module, whereas TFH genes were mostly enriched in the black module (Fig. 4E). To investigate the putative functions associated with each module, all the identified modules were subjected to functional enrichment analysis. Functional enrichment analysis of the saddlebrown module demonstrated tolerance induction, negative regulation of leukocytes and lymphocytes proliferation, negative regulation of leukocyte cell-to-cell adhesion and production of cytokines involved immune responses (Fig. 4F). Of note was the cyan module for the TFH which contained GZMH and GZMM, genes associated with cytotoxicity [35–37] (Fig. 4G). Collectively, these data provide evidence that TFRs are transcriptionally distinct from TFH cells and that their transcriptional profile is dominated by immune regulatory pathways.

### Epigenomic definition of TFRs and TFH cells

Next, we used the Assay for Transposase Accessible Chromatin with high-througput sequencing (ATAC-seq) to examine the epigenomic basis of the observed transcriptional differences between TFR and TFH subsets. ATAC-seq experiments were performed on 4 biological replicates (1 HIV-uninfected and 3 HIV-infected), all of whom had RNA-seq data as well. TFRs were sorted based on CD4^+^CXCR5^+^PD1^+^CD127^−^CD25^+^ phenotype and TFH cells were defined by CD4^+^CXCR5^hi^PD1^hi^ phenotype (Additional file 4). Experimental design is shown in Fig. 5A. We ran the ATAC-Seq data through a pipeline that includes, peak calling and differential peak analysis. We observed a clear delineation in overall genome-wide accessibility between the two cell subsets as depicted in ATAC-Seq PCA plot, showing 31% of the variance in principal component 1 [PC1] (Fig. 5B). We next performed differential peak analysis at an adjusted p-value cut-off of *p* < 0.05, each corresponding to accessible regions in the genome. As an additional quality control step, we assessed the chromatin accessibility of TFRs and TFH cells canonical genes such as FOXP3 (Fig. 5C) and IL-21 (Fig. 5D). As expected, the canonical genes were more open in the appropriate subtype, respectively. Importantly, several genes that exhibited differential expression at transcription level were also differentially accessible at DNA level (Additional file 7). Of particular interest to this study was, Fc Receptor-Like protein 3 (FCLR3) (Fig. 5E), and Dipeptidyl-peptidase 4 (DPP4) (Fig. 5F), both of which have been previously associated with immune regulation [38].

### DPP4 and FCRL3 markers discriminate TFRs from TFH cells in healthy and HIV-infected human lymph node tissues

**Fig. 6:**
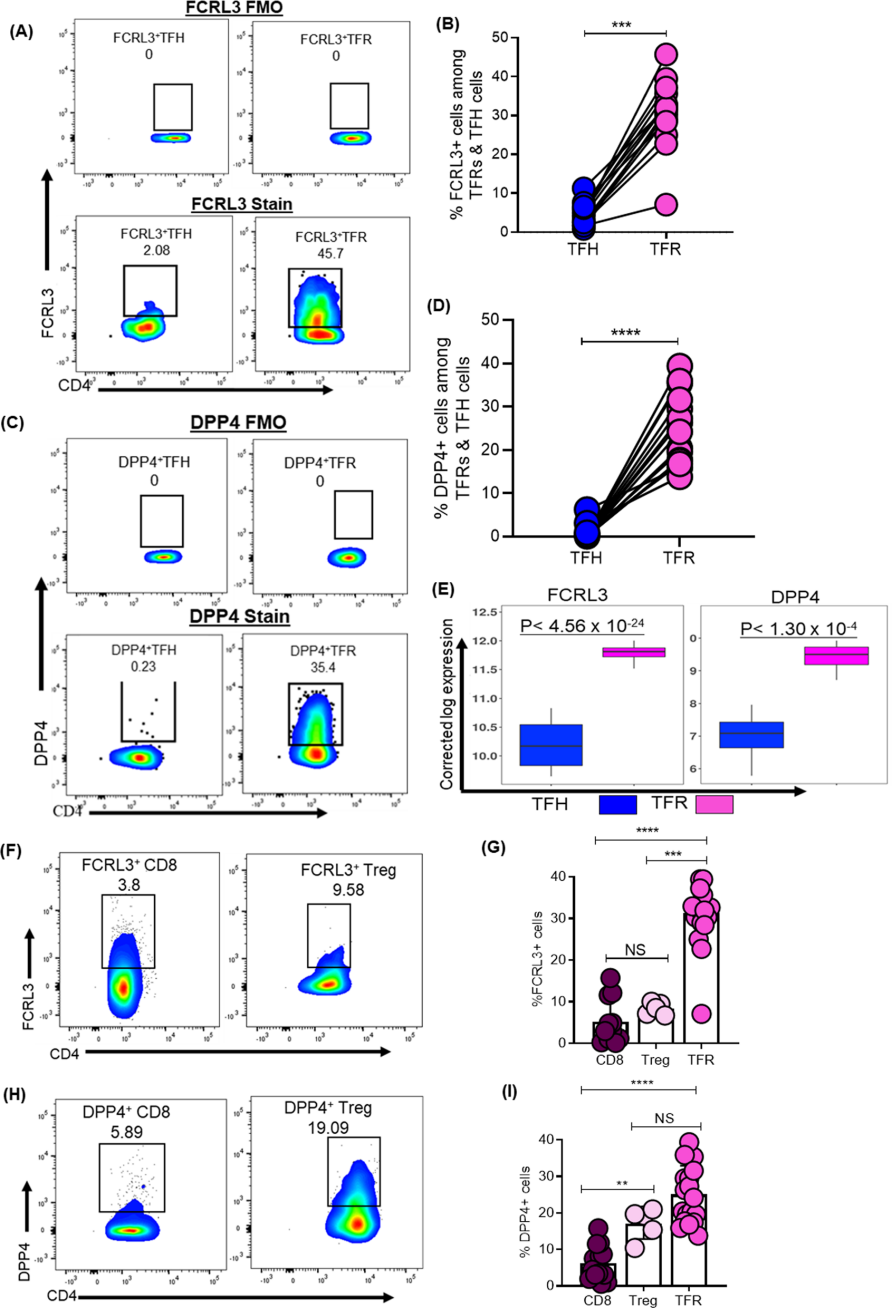
DPP4 and FCRL3 markers discriminate TFR’s from TFH cells. **A** Representative flow cytometry plots and **B** summary plot demonstrating expression of FCRL3^+^ cells among TFRs and TFHs. **C** Representative flow cytometry plots and **D** summary plot demonstrating the percentage of DPP4^+^ cells among TFRs and TFHs. **E** Boxplots with p-values of both FCRL3 and DPP4 expression. **F** Representative flow cytometry plots and **G** summary plot demonstrating expression of FCRL3^+^cells among Tregs and CD8 T cells. **H** Representative flow cytometry plots and **I** summary plot demonstrating expression of DPP4^+^ cells among Tregs, CD8 T cells and TFRs. *P* values were determined using Mann–Whitney U test

From the RNA-seq and ATAC-seq data, we selected two surface molecules namely, Fc Receptor-Like protein 3 (FCLR3) and Dipeptidyl-peptidase 4 (DPP4) for further investigation because of their reported roles in immune regulation [38]. We investigated whether these two surface proteins could be used to more definitively distinguish TFRs from TFH in human LNs of healthy and HIV-infected donors. Representative flow cytometry plots (Fig. 6A) and aggregate data (Fig. 6B) show that FCLR3 is highly expressed on TFRs compared to TFH (*p* < 0.0001). Similarly, DPP4 was also highly expressed on TFRs relative to TFH (Fig. 6C, D; *p* < 0.0001). These data corroborate RNA-Seq data (Fig. 6E). To evaluate the specificity of DPP4 and FCRL3 to TFRs, we investigated the expression of both markers by Tregs (CD4^+^CD25^+/hi^FOXP3^+^) and CD8^+^ T cells. CD8^+^ T cells were included to increase the rigor of the analysis. Representative flow cytometric plots (Fig. 6F) and aggregate data **(**Fig. 6G**)** show that FCRL3 expression on TFRs was significantly higher compared to Tregs (*p* = 0.0008) and CD8^+^ T cells (*p* < 0.0001), whereas DPP4 was expressed at intermediate levels relative to Tregs (*p* = 0.07) but higher compared to CD8^+^ T cells (*p* < 0.0001) **(**Fig. 6H, I**)**.

### FCRL3 and DPP4 define four functionally distinct TFR populations

**Fig. 7:**
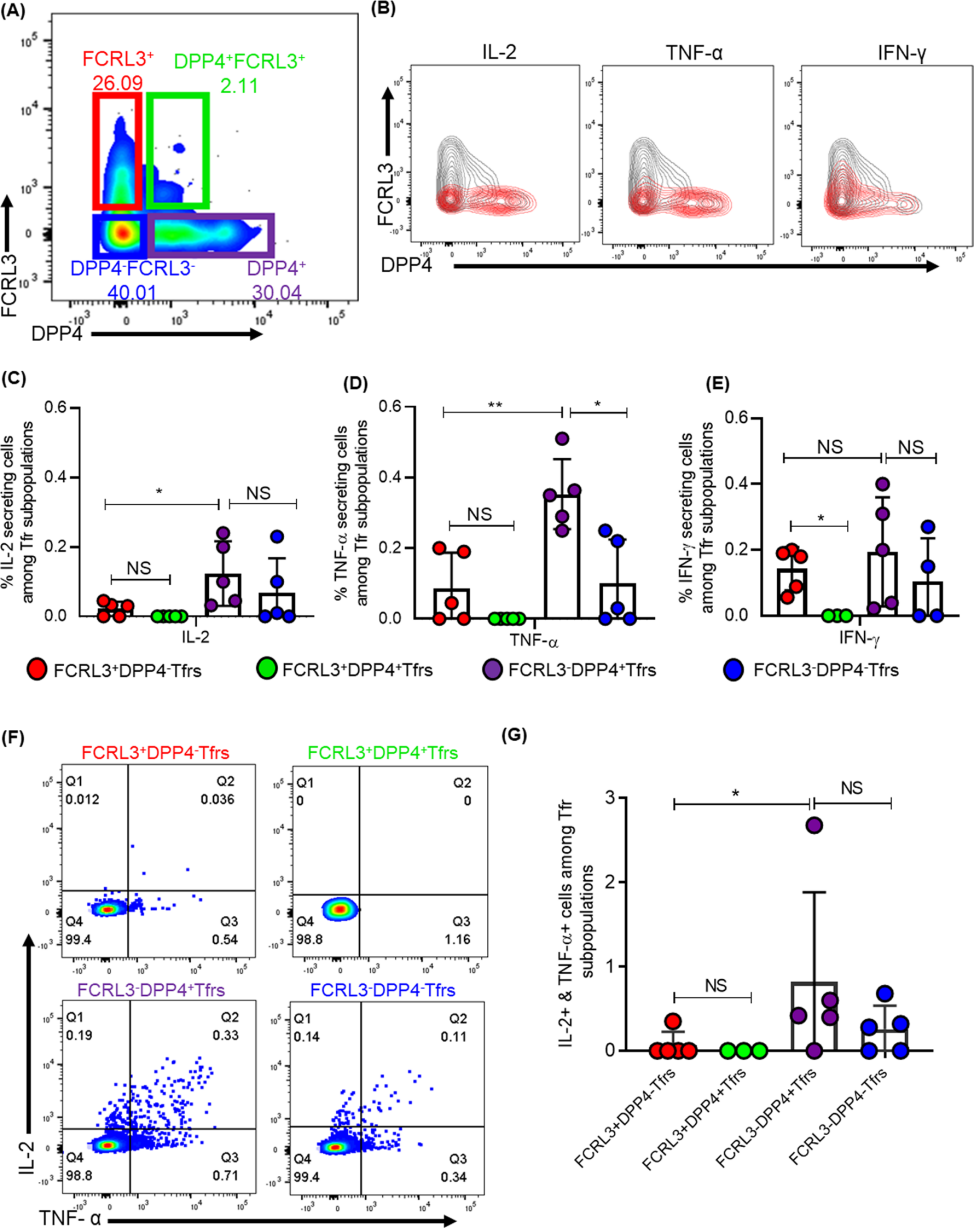

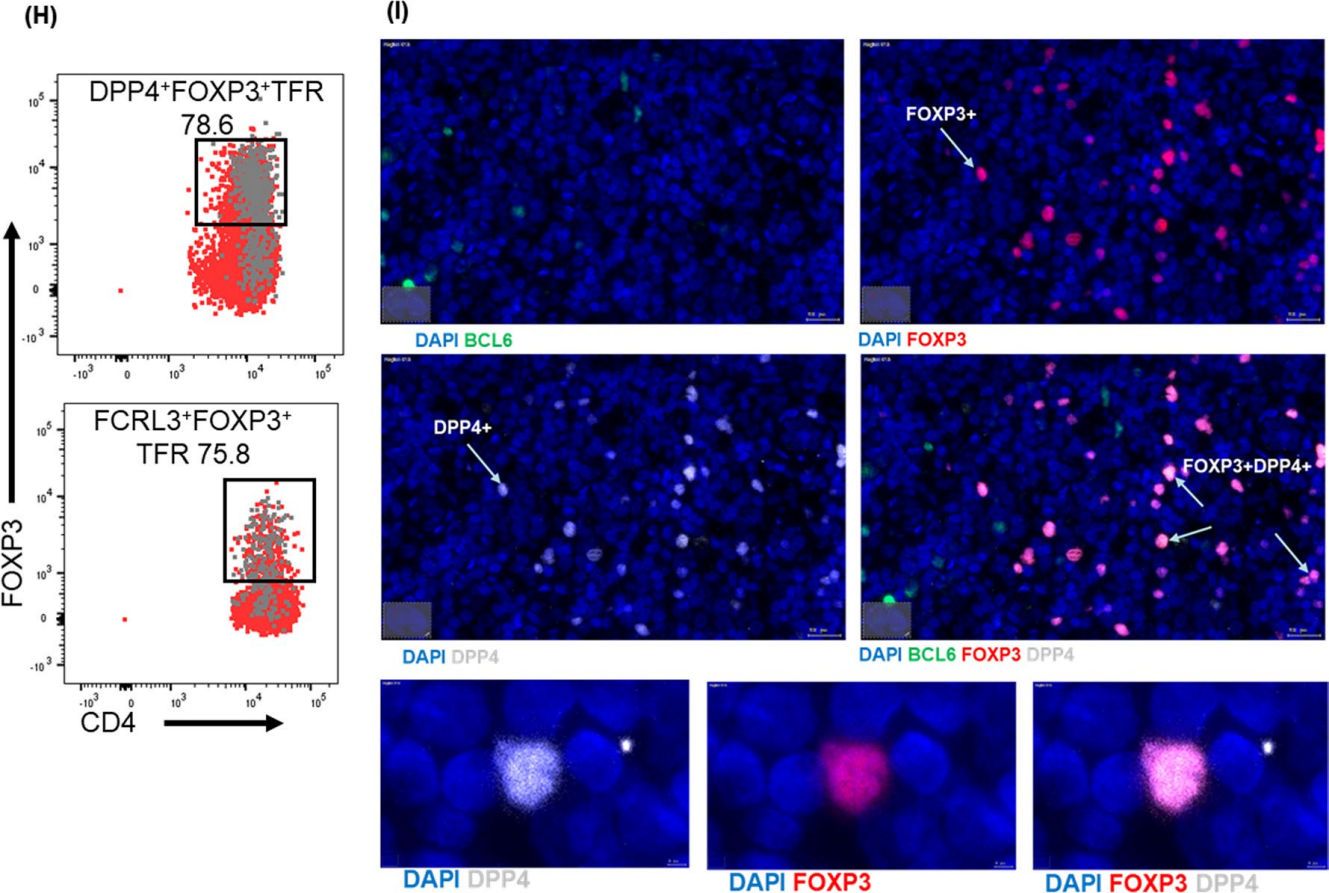
FCRL3 and DPP4 define distinct TFR populations. **A** Flow cytometry plot showing co-expression of FCRL3 and DPP4 by TFRs. **B** Representative contour plots demonstrating overlay of TFR populations (grey) and cytokine secreting cells (red) and summary plots showing **C** IL-2, **D** TNF-α and **E** IFN-γ expressing cells within TFR subsets. **F** Representative flow cytometry plots and **G** aggregate data depicting co-expression of IL-2 and TNF-α by the TFR subpopulations. **H** Representative flow plot demonstrating overlay of FCRL3^+^ TFRs (grey) populations and FOXP3^+^(red) cells together with DPP4^+^Tfrs (grey) and FOXP3^+^ cells (red). **I** Representative image of immunoflouresently stained LN sections with zoomed-in images below, LNs were stained with BCL-6 (green), FOXP3 (red) and DPP4 (white)

We next investigated whether expression pattern of DPP4 and FCRL3 define distinct TFR subsets. Co-staining of DPP4 with FCRL3 following gating on TFRs revealed four distinct subpopulations: FCRL3^+^DPP4^−^, FCRL3^−^DPP4^+^, FCRL3^+^DPP4^+^ and FCRL3^−^DPP4^−^ TFRs (Fig. 7A**)**, consistent with previous work showing TFRs heterogeneity [21, 39]. Next, we investigated if the identified subsets were phenotypically and functionally different by first assessing expression of memory markers CD62L and CD27. We found that all four TFR populations displayed CD27^hi^ and CD62L^+^ phenotype, consistent with memory cells [40, 41](Additional file 8A–C). Next, we investigated if the populations are functionally distinct by assessing cytokine secretion following stimulation with a superantigen SEB. Representative flow plots (Fig. 7B**)** and aggregate data (Fig. 7C–E), show that DPP4^+^FCRL3^−^ TFRs secreted greater amounts of IL-2 and TNF-α and a trend towards more IFN-γ production compared to FCRL3^+^DPP4^−^TFRs. FCRL3^−^DPP4^−^TFRs secreted comparable amounts of IL-2 and IFN-γ and lower TNF-α compared to DPP4^+^FCRL3^−^ TFRs. The DPP4^+^FCRL3^+^ TFR subset did not secrete any of the cytokines investigated, however, it is important to note that this population represented a very small subset. Further analyses showed that the two subsets DPP4^+^FCRL3^−^ and FCRL3^−^DPP4^−^TFRs co-produced greater amounts of IL-2 and TNF-α compared to FCRL3^+^ DPP4^−^TFRs (Fig. 7F, G). Together, these data show that DPP4 and FCRL3 maybe used to delineate four functionally distinct TFRs subpopulations.

To validate these TFR subsets as bonafide Tregs, we measured FOXP3 expression and found that 78.6% of DPP4^+^FCLR3^−^TFRs and 75.8% of DPP4^−^FCLR3^+^TFRs (Fig. 7H) were FOXP3 positive, respectively. Multi-colour immunofluorescence imaging corroborated with the flow cytometry results (Fig. 7I). Next, we assessed whether HIV-infection modulates the frequencies of the subsets. We first, compared the transcriptional profiles of TFRs between 4 HIV-infected and 3 HIV-uninfected samples. We identified a total of 72 borderline DE genes; 39 down-regulated and 33 up-regulated, but DPP4 and FCRL3 were not among them (Additional file 9A). In fact, all the seemingly DE genes were below the q-value threshold of 0.05. To further explore potential impact of HIV infection on these markers, we next used flow cytometry to compare the frequencies of the TFR subsets from 12 HIV-infected donors and 9 healthy controls. We found no significant difference in frequencies of DPP4^+^FCRL3^−^TFRs (Additional file 9B; *p* = 0.66), FCRL3^+^DPP4^−^TFRs (Additional file 9C; *p* = 0.59), DPP4^−^FCRL3^−^TFRs (Additional file 9D; *p* = 0.26) and DPP4^+^FCRL3^+^TFRs (Additional file 9E; *p* = 0.3) between HIV infected and uninfected donors. Next, we assessed if there was a relationship between the frequency of the TFR subsets and viral loads (LN, PBMC and plasma) and CD4 counts, which are markers of HIV disease progression. Again, we found no correlation between the frequencies of DPP4^+^FCRL3^−^TFRs (Additional file 9F; *p* = 0.64, r = 0.18), FCRL3^+^DPP4^−^TFRs (Additional file 9G, *p* = 0.47, r = − 0.3) and absolute CD4 counts. Similarly, both subsets did not correlate with LN and plasma or with PBMC viral loads (Additional file 9H-K**)**. Furthermore, FCRL3^+^DPP4^−^TFR and DPP4^−^FCRL3^−^TFR subsets did not correlated with absolute CD4 counts and the viral loads (data not shown). Taken together, these studies suggest that DPP4 and FCRL3 are not significantly modulated by HIV infection. However, it is important to note that we used HIV samples with very low viremia and the sample size was very small, therefore we cannot definitively rule out the possibility of uncontrolled HIV-1 infection having an effect on the two markers.

## Discussion

TFRs were recently discovered in human tonsils [6, 8, 11], but many questions related to their role in the context of HIV-1 immunopathogenesis remain unanswered. Animal studies describe TFRs as a specialized Treg subset that suppresses TFH and B cells during GC responses [6, 8, 11]. However, the role of TFRs in human disease is ill defined. Here we investigated frequencies, function, transcriptome and topological distribution of TFRs in human LNs in treated and untreated HIV infection, as well as in healthy controls. We found that TFRs predominantly localize in the extra-follicular region where TFR-mediated suppression is likely most efficient. Importantly, we identified two novel phenotypic surface markers that reliably identify TFRs, as well as group them into four subsets revealing previously underappreciated TFR heterogeneity. These studies provide new insight into the localization, function and transcriptional profile of TFRs during HIV-1 infected and uninfected individuals.

The precise functional role of TFRs in human tissues is not well understood. Murine studies have reported both suppresser and helper functions [15, 17, 22, 24, 25, 31, 32, 42–44]. In this study, direct assessment of suppressor function using three-way co-cultures demonstrated the ability of TFRs to suppress TFH-mediated antibody production by naïve B cells. RNA-Seq data show that TFRs express helios (IKZF2), Eos (IKZF4), PRDM1, CTLA-4, Lef1 and CCR8 markers that are typically associated with immune regulation [45–50]. Furthermore, RNA-Seq data showed that TFRs were highly enriched in molecular pathways related to tolerance induction, negative regulation of T cell proliferation, leukocyte cell-to-cell adhesion and regulation of cytokines involved in immune response. Together, these data are consistent with previous evidence suggesting that the dominant role of TFRs is to suppress excessive immunological responses [9, 12, 22, 39].

Contrary to the widely accepted model wherein TFRs localise within the GC [6, 8], topological evaluation of LN tissues revealed that they predominantly reside in extrafollicular regions of the LN. These data are consistent with the paradigm proposed by Sayin et al*.* that TFRs act as gate keepers regulating humoral response from “outside in” [21]. It has also been suggested that TFRs localize at T-B cell border, an important site for TFH differentiation, to moderate TFH and B cell interactions; [51, 52], and prevent activated TFH cells from entering pre-existing GC reactions [53]. Additionally, animal studies have shown direct TFR-B cell suppression, leading to reduced B cell memory differentiation and moderation of GC B cells and long-lived plasma cells [22, 54]. More work is needed to better understand and validate these potential roles of TFRs in humans. However, scarcity of suitable human LT samples and lack of TFR-specific surface markers for such studies remains a significant challenge.

Although the bulk of TFRs reside in extrafollicular regions, a small proportion are localized in GCs. Whether the follicular and extra-follicular TFRs are biologically distinct from each other remains an open question. Like closely related TFH, TFRs may consist of subpopulations of different functions ranging from immunosuppression [8, 11, 12, 17, 20, 22, 39] to promoting B cell responses in GCs [25, 31, 32, 43, 55]. Consistent with this idea, two studies recently showed that both humans and mice TFRs are heterogenous and can be subdivided into different cell subsets according to differential expression of CD25 (CD25^hi^ and CD25^−^ TFRs) [39] and PD-1 (PD-1^+^ and PD-1^−^ TFRs) [21]. Other studies suggest that Tregs, the parent population of TFRs can be subdivided into different subsets based on the expression of CTLA-4, PD-1, CD45RA, CD62L, and CD278 (ICOS) [56–60]. Our study provides strong support to the idea of TFRs heterogeneity. We show that TFRs can reside in follicular or extrafollicular spaces of LN tissues indicating that there may be spatial adaptations that could lead to TFRs heterogeneity. We also show that TFRs can inhibit TFHs in a cell-to-cell setup and can also produce IL-10 that has the potential to suppress cells that are spatially separated. Importantly, transcriptomic and epigenomic analysis identified FCRL3 and DPP4 that were subsequently validated as surface markers that delineates TFRs into four subpopulations three of which maybe functionally distinct.

FCRL3 is a cell surface protein expressed on a subset of Tregs, B cells, natural killer (NK), CD8^+^ T, and gamma delta T cells (gdT) [61–64]. It regulates plasma cell differentiation [38], B cell function [65] and inhibits Treg function [66]. DPP4, also known as CD26, is an extracellular protein that has been traditionally used as a marker of immune activation and effector functions in T cells [45]. However, to our knowledge, the functional role of FCRL3 and DPP4 expression in TFRs is unclear. Ours is the first report demonstrating that FCRL3 and DPP4 expression could reliably discriminate human TFRs from TFHs at the time of writing.

A series of validation experiments at protein level corroborated our Next-Generation Sequencing (NGS) data showing greater surface expression of DPP4 and FCRL3 on TFRs compared to TFH, Tregs and CD8^+^ T cells. Co-staining of DPP4 and FCRL3 showed that they are predominantly mutually exclusive, uncovering remarkable TFRs heterogeneity. Further analysis showed that these populations defined memory populations with potentially distinct differentiation lineages. Regulatory T cells expressing FCRL3 have been shown to exhibit a memory phenotype [67]. Consistently, we found that TFRs expressing FCRL3 had high CD27 and CD62L expression, denoting a memory phenotype. Furthermore, DPP4^+^FCRL3^−^TFRs displayed a high capacity to produce IL-2 and TNF-α, while the FCRL3^+^DPP4^−^TFRs displayed limited cytokine production capacity. Given these findings and observations from Swainson et al. [67] which demonstrated that FCRL3^+^ Tregs have reduced capacity to suppress the proliferation of effector T cells in humans, it is worth investigating if FCRL3^+^DPP4^−^TFRs also have limited suppressor cytokine secreting capacity. A more robust functional readout is needed to confirm that FCRL3 and DPP4 are functionally defining markers.

However, we did not have samples to sort these subsets and perform ddPCR, RNA-Seq or ATAC-Seq experiments. Clearly this is important observation that warrants further exploration.

Much about the role of TFRs in HIV pathogenesis remains unknown. We were unable to clearly determine the impact of HIV infection on TFR subsets due to low HIV viral loads in our clinical samples. Future elucidation of FCRL3^+^DPP4^−^, DPP4^+^FCRL3^−^, FCRL3^+^DPP4^+^ and FCRL3^−^DPP4^−^ TFRs function, differentiation and signalling will improve our understanding of immune tolerance and homeostasis and may create opportunities for the development of new therapeutic interventions in the HIV-1 disease settings. There is also need for more work to elucidate molecular mechanisms that drive TFRs expansion within the GCs, if and how the increased TFR frequencies inside GCs influence antibody production or TFH function.

## Conclusions

Improved understanding of TFRs has partly been hampered by the lack of suitable surface markers for distinguishing them from TFH and conventional Tregs. Key markers used to define TFRs such as CD25 and FOXP3 are also expressed by conventional Tregs, whereas CD127, PD-1 and CXCR5 are also not exclusive to this cell lineage. Here, we report two novel markers DPP4 and FCRL3 markers that define functionally distinct TFR subsets in human LNs. Our study also provides insight into the phenotype, localization and function of TFRs in the context of HIV-1 infection.

### Methods

#### Study population

We studied a total of 58 participants: 20 HIV uninfected and 38 HIV-infected individuals of which 31 were treatment initiators and 7 were chronic untreated. A subset of these were used for RNA-seq experiments selected based on the sample availability. The study participants were enrolled from the HIV Pathogenesis Programme (HPP) LN study cohort. Individuals were recruited from study sites based in the Umlazi Township, Durban, South Africa for an on-going cohort known as the Females Rising through Education, Support and Health (FRESH). The FRESH cohort was designed to identify early HIV infection by carrying out longitudinal follow-ups and frequent testing for HIV acquisition in a high-risk HIV negative study population [42]. Individuals identified in hyperacute infection were placed on ART soon after infection. Informed consent was obtained from all study participants prior to enrolment in the study. Excisional LNs and paired blood samples were obtained from the recruited study participants. Inguinal, axillary or cervical LNs were obtained alongside a paired blood sample of 120 ml from each study participant. Measurements of CD4 counts and viral loads were performed by Global Clinical and Viral laboratories (Durban, South Africa). Human ethics approval was obtained from the Biomedical Research Ethics (BREC) of the University of KwaZulu-Natal and the Institutional Review Board (IRB) of Massachusetts General Hospital.

#### Lymph nodes and peripheral blood samples

Excised LNs were divided into two pieces; approximately 1/3 of the LN was fixed in 10% formal-saline (Sigma-Aldrich, St Louis, Missouri, USA) for downstream microscopy studies. The 1/3 formal-saline fixed tissues were paraffin embedded by Lancet Laboratories (Durban, South Africa). Tissue blocks were thereafter stored and cut using a microtome (Leica) for staining on individual slides. The remaining 2/3 was mechanically processed to release lymph node mononuclear cells (LMCs) as described by Schacker et al*.*, *16*. In brief, the macerated LN was passed through a 70 uM cell strainer (BD, 352,350) into a collection tube containing R10 media (RPMI 1640 Supplemented with 10% heat-inactivated fetal calf serum [FCS], 2 mM L-glutamine, 50 U/ml of penicillin, 50 ug of streptomycin/ml, and 10 mM HEPES). Cells were pelleted and collected by centrifugation (1800 RPM, 6 min (min), room temperature (RT). Peripheral blood mononuclear cells (PBMCs) were isolated by HPPs core processing facility from patient blood samples by density-gradient centrifugation using Histopaque-1077 (Sigma-Aldrich). LNs and peripheral blood samples were obtained and processed on the same day and cryopreserved in freezing media (10% DMSO- FBS).

#### Flow cytometry analysis

Frozen LMCs and PBMCs were phenotypically and functionally characterized using multi-parameter flow cytometry. Briefly, for surface staining, cells were incubated for 20 min at RT in the dark in staining buffer [2% FCS in PBS buffer] containing the following antibodies: CD3-AF700-UCHT1 (BD, 557,943), CD4-BV650-SK3 (BD, 563,875), CD8-PE-RPA-T8 (BD, 555,367), CD25-PE-Cy5 -M-A251 (BD, 555,433), CD127-BV785-A019D5 (BioLegend, 351,329), CXCR5-AF488-RF8B2 (BD, 558,112) and PD-1- BV421-EH12.2H7 (BioLegend, 329,920), FCRL3-PE-H5/FcRL3 (BioLegend, 374,406), DPP4-PE-CY5-BA5b (BioLegend, 302,713), CD62L-BV711-SK11 (BioLegend, 565,040) and CD27-FITC-M-T271 (BioLegend, 356,404). Cells were also stained with live/dead fixable aqua cell viability dye (Invitrogen, L34957). For intracellular staining, cells were washed with staining buffer and incubated for 20 min at 4 °C with 4X transcription factor fix/perm buffer according to manufacturer’s instructions (BD, 51–9,008,100). Fixed and permeabilized cells were washed again with perm wash buffer (BD, 519,008,102) and incubated for 20 min at RT with perm wash buffer containing FOXP3- PE-CF594- 259D/CY (BD, 562,421), TNF-α-A700-Mab11 (BD, 557,996), IFN-γ-BV711-45.B3 (BD, 502,540) and IL-2-FITC (BD, 5344.111) antibodies. Fluorescence minus one (FMO) or unstained cells were used as a control. Cells were acquired on a LSRFortessa™ (Serial # H647794E6049, BD). Data was analysed using FlowJo software (Treestar FlowJo version 10).

#### Immunofluorescence (IF) microscopy

Multicolor immunofluorescence microscopy staining was conducted on 0.4 µM sections of formalin fixed paraffin embedded (FFPE) lymph nodes using the opal 4-colour fluorescent IHC kit (PerkinElmer, Waltham, MA, USA) according to manufacturer instructions with minor modifications. Briefly, following deparaffinization, rehydration and antigen retrieval, two blocking steps (2 × 10 min, RT) were performed using the Dako peroxidase blocking reagent (Agilent, S202386) and Bloxall block (Vector Laboratories, SP-6000). Slides were washed with Flex 20X wash buffer (Dako, K800721) for 5 min, followed by incubation with first primary antibody, BCL-6 (Dako, IR62561) for 30 min at RT, washed with wash buffer for 5 min, thereafter, probed with Opal polymer HRP (PerkinElmer, ARH1001EA) for 20 min at RT, washed with wash buffer twice (5 min) and detected using the Opal polymer 520 (10 min, RT) (PerkinElmer, FP1487). This procedure was repeated for the second and third antibodies CD4 (Dako, R64961) and FOXP3 (AbCam, ab22510) detected on Opal 570 (PerkinElmer, FP1488) and Opal 670 (PerkinElmer, FP1489), DPP4 (AbCam, ab222716) respectively. Slides were counterstained with spectral DAPI (PerkinElmer, FF1490) and mounted with Dako fluorescence mounting medium (Agilent, S3023). Images were acquired using the Axio Observer with TissueFAXS imaging software (TissueGnostics). Quantitative image analysis was conducted using TissueQuest (TissueGnostics).

#### Co-culture assays

A subset of five HIV-infected late-treated individuals were used to perform the co-culture assays. To increase cell numbers for the assay, we enriched for total CD4^+^CD25^−^CD127^+^ and CD4^+^CD25^+^CD127^−^. Briefly, LMCs were washed in R10 media then CD4^+^ T cells were negatively selected from the total LMCs followed by positive selection on anti-CD25 magnetic beads, separating CD4^+^CD25^−^CD127^+^(TFH) and CD4^+^CD25^+^CD127^−^(TFR) cells using the Regulatory T cell Separation Kit and AutoMACS (Miltenyi Biotech). Naïve B cells were isolated using the human naïve B cell enrichment kit according to manufacturer’s instructions (Stemcell technologies, Vancouver, Canada). Thereafter, the CD4^+^CD25^−^CD127^+^ (TFH) were co-cultured in a 96-well U-bottom plate with autologous naïve B cell populations in the presence or absence of CD4^+^CD25^+^CD127^−^ (TFR). IgG concentrations were determined in day 8 culture supernatants by ELISA.

#### Droplet digital PCR (ddPCR)

TFRs (CD4^+^CXCR5^+^PD-1^+^CD25^+^CD127^−^) and conventional CD4 T cells (CD4^+^CXCR5^−^PD-1^−^) were sorted on the FACS aria fusion (BD Biosciences). We sorted extra-follicular TFRs because follicular TFRs exist at very low frequency not amenable to FAC sorting. Total RNA was extracted from sorted populations using the QIAzol Lysis Reagent (Qiagen, Cat # 79,306) and RNeasy Mini Kit (Qiagen, Cat # 74,106) as per manufacturer’s instructions, and used for cDNA synthesis using the iScript cDNA synthesis kit (Bio-Rad, Cat # 1,708,891). The cDNA was used as a template for IL-10 (ThermoFischer Scientific, Assay ID: Hs00961622_m1) mRNA quantification by droplet digital PCR assays using pre-designed qPCR kits (ThermoFischer Scientific, 4,331,182) for FAM/MGB fluorescence. Briefly, PCR droplets were generated using QX200 Droplet Generator (Bio-Rad) from a master mix of cDNA, ddPCR supermix for probes (No dUTP) (Bio-Rad, 186–3025), pre-designed probes and droplet generation oil. PCR thermal cycling was conducted following optimized cycling conditions: an initial incubation at 95 °C for 10 min, 40 cycles of 94 °C for 30 s and 60 °C for 1 min, followed by a final incubation at 98 °C for 10 min and holding at 4 °C until reading time. After PCR amplification, droplets were measured in the QX200 ddPCR Droplet Reader, and target gene copy number was analyzed using QuantaSoft analysis software (Bio-Rad) and recorded as mRNA copies/20 μL. Absolute IL-10 mRNA counts were normalized to the expression of the housekeeping gene B2M (ThermoFischer Scientific, Assay ID: Hs00187842_m1).

#### Activation-induced marker (AIM) assay

LMCs were thawed, rested for 3 h and cultured in 96-well U-bottom plates at a concentration of 1 × 10^6^ cells using AIM-V medium (Thermo-fisher scientific). Cells were either left unstimulated or stimulated with HIV-1 peptide pools spanning the entire protein sequences for Gag, Nef and Env. Staphylococcal enterotoxin B (SEB, 0.5 μg/ml) (Sigma) was used as a positive control, while unstimulated cells were used as a negative control. Thereafter, cells were incubated for 18 h at 37 °C and 5% CO_2_. Following stimulation cells were stained for viability dye (aquavivid, Life Technologies) and surface markers (20 min, RT) and then fixed using 2% paraformaldehyde before acquisition at a LSRII flow cytometer (BD Biosciences). Background subtracted signal was calculated as the frequency of AIM^+^ cells stimulated with antigen minus the frequency unstimulated AIM^+^ cells.

#### ATAC-seq data processing and analysis

ATAC-Seq FASTQ files were analysed with FastQC and passed all quality checks. Paired-end sequences were trimmed with TrimGalore and mapped to the hg19 genome reference with Burrows-Wheeler Alignment (BWA) [68] and sorted with the Sequence Alignment/Map (SAMtools) [69]. The resulting BAM files were filtered for reads falling into known blacklisted regions. For each sample, reads were marked as duplicate with Picard Tools. Peaks for each subset were called using an intersection of the results from the Genrich and MACS2 peak callers [70]. A cut-off of 0.05 was chosen for FDR. Peak quantification was done by counting the reads falling into the total peak set. Downstream analysis was performed with DESeq2. Peak regions were annotated with the annotate Peak function from the ChIPseeker package 31.

#### RNA-seq sample preparation and sequencing

TFRs (CD4^+^CXCR5^+^PD-1^+^CD25^+^CD127^−^) and TFH cells (CD4^+^CXCR5^hi^PD-1^hi^) were sorted from LN samples of seven biological replicates (4 HIV positive and 3 HIV negative) on BD FACSAria II (BD Biosciences) with a sorting purity of ~ 99% (Additional file 4). Total RNA was isolated from lysed cells using QiagenRNeasy Mini columns (Qiagen, Valencia, CA) according to the manufacturer’s instructions. Purified RNA was quantified using TapeStation 2200 instrument (Agilent). Messenger RNA (mRNA) was isolated from total RNA using NEBNext oligo dT beads (New England Biolabs). The isolated mRNA was fragmented and thereafter reverse transcribed using NEBNext ultra RNA library preparation kit (New England Biolabs). cDNA products were purified using AmpureXP beads (Beckman Coulter, Danvers, MA) and indexed using NEBNext multiplex oligo (New England Biolabs). KAPA kit was used for final quantification of obtained cDNA libraries molarity for sequencing. Indexed libraries were pooled and sequenced using high throughput NextSeq 550 (Illumina, San Diego, CA).

#### RNA-seq data processing and analysis

RNA-Seq reads were quantified with the Kallisto package using the Ensembl 85 reference [71]. Differential expression analysis was performed with the Sleuth R package [33] on the aggregate transcripts for each gene. To account for heterogeneity among sample sources, the patient ID of each sample was used as a covariate in addition to cell subset for differential expression analysis. A q-value cut-off of 0.05 was considered statistically significant. The beta statistic was converted to a log base 2 value and used as a proxy for log-fold differences in gene expression between subsets. For visualization purposes, gene counts were transformed with the *vst* function of the DESeq2 package and inter-patient variation was removed using the *sva* and *limma* R packages [72–74]. Functional enrichment was performed with the *enrichGO* function of the *clusterProfiler* R package [75]. Visualization of results was done with the *ggplot* and *ComplexHeatmap* R-packages including custom scripts [76]. Lastly, a gene expression network was constructed with WGCNA [34] to determine TFR-centric and TFH-centric modules. Functional enrichment was calculated and GSEA analysis was performed between TFR and TFH for each module.

### Statistical analysis

Statistical analysis and graphical presentation were performed using GraphPad Prism version 7.0 software (GraphPad Software Inc., La Jolla, CA, USA). Mann–Whitney U test was utilized to compare differences between any two groups. Spearman’s Rank correlation was used to define the correlation between variables. Statistical analysis of significance was calculated using Kruskal Wallis test with Dunn’s post hoc analyses for multiple comparisons. Statistical significance was set at *p* < 0.05.

## Supplementary Information

**Additional file1.** Summary of clinical characteristics of study participants for DDPCR assay.

**Additional file2.** Antigen specificity of TFRs.

**Additional file3.** Summary of clinical characteristics of study participants for fre-quencies, localisation and functional characterization.

**Additional file4.** Gating strategy and sorting purity for TFRs and TFH cells.

**Additional file5.** Summary of clinical characteristics of study participants for NGS experiments.

**Additional file6.** List of differentially expressed genes.

**Additional file7.** List of differentially accessible genes.

**Additional file8.** TFRs subpopulation display a CD27hiCD62L+ phenotype.

**Additional file9. **HIV-infection does not modulate DPP4 and FCRL3 expression.

## Data Availability

The datasets generated and/or analysed during the current study are available in the NCBI's GEO repository, [ACCESSION NUMBER: GSE180532].

## Abbreviations

LN: Lymph node
GC: Germinal center
Tregs: Regulatory T cells
TFH: T follicular helper cells
TFRs: T follicular regulatory cells
CD40L: CD40 ligand
CXCR5: C-X-C chemokine receptor type 5
ICOS: Inducible CO-stimulator
PD-1: Programmed death-1
PD-L1: Programmed death-ligand 1
FOXP3: Forkhead box protein 3
BCL-6: B cell lymphoma 6
CTLA-4: Cytotoxic T-lymphocyte-associated protein 4
FACS: Fluorescent-activated cell sorting
NGS: Next generation sequencing
ATAC-Seq: Assay for transposase accessible chromatin using sequencing
TSS: Transcriptional start site
RNA-Seq: RNA-sequencing
QC: Quality control
PCA: Principal component analysis
TFs: Transcription factors
DE: Differentially expressed
WGCNA: Weighted gene correlated network analysis
GSEA: Gene set enrichment analysis
DPP4: Dipeptidyl-peptidase 4
FCRL3: FC receptor Like protein 3

## References

[1] Boasso A, Shearer GM, Chougnet C (2009). Immune dysregulation in human immunodeficiency virus infection: know it, fix it, prevent it?. J Intern Med.

[2] Stellbrink HJ, van Lunzen J (2001). Lymph nodes during antiretroviral therapy. Curr Opin Infect Dis.

[3] Crotty S (2011). Follicular helper CD4 T cells (TFH). Annu Rev Immunol.

[4] . !!! INVALID CITATION !!! (Pratama and Vinuesa 2014, Vinuesa et al. 2009).

[5] Lindqvist M, van Lunzen J, Soghoian DZ, Kuhl BD, Ranasinghe S, Kranias G (2012). Expansion of HIV-specific T follicular helper cells in chronic HIV infection. J Clin Investig.

[6] Chung Y, Tanaka S, Chu F, Nurieva RI, Martinez GJ, Rawal S (2011). Follicular regulatory T cells expressing Foxp3 and Bcl-6 suppress germinal center reactions. Nat Med.

[7] Li S, Folkvord JM, Kovacs KJ, Wagstaff RK, Mwakalundwa G, Rendahl AK (2019). Low levels of SIV-specific CD8+ T cells in germinal centers characterizes acute SIV infection. PLoS Pathog.

[8] Linterman MA, Pierson W, Lee SK, Kallies A, Kawamoto S, Rayner TF (2011). Foxp3+ follicular regulatory T cells control the germinal center response. Nat Med.

[9] Sage PT, Paterson AM, Lovitch SB, Sharpe AH (2014). The coinhibitory receptor CTLA-4 controls B cell responses by modulating T follicular helper, T follicular regulatory, and T regulatory cells. Immunity.

[10] Wing JB, Ise W, Kurosaki T, Sakaguchi S (2014). Regulatory T cells control antigen-specific expansion of Tfh cell number and humoral immune responses via the coreceptor CTLA-4. Immunity.

[11] Wollenberg I, Agua-Doce A, Hernandez A, Almeida C, Oliveira VG, Faro J (2011). Regulation of the germinal center reaction by Foxp3+ follicular regulatory T cells. J Immunol.

[12] Miles B, Miller SM, Folkvord JM, Kimball A, Chamanian M, Meditz AL (2015). Follicular regulatory T cells impair follicular T helper cells in HIV and SIV infection. Nat Commun.

[13] Lopez-Ocasio M, Buszko M, Blain M, Wang K, Shevach EM (2020). T follicular regulatory cell suppression of T follicular helper cell function is context-dependent in vitro. Front Immunol.

[14] Chowdhury A, Del Rio Estrada PM, Tharp GK, Trible RP, Amara RR, Chahroudi A (2015). Decreased T follicular regulatory Cell/T follicular helper cell (TFH) in simian immunodeficiency virus-infected rhesus macaques may contribute to accumulation of TFH in chronic infection. J Immunol.

[15] Wallin EF, Jolly EC, Suchanek O, Bradley JA, Espeli M, Jayne DR (2014). Human T-follicular helper and T-follicular regulatory cell maintenance is independent of germinal centers. Blood.

[16] Sage PT, Francisco LM, Carman CV, Sharpe AH (2013). The receptor PD-1 controls follicular regulatory T cells in the lymph nodes and blood. Nat Immunol.

[17] Wu H, Chen Y, Liu H, Xu LL, Teuscher P, Wang S (2016). Follicular regulatory T cells repress cytokine production by follicular helper T cells and optimize IgG responses in mice. Eur J Immunol.

[18] Colineau L, Rouers A, Yamamoto T, Xu Y, Urrutia A, Pham HP (2015). HIV-infected spleens present altered follicular helper T cell (Tfh) subsets and skewed B cell maturation. PLoS ONE.

[19] Miller SM, Miles B, Guo K, Folkvord J, Meditz AL, McCarter MD (2017). Follicular regulatory T cells are highly permissive to R5-tropic HIV-1. J Virol..

[20] Roider J, Maehara T, Ngoepe A, Ramsuran D, Muenchhoff M, Adland E (2018). High-frequency, functional HIV-specific T-follicular helper and regulatory cells are present within germinal centers in children but not adults. Front Immunol.

[21] Sayin I, Radtke AJ, Vella LA, Jin W, Wherry EJ, Buggert M (2018). Spatial distribution and function of T follicular regulatory cells in human lymph nodes. J Exp Med.

[22] Sage PT, Ron-Harel N, Juneja VR, Sen DR, Maleri S, Sungnak W (2016). Suppression by T(FR) cells leads to durable and selective inhibition of B cell effector function. Nat Immunol.

[23] Sage PT, Sharpe AH (2016). T follicular regulatory cells. Immunol Rev.

[24] Xie MM, Chen Q, Liu H, Yang K, Koh B, Wu H (2020). T follicular regulatory cells and IL-10 promote food antigen-specific IgE. J Clin Investig.

[25] Xie MM, Fang S, Chen Q, Liu H, Wan J, Dent AL (2019). Follicular regulatory T cells inhibit the development of granzyme B-expressing follicular helper T cells. JCI Insight..

[26] Maceiras AR, Almeida SCP, Mariotti-Ferrandiz E, Chaara W, Jebbawi F, Six A (2017). T follicular helper and T follicular regulatory cells have different TCR specificity. Nat Commun.

[27] Aloulou M, Carr EJ, Gador M, Bignon A, Liblau RS, Fazilleau N (2016). Follicular regulatory T cells can be specific for the immunizing antigen and derive from naive T cells. Nat Commun.

[28] Reiss S, Baxter AE, Cirelli KM, Dan JM, Morou A, Daigneault A (2017). Comparative analysis of activation induced marker (AIM) assays for sensitive identification of antigen-specific CD4 T cells. PLoS ONE.

[29] Sage PT, Sharpe AH (2015). T follicular regulatory cells in the regulation of B cell responses. Trends Immunol.

[30] Aloulou M, Fazilleau N (2019). Regulation of B cell responses by distinct populations of CD4 T cells. Biomed J.

[31] Laidlaw BJ, Lu Y, Amezquita RA, Weinstein JS, Vander Heiden JA, Gupta NT (2017). Interleukin-10 from CD4(+) follicular regulatory T cells promotes the germinal center response. Sci Immunol.

[32] León B, Bradley JE, Lund FE, Randall TD, Ballesteros-Tato A (2014). FoxP3+ regulatory T cells promote influenza-specific Tfh responses by controlling IL-2 availability. Nat Commun.

[33] Pimentel H, Bray NL, Puente S, Melsted P, Pachter L (2017). Differential analysis of RNA-seq incorporating quantification uncertainty. Nat Methods.

[34] Langfelder P, Horvath S (2008). WGCNA: an R package for weighted correlation network analysis. BMC Bioinform.

[35] Fellows E, Gil-Parrado S, Jenne DE, Kurschus FC (2007). Natural killer cell-derived human granzyme H induces an alternative, caspase-independent cell-death program. Blood.

[36] Wang H, Sun Q, Wu Y, Wang L, Zhou C, Ma W (2015). Granzyme M expressed by tumor cells promotes chemoresistance and EMT in vitro and metastasis in vivo associated with STAT3 activation. Oncotarget.

[37] Wang L, Li Q, Wu L, Liu S, Zhang Y, Yang X (2013). Identification of SERPINB1 as a physiological inhibitor of human granzyme H. J Immunol.

[38] Li FJ, Schreeder DM, Li R, Wu J, Davis RS (2013). FCRL3 promotes TLR9-induced B-cell activation and suppresses plasma cell differentiation. Eur J Immunol.

[39] Wing JB, Kitagawa Y, Locci M, Hume H, Tay C, Morita T (2017). A distinct subpopulation of CD25(-) T-follicular regulatory cells localizes in the germinal centers. Proc Natl Acad Sci USA.

[40] Guerra-Maupome M, Palmer MV, Waters WR, McGill JL (2019). Characterization of γδ T cell effector/memory subsets based on CD27 and CD45R expression in response to mycobacterium bovis infection. ImmunoHorizons.

[41] Wirth TC, Badovinac VP, Zhao L, Dailey MO, Harty JT (2009). Differentiation of central memory CD8 T cells is independent of CD62L-mediated trafficking to lymph nodes. J Immunol.

[42] Dong KL, Moodley A, Kwon DS, Ghebremichael MS, Dong M, Ismail N (2018). Detection and treatment of Fiebig stage I HIV-1 infection in young at-risk women in South Africa: a prospective cohort study. Lancet HIV.

[43] Kawamoto S, Maruya M, Kato LM, Suda W, Atarashi K, Doi Y (2014). Foxp3(+) T cells regulate immunoglobulin a selection and facilitate diversification of bacterial species responsible for immune homeostasis. Immunity.

[44] Suchard MS, Mayne E, Green VA, Shalekoff S, Donninger SL, Stevens WS (2010). FOXP3 expression is upregulated in CD4T cells in progressive HIV-1 infection and is a marker of disease severity. PLoS ONE.

[45] Barsheshet Y, Wildbaum G, Levy E, Vitenshtein A, Akinseye C, Griggs J (2017). CCR8(+)FOXp3(+) T(reg) cells as master drivers of immune regulation. Proc Natl Acad Sci USA.

[46] Cretney E, Xin A, Shi W, Minnich M, Masson F, Miasari M (2011). The transcription factors blimp-1 and IRF4 jointly control the differentiation and function of effector regulatory T cells. Nat Immunol.

[47] Getnet D, Grosso JF, Goldberg MV, Harris TJ, Yen HR, Bruno TC (2010). A role for the transcription factor Helios in human CD4(+)CD25(+) regulatory T cells. Mol Immunol.

[48] Gokhale AS, Gangaplara A, Lopez-Occasio M, Thornton AM, Shevach EM (2019). Selective deletion of Eos (Ikzf4) in T-regulatory cells leads to loss of suppressive function and development of systemic autoimmunity. J Autoimmun.

[49] Matoba T, Imai M, Ohkura N, Kawakita D, Ijichi K, Toyama T (2019). Regulatory T cells expressing abundant CTLA-4 on the cell surface with a proliferative gene profile are key features of human head and neck cancer. Int J Cancer.

[50] Xing S, Gai K, Li X, Shao P, Zeng Z, Zhao X (2019). Tcf1 and Lef1 are required for the immunosuppressive function of regulatory T cells. J Exp Med.

[51] Kerfoot SM, Yaari G, Patel JR, Johnson KL, Gonzalez DG, Kleinstein SH (2011). Germinal center B cell and T follicular helper cell development initiates in the interfollicular zone. Immunity.

[52] Qi H, Cannons JL, Klauschen F, Schwartzberg PL, Germain RN (2008). SAP-controlled T-B cell interactions underlie germinal centre formation. Nature.

[53] Shulman Z, Gitlin AD, Targ S, Jankovic M, Pasqual G, Nussenzweig MC (2013). T follicular helper cell dynamics in germinal centers. Science (New York, NY).

[54] Lim HW, Hillsamer P, Banham AH, Kim CH (2005). Cutting edge: direct suppression of B cells by CD4+ CD25+ regulatory T cells. J Immunol.

[55] Xie MM, Dent AL (2018). Unexpected help: follicular regulatory T cells in the germinal center. Front Immunol.

[56] Franceschini D, Paroli M, Francavilla V, Videtta M, Morrone S, Labbadia G (2009). PD-L1 negatively regulates CD4+CD25+Foxp3+ Tregs by limiting STAT-5 phosphorylation in patients chronically infected with HCV. J Clin Investig.

[57] Ito T, Hanabuchi S, Wang YH, Park WR, Arima K, Bover L (2008). Two functional subsets of FOXP3+ regulatory T cells in human thymus and periphery. Immunity.

[58] Nadkarni S, Mauri C, Ehrenstein MR (2007). Anti-TNF-alpha therapy induces a distinct regulatory T cell population in patients with rheumatoid arthritis via TGF-beta. J Exp Med.

[59] Radziewicz H, Dunham RM, Grakoui A (2009). PD-1 tempers Tregs in chronic HCV infection. J Clin Investig.

[60] Valmori D, Merlo A, Souleimanian NE, Hesdorffer CS, Ayyoub M (2005). A peripheral circulating compartment of natural naive CD4 Tregs. J Clin Investig.

[61] Bajpai UD, Swainson LA, Mold JE, Graf JD, Imboden JB, McCune JM (2012). A functional variant in FCRL3 is associated with higher Fc receptor–like 3 expression on T cell subsets and rheumatoid arthritis disease activity. Arthritis Rheum.

[62] Davis RS, Wang Y-H, Kubagawa H, Cooper MD. Identification of a family of Fc receptor homologs with preferential B cell expression. 2001;98(17):9772-7. 10.1073/pnas.171308498 PMC55528 11493702

[63] Nagata S, Ise T, Pastan I (2009). Fc receptor-like 3 protein expressed on IL-2 nonresponsive subset of human regulatory T cells. J Immunol.

[64] Polson AG, Zheng B, Elkins K, Chang W, Du C, Dowd P (2006). Expression pattern of the human FcRH/IRTA receptors in normal tissue and in B-chronic lymphocytic leukemia. Int Immunol.

[65] Davis RS (2007). Fc receptor-like molecules. Annu Rev Immunol.

[66] Agarwal S, Kraus Z, Dement-Brown J, Alabi O, Starost K, Tolnay M (2020). Human Fc receptor-like 3 inhibits regulatory T cell function and binds secretory IgA. Cell Rep.

[67] Swainson LA, Mold JE, Bajpai UD, McCune JM (2010). Expression of the autoimmune susceptibility gene FcRL3 on human regulatory T cells is associated with dysfunction and high levels of programmed cell death-1. J Immunol.

[68] Li H, Durbin R (2009). Fast and accurate short read alignment with Burrows-Wheeler transform. Bioinformatics (Oxford, England).

[69] Li H, Handsaker B, Wysoker A, Fennell T, Ruan J, Homer N (2009). The sequence alignment/map format and SAMtools. Bioinformatics (Oxford, England).

[70] Zhang Y, Liu T, Meyer CA, Eeckhoute J, Johnson DS, Bernstein BE (2008). Model-based analysis of ChIP-Seq (MACS). Genome Biol.

[71] Bray NL, Pimentel H, Melsted P, Pachter L (2016). Near-optimal probabilistic RNA-seq quantification. Nat Biotechnol.

[72] Leek JT, Johnson WE, Parker HS, Jaffe AE, Storey JD (2012). The sva package for removing batch effects and other unwanted variation in high-throughput experiments. Bioinformatics (Oxford, England).

[73] Love MI, Huber W, Anders S (2014). Moderated estimation of fold change and dispersion for RNA-seq data with DESeq2. Genome Biol.

[74] Ritchie ME, Phipson B, Wu D, Hu Y, Law CW, Shi W (2015). limma powers differential expression analyses for RNA-sequencing and microarray studies. Nucleic Acids Res.

[75] Yu G, Wang LG, Han Y, He QY (2012). clusterProfiler: an R package for comparing biological themes among gene clusters. OMICS.

[76] Gu Z, Eils R, Schlesner M (2016). Complex heatmaps reveal patterns and correlations in multidimensional genomic data. Bioinformatics (Oxford, England).

