# Supplementary material for: The impact of HIV infection on the frequencies, function, spatial localization and heterogeneity of T follicular regulatory cells (TFRs) within human lymph nodes
Source: BMC Immunol. 2022 Jul 1;23:34. doi: 10.1186/s12865-022-00508-1 (PMC9250173; doi:10.1186/s12865-022-00508-1)
Supplement: Supplementary file 1 — Additional file1. Summary of clinical characteristics of study participants for DDPCR assay. [file 12865_2022_508_MOESM1_ESM.docx]

**Additional file 1. Summary of clinical characteristics of study participants for DDPCR assay**

|  | **HIV negative** | **Treated** | **Untreated** |
| --- | --- | --- | --- |
| **n** | 4 | 8 | 5 |
| **Age (years)** | 21 (20-22) | 22 (21-25) | 26 (18-29) |
| **CD4 count (cells/ul)** | N/A | 986  (650-1290) | 511  (251-1229) |
| **Viral load (copies/ml)** | N/A | <20  (<20-130,000) | 22,000  (87-400,000) |
