# Supplementary material for: The impact of HIV infection on the frequencies, function, spatial localization and heterogeneity of T follicular regulatory cells (TFRs) within human lymph nodes
Source: BMC Immunol. 2022 Jul 1;23:34. doi: 10.1186/s12865-022-00508-1 (PMC9250173; doi:10.1186/s12865-022-00508-1)
Supplement: Supplementary file 2 — Additional file2. Antigen specificity of TFRs. [file 12865_2022_508_MOESM2_ESM.docx]

**Additional file 2. Antigen specificity of TFRs**


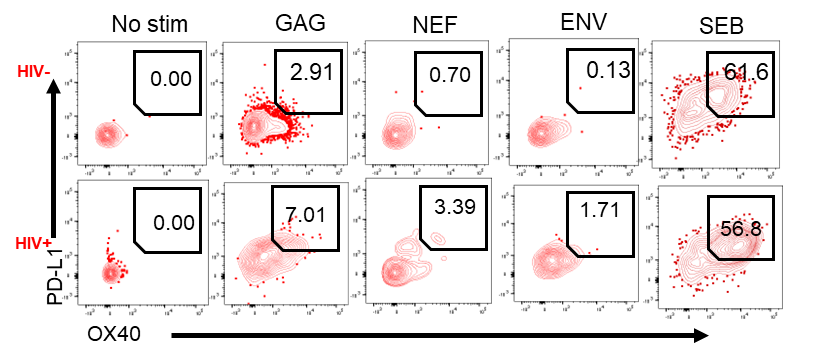

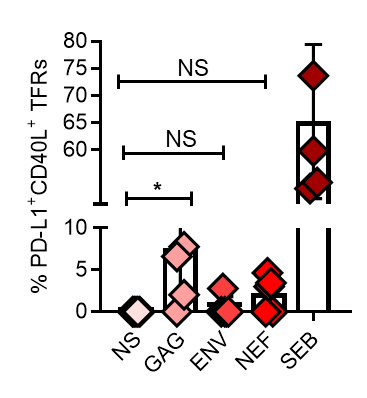


**(A)**

**(B)**

**Additional file 2. Antigen specificity of TFRs**

(**A**) Representative flow cytometry plots for HIV-uninfected and HIV-infected individual and (**B**) summary plot showing AIM+ TFRs identified as PD-L1+CD40L+ in HIV-infected subjects (n=5). Net frequency of the HIV-stimulated condition was calculated by subtracting the frequency detected in the unstimulated control. P values were determined using Mann-Whitney U test.
