# Supplementary material for: The impact of HIV infection on the frequencies, function, spatial localization and heterogeneity of T follicular regulatory cells (TFRs) within human lymph nodes
Source: BMC Immunol. 2022 Jul 1;23:34. doi: 10.1186/s12865-022-00508-1 (PMC9250173; doi:10.1186/s12865-022-00508-1)
Supplement: Supplementary file 3 — Additional file3. Summary of clinical characteristics of study participants for fre-quencies, localisation and functional characterization. [file 12865_2022_508_MOESM3_ESM.docx]

**Additional file 3. Summary of clinical characteristics of study participants for frequencies and functional characterization^ab^**

|  | **HIV negative** | **Treated** | **Untreated** |
| --- | --- | --- | --- |
| **n** | 8 | 15 | 7 |
| **Male** | 0 | 0 | 0 |
| **Female** | 8 | 15 | 7 |
| **Age (years)** | 20  (19-23) | 21  (18-25) | 26  (24-28) |
| **CD4 count (cells/ul)** | N/A | 782  (507-1248) | 856  (355-1229) |
| **Viral load (copies/ml)** | N/A | <20  (<20-55,000) | 1900  (<20-59,000) |
| **Treatment duration (days)** | N/A | 120  (8-926) | N/A |

^a 100% of participants were female^

^b Range values are reported in parentheses under median values^
