# Supplementary material for: The impact of HIV infection on the frequencies, function, spatial localization and heterogeneity of T follicular regulatory cells (TFRs) within human lymph nodes
Source: BMC Immunol. 2022 Jul 1;23:34. doi: 10.1186/s12865-022-00508-1 (PMC9250173; doi:10.1186/s12865-022-00508-1)
Supplement: Supplementary file 4 — Additional file4. Gating strategy and sorting purity for TFRs and TFH cells. [file 12865_2022_508_MOESM4_ESM.docx]

**Additional file 4. Gating strategy and sorting purity for TFRs and TFH cells**


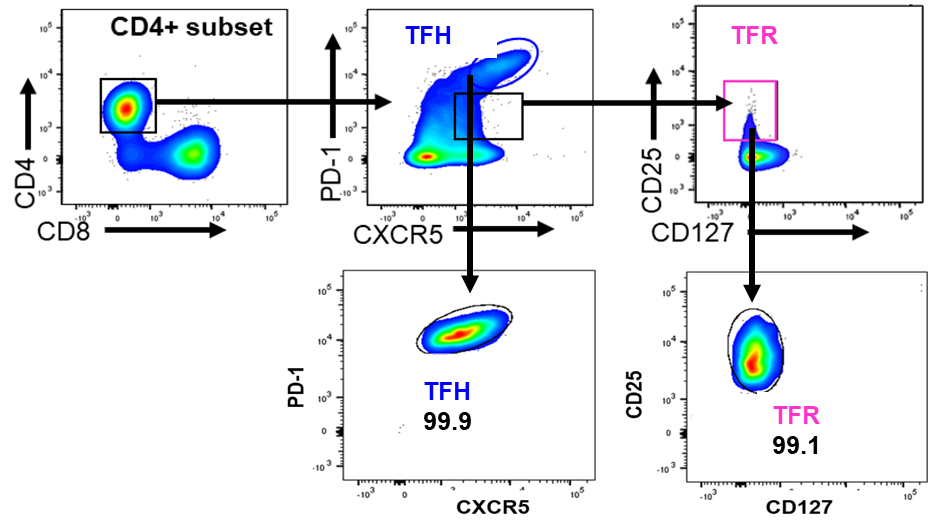


**Additional file 4. Gating strategy and sorting purity of TFRs and TFH cells.** TFRs were identified based on CD4^+^CXCR5^+^PD1^hi^CD127^-^CD25^+^ phenotypic markers and TFH cells were defined by CD4^+^CXCR5^hi^PD1^hi^ and sorted from LN samples on BD FACSAria II (BD Biosciences) with a sorting purity of ~99%.
