# Supplementary material for: The impact of HIV infection on the frequencies, function, spatial localization and heterogeneity of T follicular regulatory cells (TFRs) within human lymph nodes
Source: BMC Immunol. 2022 Jul 1;23:34. doi: 10.1186/s12865-022-00508-1 (PMC9250173; doi:10.1186/s12865-022-00508-1)

**Additional file 5. Summary of clinical characteristics of study participants for NGS experiments**


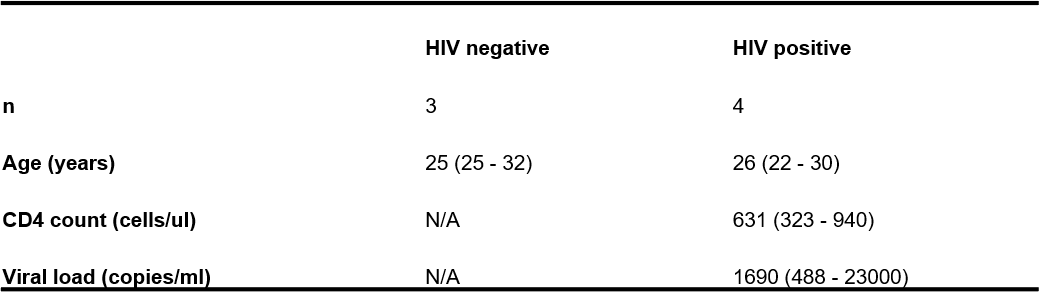

Supplement: Supplementary file 5 — Additional file5. Summary of clinical characteristics of study participants for NGS experiments. [file 12865_2022_508_MOESM5_ESM.docx]
