# Supplementary material for: The impact of HIV infection on the frequencies, function, spatial localization and heterogeneity of T follicular regulatory cells (TFRs) within human lymph nodes
Source: BMC Immunol. 2022 Jul 1;23:34. doi: 10.1186/s12865-022-00508-1 (PMC9250173; doi:10.1186/s12865-022-00508-1)
Supplement: Supplementary file 6 — Additional file6. List of differentially expressed genes. [file 12865_2022_508_MOESM6_ESM.docx]

**Additional file 6. List of differentially expressed genes.**

| **Gene Symbol** | **log2 FC** | **P-value** | **Q-value** | **Mean Counts** |
| --- | --- | --- | --- | --- |
| TOX2 | -2,6495215 | 8,9569E-26 | 1,8019E-21 | 3530,31207 |
| TOX | -1,6844706 | 4,5367E-25 | 4,5634E-21 | 2285,56122 |
| FCRL3 | 1,68968357 | 4,562E-24 | 3,0593E-20 | 2049,15279 |
| KCNK5 | -2,0457447 | 1,3512E-23 | 6,7957E-20 | 683,461502 |
| ITM2A | -1,8358424 | 2,2225E-23 | 8,9424E-20 | 1873,8083 |
| TCF7 | -1,8453658 | 4,811E-23 | 1,6131E-19 | 3033,24764 |
| GBP5 | 2,07678645 | 3,4954E-22 | 1,0046E-18 | 2865,35477 |
| NFATC1 | -1,5278097 | 1,0646E-20 | 2,6773E-17 | 2663,88446 |
| ATP1B1 | 2,65504496 | 9,2847E-20 | 2,0754E-16 | 252,326637 |
| NFATC2 | -1,5818569 | 1,1007E-19 | 2,2143E-16 | 1512,07243 |
| NCDN | -1,8562111 | 1,5057E-19 | 2,7538E-16 | 721,825919 |
| RHOB | -1,6625758 | 2,152E-19 | 3,6079E-16 | 1003,47797 |
| HAPLN3 | 1,6796655 | 8,0772E-19 | 1,25E-15 | 891,805765 |
| RNF19A | -1,4637548 | 9,5803E-19 | 1,3767E-15 | 1959,53239 |
| FGF23 | 1,74174439 | 1,1575E-18 | 1,5525E-15 | 900,28542 |
| PDCD1 | -3,9255371 | 3,8934E-18 | 4,8955E-15 | 673,979951 |
| IGFBP4 | -2,9520581 | 5,03E-18 | 5,9526E-15 | 584,451855 |
| TTN | 2,8768443 | 9,6869E-17 | 1,0827E-13 | 198,529055 |
| IL2RA | 2,3839716 | 1,9236E-16 | 2,0368E-13 | 1561,3721 |
| FCRL1 | 3,04984072 | 2,0406E-16 | 2,0527E-13 | 170,591577 |
| FKBP5 | -1,9683705 | 2,8041E-16 | 2,6863E-13 | 2563,3217 |
| SPON1 | 2,07609358 | 1,0564E-15 | 9,6601E-13 | 428,428289 |
| F5 | 1,46138121 | 1,1401E-15 | 9,9721E-13 | 1532,61492 |
| TMEM63A | 1,6119197 | 3,004E-15 | 2,5181E-12 | 627,256481 |
| CXCR5 | -1,8903579 | 9,3042E-15 | 7,1993E-12 | 3792,85259 |
| ST8SIA1 | -1,7630221 | 9,1838E-15 | 7,1993E-12 | 458,677536 |
| ICOS | -1,3533053 | 5,9327E-14 | 4,4205E-11 | 8791,18316 |
| KIAA0040 | 1,69558728 | 9,6834E-14 | 6,9575E-11 | 708,787909 |
| COTL1 | -2,3958749 | 1,0512E-13 | 7,2923E-11 | 4000,71801 |
| PLXND1 | -1,921426 | 1,4961E-13 | 1,0033E-10 | 335,35712 |
| SELP | 3,08718337 | 1,6076E-13 | 1,0433E-10 | 138,568123 |
| C16orf54 | -1,4228011 | 1,921E-13 | 1,2077E-10 | 822,978092 |
| TRPM2 | 1,54469169 | 3,6185E-13 | 2,206E-10 | 1371,03274 |
| ADTRP | 1,92853154 | 6,6448E-13 | 3,9318E-10 | 521,549674 |
| TNFSF8 | -2,3087651 | 1,0124E-12 | 5,8191E-10 | 712,058574 |
| CXXC11 | -9,421135 | 1,196E-12 | 6,6839E-10 | 13,0915818 |
| SLC12A6 | 1,45641491 | 1,5432E-12 | 8,3907E-10 | 1693,31881 |
| ZBTB10 | -1,2121172 | 2,4475E-12 | 1,2958E-09 | 4168,00256 |
| MXD4 | -1,4804214 | 2,5664E-12 | 1,3239E-09 | 544,989107 |
| SH2D1A | -1,8037848 | 3,5007E-12 | 1,7607E-09 | 318,390771 |
| CD82 | -1,5970377 | 3,604E-12 | 1,7684E-09 | 833,928083 |
| TXK | 1,69744528 | 4,0361E-12 | 1,9333E-09 | 384,544035 |
| TC2N | -1,1503142 | 4,6438E-12 | 2,1727E-09 | 2116,83336 |
| C9orf16 | -1,4077814 | 5,8919E-12 | 2,6939E-09 | 728,46818 |
| SEPN1 | -1,1953558 | 8,873E-12 | 3,9668E-09 | 2027,51509 |
| GSE1 | -1,774868 | 1,0918E-11 | 4,775E-09 | 418,283767 |
| RTKN2 | 2,56052204 | 1,1172E-11 | 4,7821E-09 | 304,034631 |
| TRAT1 | -1,7297923 | 3,2035E-11 | 1,3427E-08 | 309,905569 |
| MAP3K8 | -1,4173139 | 4,2273E-11 | 1,7356E-08 | 552,97404 |
| IKZF2 | 1,84315569 | 4,4344E-11 | 1,7842E-08 | 834,89176 |
| MAP3K1 | 1,21638846 | 6,536E-11 | 2,5782E-08 | 1561,28161 |
| SLC25A46 | -1,4150883 | 7,8507E-11 | 3,0373E-08 | 518,045692 |
| FLT3LG | 1,57269178 | 1,0931E-10 | 4,1491E-08 | 1095,18614 |
| POMT1 | -1,6590906 | 1,2268E-10 | 4,5706E-08 | 1710,54273 |
| THEM4 | 1,55475657 | 1,6805E-10 | 6,1471E-08 | 556,449309 |
| MT-ND4L | 1,08877928 | 1,9974E-10 | 7,1756E-08 | 6741,08974 |
| TRERF1 | -1,060637 | 2,559E-10 | 9,0321E-08 | 1783,24663 |
| PARP15 | 1,54936371 | 3,1516E-10 | 1,0932E-07 | 972,940416 |
| FOXP3 | 2,45978615 | 3,2992E-10 | 1,1062E-07 | 1387,94931 |
| ITPKB | -1,2502326 | 3,255E-10 | 1,1062E-07 | 12910,1858 |
| AHNAK | 1,15851187 | 3,425E-10 | 1,1296E-07 | 885,405313 |
| KIAA1671 | -2,0993986 | 3,6085E-10 | 1,1709E-07 | 591,475335 |
| MAP7 | -2,2338086 | 3,6802E-10 | 1,1752E-07 | 176,331826 |
| THEMIS | -2,4963043 | 4,6946E-10 | 1,4757E-07 | 359,343069 |
| PPP1CC | -1,159537 | 4,9225E-10 | 1,5236E-07 | 2374,42836 |
| CXCL13 | -8,9859382 | 5,7335E-10 | 1,7477E-07 | 11,2587057 |
| ITGB2 | -1,0077162 | 5,9529E-10 | 1,7875E-07 | 1990,36233 |
| IL1R1 | 3,38581496 | 6,4031E-10 | 1,8944E-07 | 400,972288 |
| TRIM8 | -1,7425556 | 6,5392E-10 | 1,9066E-07 | 658,472765 |
| HNRNPLL | -1,006021 | 7,4229E-10 | 2,1187E-07 | 2668,16321 |
| FBXO32 | -1,5956206 | 7,4774E-10 | 2,1187E-07 | 339,079271 |
| GNG4 | -7,3727271 | 8,2557E-10 | 2,3068E-07 | 49,5854542 |
| LDOC1L | -2,2867279 | 9,257E-10 | 2,5511E-07 | 530,322502 |
| RP11-47A8.5 | -2,1442291 | 1,0307E-09 | 2,8021E-07 | 181,730144 |
| ARHGAP5 | 1,80763018 | 1,6607E-09 | 4,4546E-07 | 828,251579 |
| HMGA1 | -1,0153993 | 1,9393E-09 | 5,1336E-07 | 1697,09037 |
| STX11 | -1,1603481 | 2,243E-09 | 5,8603E-07 | 1124,93663 |
| TPM2 | 1,61781273 | 2,5137E-09 | 6,4835E-07 | 293,86155 |
| RASGRP2 | 1,02318098 | 3,0461E-09 | 7,7572E-07 | 1348,10186 |
| NR3C2 | 2,12497606 | 3,2414E-09 | 8,1514E-07 | 219,098763 |
| CARD16 | 1,49073527 | 3,349E-09 | 8,3178E-07 | 681,235589 |
| GNA13 | -0,9567934 | 3,5727E-09 | 8,7652E-07 | 2292,32414 |
| LAYN | 2,08052096 | 3,9721E-09 | 9,6277E-07 | 268,840931 |
| CD55 | 1,4486243 | 4,1588E-09 | 9,9603E-07 | 445,311578 |
| MCL1 | -0,986284 | 4,6872E-09 | 1,1094E-06 | 6063,90462 |
| ICA1 | -1,0578777 | 5,2175E-09 | 1,2205E-06 | 1554,88182 |
| SAMHD1 | 1,40276182 | 6,7366E-09 | 1,5578E-06 | 1420,5894 |
| CD40LG | -1,9114124 | 9,3446E-09 | 2,1363E-06 | 668,101275 |
| MEGF6 | 1,25648598 | 1,0409E-08 | 2,3529E-06 | 531,645119 |
| PLCL1 | 1,67051722 | 1,1576E-08 | 2,5875E-06 | 685,814741 |
| CASK | 1,13133104 | 1,3442E-08 | 2,9716E-06 | 1853,82376 |
| VPS13C | 1,05663573 | 1,3707E-08 | 2,9973E-06 | 1901,15254 |
| SLC7A10 | -2,5276656 | 1,3964E-08 | 3,0022E-06 | 167,824976 |
| EGR1 | 1,56593241 | 1,4027E-08 | 3,0022E-06 | 809,759376 |
| RORA | 1,34627407 | 1,7349E-08 | 3,674E-06 | 387,605339 |
| MT1E | -1,6879748 | 1,8275E-08 | 3,8298E-06 | 734,103844 |
| NTRK3 | -7,3672921 | 1,9531E-08 | 4,012E-06 | 6,42477543 |
| GPA33 | 2,26783965 | 1,9543E-08 | 4,012E-06 | 143,229644 |
| IKZF3 | -0,9309562 | 2,0378E-08 | 4,1411E-06 | 1801,20314 |
| SH3KBP1 | -0,9215452 | 2,0849E-08 | 4,1944E-06 | 3077,18129 |
| TENM1 | -2,1601503 | 2,12E-08 | 4,2228E-06 | 747,653017 |
| ITGAL | -0,9137891 | 2,467E-08 | 4,8657E-06 | 2149,47518 |
| TLDC2 | 2,16528869 | 2,4914E-08 | 4,8662E-06 | 186,980272 |
| TNNT3 | 1,46231368 | 3,0729E-08 | 5,9444E-06 | 339,561019 |
| NEU1 | -1,1293691 | 3,165E-08 | 6,0641E-06 | 848,966252 |
| ASAP1 | -1,4053638 | 3,241E-08 | 6,1512E-06 | 340,281838 |
| SMCO4 | -2,7368885 | 3,5801E-08 | 6,7312E-06 | 112,566509 |
| GOLGA8B | 0,90340944 | 3,6735E-08 | 6,8308E-06 | 4341,26115 |
| LRMP | -1,1051888 | 3,701E-08 | 6,8308E-06 | 762,972692 |
| SLC9A3R1 | -1,0028005 | 3,9307E-08 | 7,1241E-06 | 1070,60382 |
| TP53INP2 | -1,2553064 | 3,9221E-08 | 7,1241E-06 | 517,75028 |
| CAST | 0,93273598 | 4,011E-08 | 7,2047E-06 | 1647,05949 |
| THADA | -1,3939127 | 4,3304E-08 | 7,7097E-06 | 1563,68989 |
| TBC1D27 | 2,73771073 | 4,4626E-08 | 7,8753E-06 | 110,476455 |
| SHISA2 | -6,3717892 | 4,706E-08 | 8,2327E-06 | 31,8233067 |
| SLC25A5 | -1,050913 | 5,2848E-08 | 9,1655E-06 | 793,353108 |
| SLFN5 | 0,96518108 | 5,7145E-08 | 9,8259E-06 | 1202,38531 |
| SGTB | 1,31044938 | 6,9044E-08 | 1,1771E-05 | 413,509557 |
| NMB | -1,8737774 | 8,0574E-08 | 1,3622E-05 | 181,278363 |
| KPNA2 | -1,2256701 | 8,5128E-08 | 1,4154E-05 | 459,184439 |
| PTPRCAP | -1,2370666 | 8,5056E-08 | 1,4154E-05 | 4288,82269 |
| PPP1R2P9 | 2,74638401 | 8,9169E-08 | 1,4704E-05 | 102,761552 |
| UPP1 | 1,55232946 | 9,1983E-08 | 1,5045E-05 | 253,655126 |
| CD8BP | -6,8399912 | 9,9894E-08 | 1,6207E-05 | 5,35169388 |
| SOCS2 | 4,30853264 | 1,0733E-07 | 1,7253E-05 | 53,0600949 |
| HLA-DPB1 | -1,3489137 | 1,0806E-07 | 1,7253E-05 | 406,674001 |
| KLF11 | 1,4123767 | 1,1614E-07 | 1,8397E-05 | 347,346366 |
| ZNF652 | 1,53837001 | 1,2291E-07 | 1,9318E-05 | 284,488187 |
| F2R | -3,0529303 | 1,2519E-07 | 1,9524E-05 | 107,05778 |
| RAB37 | -1,0019696 | 1,2691E-07 | 1,9639E-05 | 1204,87676 |
| NIN | -1,2008579 | 1,3008E-07 | 1,9976E-05 | 3026,05551 |
| KIAA1324 | -1,3144298 | 1,3147E-07 | 2,0038E-05 | 569,02657 |
| SAE1 | -1,0740154 | 1,5404E-07 | 2,3301E-05 | 711,156255 |
| DUSP6 | -2,9260313 | 1,6177E-07 | 2,4108E-05 | 370,229799 |
| TP53I13 | -1,0161446 | 1,6175E-07 | 2,4108E-05 | 899,653176 |
| H2AFV | -0,9573124 | 1,8238E-07 | 2,6979E-05 | 966,444744 |
| IDH2 | -0,9520824 | 1,875E-07 | 2,7534E-05 | 1026,16278 |
| IFITM1 | -1,1831358 | 2,1155E-07 | 3,0841E-05 | 4182,62721 |
| RP11-94L15.2 | -0,8444987 | 2,1703E-07 | 3,1411E-05 | 4478,25202 |
| CALR | -0,8401464 | 2,3629E-07 | 3,3955E-05 | 2870,00949 |
| RP11-87N24.3 | 1,68535706 | 2,5596E-07 | 3,6521E-05 | 8033,44987 |
| MYO15B | 1,28888962 | 2,6975E-07 | 3,8217E-05 | 766,807818 |
| FCRL6 | -6,3597552 | 2,8485E-07 | 4,0075E-05 | 4,53115115 |
| ZNF331 | -0,8326055 | 3,0518E-07 | 4,2636E-05 | 2457,18967 |
| RP11-53B2.2 | 1,1439938 | 3,4665E-07 | 4,8095E-05 | 466,043617 |
| SIK1 | -0,8582614 | 3,7251E-07 | 5,1329E-05 | 5897,82522 |
| MID1IP1 | -1,2725988 | 3,7581E-07 | 5,1433E-05 | 358,344578 |
| CTTN | -1,7919639 | 4,3607E-07 | 5,8878E-05 | 181,994756 |
| HS3ST3B1 | 0,86860399 | 4,3533E-07 | 5,8878E-05 | 1402,69302 |
| GZMH | -6,129085 | 4,6932E-07 | 6,2946E-05 | 4,18301307 |
| MS4A15 | -6,1285583 | 5,7055E-07 | 7,6015E-05 | 4,18224962 |
| ANKRD13A | -1,3238774 | 5,9993E-07 | 7,9404E-05 | 488,019539 |
| RP11-132N15.3 | -6,1278038 | 6,2323E-07 | 8,1949E-05 | 4,18115607 |
| INHBB | -6,0019415 | 6,2877E-07 | 8,214E-05 | 4,00269235 |
| FZD7 | -5,9948224 | 6,3287E-07 | 8,2143E-05 | 3,99282869 |
| ARHGEF11 | 2,35049708 | 6,5096E-07 | 8,3948E-05 | 188,999711 |
| PTPN11 | -1,0762303 | 6,9926E-07 | 8,9603E-05 | 519,110653 |
| KIAA1644 | -5,953957 | 7,133E-07 | 9,0824E-05 | 3,93667747 |
| ARAP2 | -1,5638794 | 7,2247E-07 | 9,1413E-05 | 454,624796 |
| BIRC3 | 1,10211826 | 7,6231E-07 | 9,5852E-05 | 830,922734 |
| FAM84B | 1,61902115 | 7,8891E-07 | 9,8579E-05 | 294,975596 |
| ARF5 | -1,0261776 | 8,521E-07 | 0,00010582 | 599,405663 |
| AC074289.1 | 1,89011699 | 8,9402E-07 | 0,00011034 | 148,45409 |
| OXNAD1 | 0,98642855 | 9,6783E-07 | 0,00011801 | 995,885222 |
| RP11-326C3.2 | -1,3849996 | 9,652E-07 | 0,00011801 | 1752,70592 |
| NR3C1 | -1,3703778 | 1,0567E-06 | 0,00012807 | 1839,29349 |
| GIMAP6 | -1,2456927 | 1,1179E-06 | 0,00013468 | 686,612844 |
| TSPAN14 | -0,9058611 | 1,1378E-06 | 0,00013626 | 947,412991 |
| SRGN | -0,9591623 | 1,1836E-06 | 0,0001409 | 12635,7447 |
| CAPZB | -0,9101846 | 1,2899E-06 | 0,00015176 | 1966,12042 |
| CD84 | -1,0471823 | 1,2887E-06 | 0,00015176 | 550,170219 |
| AC114730.3 | -5,7102007 | 1,3125E-06 | 0,00015351 | 3,61776914 |
| BZRAP1-AS1 | -1,0169755 | 1,3435E-06 | 0,00015624 | 613,531492 |
| CHUK | -1,0771413 | 1,4322E-06 | 0,0001656 | 477,021283 |
| KLHL2 | 0,99267493 | 1,6232E-06 | 0,00018661 | 725,438514 |
| PDE8A | 1,40230791 | 1,7193E-06 | 0,00019653 | 1011,52609 |
| AC017002.2 | 2,1690876 | 1,9184E-06 | 0,00021747 | 119,033267 |
| PPP4C | -0,8695216 | 1,9242E-06 | 0,00021747 | 1004,64362 |
| AL590867.1 | 1,23203072 | 2,086E-06 | 0,00023445 | 373,179875 |
| H2AFY | -0,8156691 | 2,1051E-06 | 0,00023528 | 1410,39034 |
| TNFSF9 | -6,2859526 | 2,1889E-06 | 0,00024254 | 15,7182886 |
| CD200 | -1,3010765 | 2,1942E-06 | 0,00024254 | 671,893073 |
| SUV420H2 | -1,2899064 | 2,5537E-06 | 0,00028074 | 482,425134 |
| PPP1R16B | -0,7743624 | 2,6093E-06 | 0,00028529 | 4578,22265 |
| CNKSR3 | -4,0900079 | 2,845E-06 | 0,00030938 | 45,837762 |
| RPL9 | 0,77681692 | 2,8719E-06 | 0,00031063 | 5170,20846 |
| DHRS7 | -1,201845 | 2,9743E-06 | 0,00031999 | 343,934207 |
| RP11-1399P15.1 | 2,75759211 | 3,008E-06 | 0,00032189 | 86,3686571 |
| GZMM | -1,5869196 | 3,1559E-06 | 0,00033593 | 861,006328 |
| GOLGA7 | -0,9653599 | 3,2867E-06 | 0,00034762 | 638,689736 |
| DNAAF2 | -1,0561037 | 3,3003E-06 | 0,00034762 | 466,174582 |
| MLKL | 1,08804858 | 3,3237E-06 | 0,00034826 | 484,182083 |
| NKX2-2 | -5,2035201 | 3,6162E-06 | 0,00037694 | 3,03513368 |
| CLCN4 | -6,1213415 | 3,8372E-06 | 0,00039762 | 6,16996713 |
| FFAR3 | -2,5533731 | 3,8541E-06 | 0,00039762 | 97,4341942 |
| TMEM155 | -6,2216402 | 3,8815E-06 | 0,00039841 | 4,31936761 |
| RP11-346D14.1 | 0,8747363 | 3,9935E-06 | 0,00040782 | 831,283313 |
| SPCS2 | -0,9378833 | 4,2285E-06 | 0,00042964 | 781,507318 |
| PPP2R5C | -0,742407 | 4,2606E-06 | 0,00043073 | 2584,36075 |
| MYO6 | -5,2186557 | 4,5553E-06 | 0,0004555 | 29,9440788 |
| PVALB | -6,1784923 | 4,5584E-06 | 0,0004555 | 9,66875584 |
| SLC52A2 | -1,237951 | 4,5736E-06 | 0,0004555 | 306,28204 |
| RP11-603B24.1 | 2,03519149 | 4,6279E-06 | 0,00045864 | 122,792757 |
| PFN1 | -0,9562927 | 4,8195E-06 | 0,00047341 | 15180,1544 |
| TKT | -0,8804209 | 4,824E-06 | 0,00047341 | 1294,52897 |
| MIAT | 1,4271164 | 4,9071E-06 | 0,00047885 | 211,869478 |
| JARID2 | -0,7838553 | 4,9509E-06 | 0,00047885 | 1419,89366 |
| SERF2 | -0,7355111 | 4,9331E-06 | 0,00047885 | 3134,80761 |
| MYL6 | -0,7449822 | 5,0065E-06 | 0,00048191 | 4469,37196 |
| PEG10 | -6,1706079 | 5,2451E-06 | 0,00050248 | 8,12055106 |
| AC114730.8 | -4,9315412 | 5,4457E-06 | 0,00051662 | 2,76210963 |
| INSM1 | -4,8942567 | 5,4694E-06 | 0,00051662 | 2,72664777 |
| CRTAM | -4,8939274 | 5,4697E-06 | 0,00051662 | 39,899999 |
| RN7SKP214 | -4,8949566 | 5,735E-06 | 0,00053914 | 2,72730926 |
| CHGB | -2,5764784 | 5,7925E-06 | 0,00053954 | 759,409291 |
| GAPDH | -0,8735358 | 5,7929E-06 | 0,00053954 | 11538,9455 |
| KB-1615E4.3 | -4,8235199 | 5,8888E-06 | 0,00054595 | 2,66061533 |
| PTPN7 | -0,8122571 | 6,1094E-06 | 0,0005638 | 1997,70913 |
| FGFR2 | -3,883368 | 6,3308E-06 | 0,00058156 | 103,159948 |
| CAV1 | -5,9854551 | 6,8052E-06 | 0,0006167 | 6,40953055 |
| NR4A3 | -1,5722668 | 6,7621E-06 | 0,0006167 | 2560,90462 |
| PPP6R2 | -0,7383301 | 6,7973E-06 | 0,0006167 | 2132,35225 |
| UQCR11 | 7,1499347 | 7,2808E-06 | 0,00065684 | 6,0865831 |
| INPP5F | 1,07320774 | 7,4186E-06 | 0,00066391 | 387,051543 |
| CTB-96E2.3 | -5,4228944 | 7,4582E-06 | 0,00066391 | 3,27489174 |
| CASP9 | -1,1636559 | 7,4306E-06 | 0,00066391 | 335,331014 |
| MTPN | -1,0040159 | 7,5391E-06 | 0,00066816 | 1180,46628 |
| RP11-713N11.4 | -4,7781257 | 7,5799E-06 | 0,00066882 | 2,61908492 |
| TOB2 | -0,9921905 | 7,6575E-06 | 0,00067272 | 1541,92874 |
| CDK2AP2 | -1,0025827 | 7,8801E-06 | 0,00068927 | 657,166572 |
| C1orf228 | -1,9880465 | 7,9592E-06 | 0,00069124 | 496,842227 |
| RP11-578F21.6 | -4,6975415 | 7,9713E-06 | 0,00069124 | 2,54695018 |
| ALMS1 | 1,05617094 | 8,0255E-06 | 0,00069295 | 746,736496 |
| ZNF274 | 1,08923295 | 8,3898E-06 | 0,00072131 | 381,490813 |
| GPR155 | 1,52236058 | 8,7781E-06 | 0,000744 | 934,250714 |
| OVOL1 | -5,3308395 | 8,7672E-06 | 0,000744 | 3,75665166 |
| SLC9A9 | -1,8752883 | 8,7598E-06 | 0,000744 | 157,305449 |
| HEY1 | -4,6813016 | 8,8017E-06 | 0,000744 | 2,53265539 |
| KSR2 | -2,0997215 | 9,0569E-06 | 0,00076237 | 111,528558 |
| MGAT4B | -0,8579244 | 9,1812E-06 | 0,00076961 | 817,717295 |
| PRDM1 | 1,66663982 | 9,3629E-06 | 0,00077803 | 154,873009 |
| SIK3 | -0,8556578 | 9,3976E-06 | 0,00077803 | 2196,37557 |
| TNFRSF13B | 1,96207123 | 9,3339E-06 | 0,00077803 | 183,683497 |
| MLLT3 | 1,01562357 | 9,5695E-06 | 0,00078901 | 481,654428 |
| PCDH1 | 2,14317851 | 1,0089E-05 | 0,00082845 | 188,290598 |
| ADRBK2 | 1,56960251 | 1,0222E-05 | 0,00083593 | 182,881788 |
| UGDH-AS1 | 0,96185487 | 1,0331E-05 | 0,00084148 | 617,700684 |
| IL2 | -4,4411549 | 1,0496E-05 | 0,00084919 | 2,33039974 |
| VN1R82P | -4,5206009 | 1,051E-05 | 0,00084919 | 2,39545623 |
| CCDC66 | 1,09416422 | 1,0738E-05 | 0,00086415 | 373,439251 |
| ADRM1 | -0,9064858 | 1,0797E-05 | 0,00086538 | 611,566572 |
| RBM15B | -0,7692306 | 1,1251E-05 | 0,00089275 | 1258,87392 |
| CSF2RB | 1,90483393 | 1,126E-05 | 0,00089275 | 246,889532 |
| ZAP70 | -0,7864415 | 1,1271E-05 | 0,00089275 | 8457,86947 |
| DGKH | 1,07759256 | 1,1477E-05 | 0,00090545 | 571,353525 |
| ULK1 | -0,8870083 | 1,2218E-05 | 0,00096019 | 745,936473 |
| GOLGA7B | 2,18240323 | 1,234E-05 | 0,00096594 | 182,729703 |
| GALNT2 | -1,1013537 | 1,2426E-05 | 0,00096894 | 621,405988 |
| LTBP3 | -0,7604552 | 1,2763E-05 | 0,00099134 | 3745,20535 |
| RNF5 | -1,0712127 | 1,3112E-05 | 0,00101454 | 430,878346 |
| RP11-173A8.2 | -4,3735465 | 1,4007E-05 | 0,00107969 | 2,27643021 |
| SLC26A1 | -1,6027479 | 1,4527E-05 | 0,00111548 | 204,993092 |
| GLIS1 | -4,3410287 | 1,5188E-05 | 0,00116179 | 2,25091931 |
| VAV3 | 1,06420879 | 1,5369E-05 | 0,00117116 | 383,414581 |
| RP11-60I3.5 | -4,276937 | 1,5477E-05 | 0,00117499 | 2,20147199 |
| INSIG1 | -1,3890404 | 1,555E-05 | 0,00117607 | 858,301644 |
| DR1 | -0,9304128 | 1,5645E-05 | 0,00117883 | 944,511214 |
| EPPK1 | 2,53299855 | 1,5747E-05 | 0,00118212 | 87,292109 |
| RASSF2 | -1,5564899 | 1,6053E-05 | 0,00120059 | 1239,80725 |
| TNF | -0,992513 | 1,6192E-05 | 0,00120648 | 443,668658 |
| GOLGA8A | 0,91843309 | 1,6258E-05 | 0,00120692 | 15997,322 |
| PKD2 | 1,2156214 | 1,6534E-05 | 0,00122291 | 278,427764 |
| BRK1 | -0,8101464 | 1,6925E-05 | 0,00124724 | 912,775861 |
| ITGA6 | 1,24689355 | 1,7166E-05 | 0,00126041 | 683,259398 |
| PCDHGB3 | -4,2524054 | 1,7262E-05 | 0,00126284 | 2,18283439 |
| SLC14A1 | 2,63424091 | 1,7514E-05 | 0,00127662 | 81,5156284 |
| P2RX5 | -1,4809528 | 1,7658E-05 | 0,00128244 | 871,550762 |
| ITGB7 | 0,97202967 | 1,7845E-05 | 0,00129135 | 692,113238 |
| SNORA66 | -4,2813836 | 1,83E-05 | 0,00131954 | 2,20486726 |
| TMED2 | -0,7857754 | 1,8597E-05 | 0,00133616 | 1118,2885 |
| RP11-863K10.7 | -4,2203701 | 1,8878E-05 | 0,00135153 | 2,15873338 |
| GYPC | -0,8686747 | 1,9439E-05 | 0,00138677 | 1269,11736 |
| AC131056.3 | -4,0781056 | 1,9709E-05 | 0,00140109 | 2,05487806 |
| ETFB | -1,1366871 | 2,0051E-05 | 0,00142034 | 512,173786 |
| RP11-55K22.5 | -4,1838473 | 2,0583E-05 | 0,00145295 | 2,13158066 |
| NEDD4 | 1,11517855 | 2,0724E-05 | 0,0014578 | 2519,36354 |
| TRIM60P18 | -4,2097127 | 2,1859E-05 | 0,00152711 | 2,15077459 |
| UBE2S | -1,105735 | 2,197E-05 | 0,00152711 | 442,910028 |
| STK39 | -1,2611547 | 2,1931E-05 | 0,00152711 | 740,474783 |
| ZNF703 | -5,4418019 | 2,2013E-05 | 0,00152711 | 93,1581369 |
| LL0XNC01-116E7.2 | -4,4410063 | 2,2148E-05 | 0,00153116 | 2,33027972 |
| UBTF | -0,7029036 | 2,3499E-05 | 0,00161905 | 3341,25167 |
| CSF1 | 1,42321812 | 2,3604E-05 | 0,00162071 | 192,484701 |
| PKM | -0,7045097 | 2,3892E-05 | 0,00163489 | 5418,9 |
| AGFG1 | -0,844547 | 2,4257E-05 | 0,00165422 | 757,615911 |
| ASCL2 | -5,5616703 | 2,4561E-05 | 0,00166929 | 15,3769608 |
| RP11-262H14.4 | -4,6277478 | 2,5076E-05 | 0,00167599 | 2,48608199 |
| SH3GL1P2 | -4,0994109 | 2,4825E-05 | 0,00167599 | 2,07010714 |
| TRAPPC5 | -1,2333895 | 2,5071E-05 | 0,00167599 | 268,164487 |
| PRELID1 | -0,936268 | 2,4893E-05 | 0,00167599 | 492,71258 |
| RGS1 | 1,2082814 | 2,5019E-05 | 0,00167599 | 7612,21524 |
| IL21 | -5,6492912 | 2,5686E-05 | 0,00170542 | 10,6104227 |
| TRABD2A | 1,19737204 | 2,5606E-05 | 0,00170542 | 306,306931 |
| GAL3ST2 | -5,4217433 | 2,5947E-05 | 0,00171709 | 5,16254493 |
| D2HGDH | -1,036359 | 2,6164E-05 | 0,00172578 | 601,907988 |
| MARCKSL1 | -1,6870197 | 2,7024E-05 | 0,00177669 | 422,329263 |
| PGAM1 | -0,7532275 | 2,7301E-05 | 0,00178904 | 1514,12093 |
| FAM179A | -3,1514739 | 2,7519E-05 | 0,0017975 | 59,1992715 |
| MIR4435-1HG | 0,84046166 | 2,7621E-05 | 0,0017983 | 706,944679 |
| RP4-784A16.5 | -4,0039186 | 2,789E-05 | 0,00180997 | 2,00271798 |
| GBP4 | 1,46007347 | 2,8309E-05 | 0,00183127 | 178,455174 |
| PI4KAP2 | -0,8166075 | 2,8521E-05 | 0,00183904 | 1927,8422 |
| TPR | 0,83277473 | 2,8846E-05 | 0,00185409 | 727,293135 |
| RP11-436G20.1 | -3,9951338 | 2,9547E-05 | 0,00189308 | 1,99662987 |
| HMCES | -0,7258077 | 2,9853E-05 | 0,00190663 | 1372,73729 |
| AC092669.3 | 1,0533919 | 3,0305E-05 | 0,00192938 | 341,588593 |
| PITPNC1 | -0,7642057 | 3,0531E-05 | 0,00193172 | 1012,98112 |
| AC026202.1 | -5,7979604 | 3,0534E-05 | 0,00193172 | 6,05339274 |
| DNAJB5 | -2,1668847 | 3,0725E-05 | 0,00193769 | 150,506831 |
| RP11-285F7.2 | 2,01664613 | 3,1067E-05 | 0,00194706 | 109,385224 |
| POU2AF1 | -1,9915769 | 3,1056E-05 | 0,00194706 | 110,919969 |
| GPR18 | -1,1218163 | 3,1615E-05 | 0,00197524 | 304,211806 |
| DZIP3 | -0,9624677 | 3,1717E-05 | 0,00197548 | 447,373911 |
| RP11-214K3.22 | -3,9445922 | 3,2236E-05 | 0,00200161 | 1,96196065 |
| CIB1 | -0,6876594 | 3,2575E-05 | 0,00201644 | 1809,1076 |
| CHST2 | -0,7406994 | 3,341E-05 | 0,00206181 | 1170,18983 |
| TBC1D17 | -0,774257 | 3,3521E-05 | 0,00206231 | 988,403973 |
| KCNQ1OT1 | 0,84556484 | 3,4495E-05 | 0,00211574 | 1442,92021 |
| AC009784.3 | -3,7821464 | 3,4653E-05 | 0,00211898 | 1,8545552 |
| SEMA4D | -1,1577993 | 3,4873E-05 | 0,00212597 | 5087,61106 |
| RP11-797A18.6 | -3,9152729 | 3,5148E-05 | 0,00213337 | 1,94212556 |
| RP11-665G4.1 | -3,8260279 | 3,5206E-05 | 0,00213337 | 1,88297516 |
| MBP | -0,8855547 | 3,6048E-05 | 0,0021778 | 547,953228 |
| PABPC4 | -0,6847535 | 3,6173E-05 | 0,00217882 | 1874,66712 |
| LDHD | -5,4522731 | 3,6413E-05 | 0,00218673 | 6,51071244 |
| RASSF3 | 1,11832381 | 3,7573E-05 | 0,00224967 | 292,206174 |
| HAL | -5,5573297 | 3,8404E-05 | 0,00229261 | 10,538743 |
| DAB1 | -5,2077735 | 3,9262E-05 | 0,00233691 | 4,80285872 |
| RP11-359G22.2 | -3,6618997 | 3,9758E-05 | 0,00235399 | 1,77885617 |
| ABHD17A | -0,731854 | 3,9783E-05 | 0,00235399 | 3765,66321 |
| HEYL | -4,2362327 | 4,0678E-05 | 0,00239987 | 91,5036819 |
| RP11-325F22.2 | 1,88253284 | 4,0805E-05 | 0,00240034 | 117,28429 |
| RAC2 | -0,6821689 | 4,178E-05 | 0,00245051 | 3257,00545 |
| CAPNS1 | -0,8117526 | 4,2682E-05 | 0,00249616 | 812,984788 |
| PRR5L | -4,7104694 | 4,284E-05 | 0,00249815 | 44,946684 |
| LZTS3 | 1,01959728 | 4,3865E-05 | 0,00251453 | 358,031431 |
| RP11-284N8.3 | 0,92434991 | 4,3612E-05 | 0,00251453 | 459,316029 |
| LINC00593 | -5,5437835 | 4,3868E-05 | 0,00251453 | 9,44104668 |
| LAT | -1,3099296 | 4,3599E-05 | 0,00251453 | 3185,35647 |
| IKZF4 | 1,49438307 | 4,3871E-05 | 0,00251453 | 162,682508 |
| CST7 | -0,9449423 | 4,3337E-05 | 0,00251453 | 690,828228 |
| IRF2BP2 | -0,9816388 | 4,4661E-05 | 0,00254531 | 22463,8487 |
| ZBTB7B | -1,1676301 | 4,4543E-05 | 0,00254531 | 891,879335 |
| JAK3 | -0,6737076 | 4,5252E-05 | 0,00257171 | 5044,55559 |
| CORO1B | -1,2276684 | 4,6166E-05 | 0,00261626 | 1601,9578 |
| H3F3AP4 | -1,21223 | 4,7153E-05 | 0,00266467 | 984,578603 |
| ZNF518B | -1,2939796 | 4,7412E-05 | 0,00267181 | 501,351852 |
| C19orf26 | -2,9082471 | 4,7971E-05 | 0,00269576 | 127,665437 |
| VDAC1 | -0,7955604 | 4,8923E-05 | 0,0027416 | 764,888056 |
| RP11-533E19.7 | -3,7897256 | 5,0587E-05 | 0,00282697 | 1,85943304 |
| FAM156B | -1,1131947 | 5,2036E-05 | 0,00289989 | 282,764755 |
| ADSL | -0,7734704 | 5,4177E-05 | 0,00301088 | 854,868315 |
| CCR8 | 1,94872338 | 5,4467E-05 | 0,00301864 | 289,270524 |
| KIAA0430 | 0,72190404 | 5,6696E-05 | 0,00313353 | 1429,70764 |
| UBE2E3 | -1,3788625 | 5,7072E-05 | 0,00314566 | 190,153859 |
| CTC-203F4.2 | -3,6767665 | 5,7363E-05 | 0,00315309 | 1,78804528 |
| GNAI2 | -0,7352851 | 5,7842E-05 | 0,00317077 | 6272,87908 |
| CTB-50E14.5 | -5,1968949 | 5,8707E-05 | 0,00320303 | 4,41462488 |
| GPR153 | -5,4325697 | 5,8749E-05 | 0,00320303 | 12,102028 |
| MIR221 | -3,6440006 | 5,9448E-05 | 0,00323238 | 1,76785545 |
| RP11-218M11.1 | -3,6104994 | 6,1729E-05 | 0,00334735 | 1,74744823 |
| STAM | 0,93335761 | 6,1913E-05 | 0,00334829 | 438,929676 |
| AKAP17A | -0,6860816 | 6,383E-05 | 0,00343349 | 3806,36291 |
| RBM42 | -0,7725644 | 6,3693E-05 | 0,00343349 | 814,825381 |
| BLK | -1,8774061 | 6,4234E-05 | 0,003446 | 248,764233 |
| TP53INP1 | -0,8163494 | 6,6553E-05 | 0,00356091 | 2908,28005 |
| CC2D1B | -0,7460457 | 6,7642E-05 | 0,00360959 | 1448,1272 |
| DRAP1 | -0,7645655 | 6,785E-05 | 0,00361113 | 894,871428 |
| KIAA2013 | -0,8861068 | 6,8534E-05 | 0,00363789 | 686,477771 |
| ATHL1 | -1,2233834 | 6,9006E-05 | 0,00365332 | 7372,13464 |
| AC017002.1 | 0,9526802 | 7,2951E-05 | 0,00384786 | 388,369875 |
| ATP6V0E1 | -0,9060661 | 7,3063E-05 | 0,00384786 | 929,537761 |
| MT-TC | 1,05427575 | 7,381E-05 | 0,00386695 | 360,611334 |
| ERCC5 | 0,74119889 | 7,3759E-05 | 0,00386695 | 928,819071 |
| C19orf43 | -0,636996 | 7,4425E-05 | 0,00387898 | 2732,87625 |
| SBNO2 | -0,6884767 | 7,4352E-05 | 0,00387898 | 1555,52108 |
| TPD52L2 | -0,7407581 | 7,6442E-05 | 0,00397382 | 1204,41614 |
| LRRC37B | 1,05230563 | 7,7312E-05 | 0,00400865 | 306,674935 |
| SEPT1 | -0,6494046 | 7,9533E-05 | 0,00410872 | 2370,80146 |
| CMIP | -0,7953869 | 7,965E-05 | 0,00410872 | 714,772099 |
| CTB-133G6.1 | 0,97294904 | 8,0117E-05 | 0,00411191 | 365,193625 |
| HIF1A | -0,7717915 | 8,0121E-05 | 0,00411191 | 785,19072 |
| AC090587.5 | -4,7556479 | 8,082E-05 | 0,00413723 | 3,77592817 |
| NKG7 | -5,4076648 | 8,1144E-05 | 0,00414328 | 12,5062513 |
| TMEM50A | -0,749872 | 8,1463E-05 | 0,00414902 | 865,076449 |
| RALGAPA1P | -3,8843919 | 8,4546E-05 | 0,00425468 | 1,92145071 |
| REPIN1 | -0,8153624 | 8,4543E-05 | 0,00425468 | 652,030652 |
| CTSB | -1,0059277 | 8,4595E-05 | 0,00425468 | 763,235902 |
| SOCS1 | -1,1370935 | 8,3758E-05 | 0,00425468 | 540,13733 |
| KDM6B | -0,6762065 | 8,4389E-05 | 0,00425468 | 1520,60231 |
| GFOD1 | -2,6217215 | 8,6634E-05 | 0,00434637 | 176,91473 |
| ARL6IP1 | -0,8417542 | 8,8271E-05 | 0,0044175 | 579,44956 |
| RPS6KA2 | 1,39499305 | 9,1002E-05 | 0,00454288 | 175,167984 |
| MVD | -0,8345466 | 9,3024E-05 | 0,00463233 | 650,916281 |
| RP11-466A19.8 | -3,3227585 | 9,5322E-05 | 0,00473502 | 1,58159393 |
| MUC20 | 1,23501573 | 9,5774E-05 | 0,00474577 | 232,068362 |
| SIAH2 | -1,3502944 | 9,6345E-05 | 0,00476235 | 234,355747 |
| LINC00152 | 0,88766905 | 9,8756E-05 | 0,00486957 | 450,979624 |
| RP1-272L16.1 | -3,3101178 | 0,00010197 | 0,00500369 | 1,57468023 |
| ATG2A | -0,8087402 | 0,00010192 | 0,00500369 | 1674,15954 |
| IL6R | -0,8787854 | 0,00010271 | 0,00501545 | 458,57015 |
| SREBF2 | -0,7352125 | 0,00010263 | 0,00501545 | 854,099876 |
| EVI2B | -0,6440097 | 0,00010386 | 0,00505918 | 1755,69906 |
| LEF1 | 0,87061465 | 0,00010473 | 0,0050894 | 1642,04332 |
| CD4 | -1,1220541 | 0,00010511 | 0,00509556 | 1522,10652 |
| ASF1A | -1,1165057 | 0,00010576 | 0,00510212 | 281,721459 |
| MT2A | -1,4134023 | 0,00010562 | 0,00510212 | 2420,32851 |
| RP11-553D4.2 | -3,4137607 | 0,00010729 | 0,00516356 | 1,63227069 |
| RP5-875H3.2 | -3,4656691 | 0,0001081 | 0,0051905 | 1,66190114 |
| RHOA | -0,7154558 | 0,00010975 | 0,00525717 | 2029,10228 |
| SLC47A2 | -5,0540781 | 0,00011324 | 0,00538435 | 6,02759488 |
| TRMT112 | -0,6956735 | 0,00011348 | 0,00538435 | 1097,40611 |
| TEAD1 | -5,2582762 | 0,00011333 | 0,00538435 | 9,21122251 |
| RP11-53B2.3 | 1,21581514 | 0,00011301 | 0,00538435 | 214,834667 |
| UAP1L1 | 1,21060812 | 0,00011478 | 0,00543341 | 219,790715 |
| USP36 | -0,9666927 | 0,00011507 | 0,00543401 | 1322,05079 |
| HTRA1 | -5,1229657 | 0,00011651 | 0,00548312 | 6,72374989 |
| FAM184A | 1,02928028 | 0,00011665 | 0,00548312 | 326,832055 |
| SNX9 | 0,62364602 | 0,00011812 | 0,00553913 | 2803,75537 |
| MT-TP | 1,25964684 | 0,00012054 | 0,00563982 | 464,389046 |
| DENND2D | -0,7657254 | 0,00012218 | 0,00570328 | 715,014463 |
| MFGE8 | 1,19887849 | 0,0001263 | 0,00588181 | 245,281565 |
| ZNF33A | 0,84756028 | 0,00012691 | 0,00588283 | 583,513534 |
| PELI1 | 0,79905204 | 0,00012668 | 0,00588283 | 585,422998 |
| GTDC1 | -0,9658734 | 0,00012906 | 0,00596864 | 418,604698 |
| NDUFA6 | -0,8507913 | 0,00012947 | 0,00597412 | 670,599183 |
| DPP4 | 3,12966393 | 0,00013005 | 0,00598724 | 51,6507755 |
| SETD7 | 1,72812276 | 0,00013427 | 0,00616729 | 349,405975 |
| ENPP7P4 | -3,2537105 | 0,00013506 | 0,00618945 | 1,5441953 |
| CISH | 1,59741046 | 0,00013595 | 0,00621603 | 135,643009 |
| RP11-334J6.6 | -4,5672731 | 0,00013783 | 0,00628747 | 3,44182481 |
| RP11-448G15.3 | 1,43479188 | 0,00013931 | 0,0063287 | 155,597759 |
| LRRC8D | -0,9013141 | 0,00013936 | 0,0063287 | 398,872863 |
| KIAA0922 | 0,87768361 | 0,00014015 | 0,00635016 | 426,179525 |
| EPB41L3 | -4,124205 | 0,00014181 | 0,00641095 | 2,74722387 |
| PASK | -1,3578167 | 0,00014242 | 0,00642401 | 2032,70217 |
| EPB41 | 0,65496896 | 0,00014482 | 0,00651669 | 4572,62998 |
| SEPT9 | -0,9563552 | 0,00014512 | 0,00651669 | 3036,53599 |
| LLGL1 | -0,7259904 | 0,00014599 | 0,00654142 | 865,455804 |
| PDGFD | -4,9715648 | 0,0001474 | 0,00658981 | 5,49581081 |
| PPP2CA | -1,0228285 | 0,00014884 | 0,00663929 | 840,816126 |
| SLC36A4 | -1,4492516 | 0,00015118 | 0,00672899 | 183,645486 |
| CDIPT | -0,7870683 | 0,00015222 | 0,00676032 | 819,8998 |
| LIMS1 | -1,0147257 | 0,0001532 | 0,00678878 | 775,628888 |
| NDRG3 | -0,8938438 | 0,00015517 | 0,00686078 | 425,899486 |
| RILPL2 | -1,1179759 | 0,00015596 | 0,00688063 | 1271,59951 |
| H3F3B | -0,8599725 | 0,00015708 | 0,00689998 | 11562,2042 |
| PTPN13 | -3,9630707 | 0,00015687 | 0,00689998 | 540,231935 |
| TMEM63C | -3,6838093 | 0,00015812 | 0,00693049 | 2,08603513 |
| LINC00893 | 1,25437276 | 0,00016284 | 0,00712192 | 194,194674 |
| ADAMTS7 | -5,1156208 | 0,00016589 | 0,00723957 | 7,56804597 |
| CEACAM4 | 6,21606164 | 0,00016813 | 0,0073211 | 6,89009883 |
| RPLP0 | -0,6745456 | 0,00016897 | 0,0073421 | 8525,87394 |
| CLNK | 1,11393568 | 0,00017042 | 0,007389 | 413,6023 |
| ERAP2 | 0,85776206 | 0,00017207 | 0,00741254 | 524,842987 |
| AP5B1 | -0,8857252 | 0,00017195 | 0,00741254 | 801,510843 |
| RP11-279F6.3 | -5,0586742 | 0,00017175 | 0,00741254 | 14,177366 |
| LRRC41 | -0,6871138 | 0,00017699 | 0,00760841 | 1029,37717 |
| RP11-686D22.8 | -4,7966987 | 0,00017853 | 0,00764171 | 5,02464241 |
| NR4A2 | -0,7998887 | 0,00017833 | 0,00764171 | 8570,61406 |
| LASP1 | -0,8527405 | 0,00018324 | 0,00782664 | 1738,8049 |
| TERF2IP | -0,6894607 | 0,00018391 | 0,0078386 | 1402,31562 |
| RRM1 | 0,98046388 | 0,00018473 | 0,00784034 | 336,029286 |
| BAK1 | -1,0188734 | 0,00018434 | 0,00784034 | 915,253043 |
| ANXA1 | -2,644985 | 0,00018555 | 0,00785855 | 538,013021 |
| CCL5 | -4,6273708 | 0,00018831 | 0,00794877 | 155,408301 |
| GADD45G | -1,5047149 | 0,00018847 | 0,00794877 | 402,438829 |
| B3GNT2 | -0,6440919 | 0,00019459 | 0,00818991 | 1344,64822 |
| IGHV1OR15-1 | -1,9157283 | 0,00019709 | 0,0082778 | 103,366688 |
| ANKRD34B | -5,1091667 | 0,00019921 | 0,0083494 | 2,93748879 |
| HDAC5 | -1,4932828 | 0,00020004 | 0,00836656 | 364,168539 |
| MPHOSPH8 | 0,75129991 | 0,00020232 | 0,00844437 | 646,909353 |
| ACTB | -0,8037556 | 0,00020344 | 0,00847379 | 16990,8999 |
| LINC00892 | -4,3629353 | 0,0002067 | 0,00857823 | 60,8264155 |
| FRK | 1,10268522 | 0,0002068 | 0,00857823 | 236,675526 |
| IKZF1 | -0,6121367 | 0,0002073 | 0,00858131 | 4835,83178 |
| MAF1 | -1,147973 | 0,00020855 | 0,00860116 | 1122,77751 |
| FIS1 | -0,7772421 | 0,00020864 | 0,00860116 | 636,14702 |
| BHLHE40 | -1,624286 | 0,00020924 | 0,00860848 | 427,342232 |
| STK38 | 0,67141966 | 0,00020992 | 0,00861883 | 1038,0939 |
| ARPC4 | -0,6471856 | 0,00021252 | 0,00870784 | 1274,03516 |
| DNHD1 | 1,04627966 | 0,00021311 | 0,00871411 | 848,192725 |
| RN7SL650P | -4,0349896 | 0,00021646 | 0,0088083 | 2,62063831 |
| PLCL2 | -0,6745474 | 0,00021673 | 0,0088083 | 1032,24783 |
| SLC39A10 | 1,2334268 | 0,00021629 | 0,0088083 | 273,306826 |
| AC018866.1 | -3,0450177 | 0,00021727 | 0,00881251 | 1,43645104 |
| AC005523.2 | 6,53400504 | 0,00021795 | 0,00882255 | 7,78926034 |
| TMSB10 | 0,6447085 | 0,00021854 | 0,00882833 | 6734,46695 |
| SDCBP | -0,6965669 | 0,00022137 | 0,00892505 | 1719,79983 |
| CHRM3-AS2 | 0,85121382 | 0,00022243 | 0,00894955 | 893,676117 |
| PPP1CA | -1,0814533 | 0,00022921 | 0,00920407 | 1156,3417 |
| ANAPC11 | -1,1803333 | 0,00023047 | 0,00923607 | 271,35757 |
| LRRC32 | 2,38106583 | 0,00023753 | 0,0095002 | 1193,98086 |
| TAB3 | -0,6237743 | 0,00023948 | 0,00955922 | 1504,21453 |
| WIPF1 | -0,5875632 | 0,00024427 | 0,00971176 | 2956,95966 |
| CPT1A | -1,8509369 | 0,00024381 | 0,00971176 | 105,167321 |
| PPP5C | -0,8564559 | 0,00024752 | 0,00980224 | 444,738179 |
| SMIM12 | -0,9327998 | 0,00024751 | 0,00980224 | 589,141572 |
| VCP | -0,6218957 | 0,00025506 | 0,01008121 | 1586,2452 |
| APBA2 | -1,4510323 | 0,00025649 | 0,01011793 | 914,931015 |
| TRGV10 | -4,9980166 | 0,00025796 | 0,01015567 | 10,3553293 |
| CCDC141 | 1,3147742 | 0,00026762 | 0,01050846 | 359,059516 |
| COA1 | -1,0228821 | 0,00026796 | 0,01050846 | 357,442986 |
| RELB | -0,8255965 | 0,00026906 | 0,01053098 | 686,829795 |
| FAS | 0,73417692 | 0,00027213 | 0,01063069 | 657,335285 |
| METTL9 | -0,9249597 | 0,00027634 | 0,01077404 | 335,572237 |
| SNHG1 | 0,65390211 | 0,00027814 | 0,01078167 | 1213,85899 |
| ECI1 | -1,0168632 | 0,00027766 | 0,01078167 | 285,093359 |
| GAS6 | -0,9974201 | 0,00027737 | 0,01078167 | 293,1238 |
| SLC25A3 | -0,5918639 | 0,00028147 | 0,0108895 | 4080,32059 |
| FOXN3 | 0,92217337 | 0,00028215 | 0,01089513 | 337,28442 |
| AC008948.1 | -4,2665505 | 0,00028398 | 0,0109446 | 2,67681111 |
| IL10RA | 0,91422716 | 0,00028456 | 0,01094588 | 3253,58789 |
| REEP4 | -1,0380153 | 0,0002865 | 0,01099962 | 356,748203 |
| ZNF428 | -1,2364871 | 0,00029157 | 0,01117308 | 233,779192 |
| GIMAP4 | -0,8544035 | 0,00029242 | 0,01118424 | 407,068352 |
| PTPN14 | -3,6243686 | 0,000295 | 0,01126139 | 77,5385404 |
| MTPAP | 0,71074525 | 0,00029576 | 0,01126928 | 926,923268 |
| PSTPIP1 | -0,613042 | 0,00029752 | 0,0113148 | 1724,19207 |
| TMEM59 | -0,6860707 | 0,00029823 | 0,01132052 | 1049,56237 |
| SGPP2 | -0,7306811 | 0,00029896 | 0,01132663 | 872,765224 |
| HDAC9 | 1,54786523 | 0,00030266 | 0,01144524 | 155,35733 |
| MTND1P23 | 0,58014477 | 0,00030491 | 0,01150885 | 3721,11807 |
| MLLT4 | 2,07589452 | 0,00030918 | 0,01162269 | 188,198783 |
| MT1X | -0,9113652 | 0,00030966 | 0,01162269 | 1472,28961 |
| RNPC3 | 0,95180115 | 0,00030861 | 0,01162269 | 316,144768 |
| RP11-436I9.5 | -4,1365387 | 0,00031176 | 0,01167974 | 2,96453639 |
| PSMD13 | -0,9293119 | 0,00031517 | 0,01178556 | 1155,84665 |
| FAM110A | -1,3451282 | 0,00032017 | 0,01192806 | 337,352211 |
| CRMP1 | -4,8885963 | 0,0003196 | 0,01192806 | 19,2934675 |
| BCL6 | -1,2609171 | 0,00032459 | 0,01207035 | 803,220164 |
| TMCO1 | -0,9394643 | 0,00032826 | 0,01218434 | 511,501833 |
| FBLN7 | -0,6411727 | 0,00032923 | 0,0121978 | 1221,09311 |
| AP001189.4 | 2,19797114 | 0,00033069 | 0,01220705 | 104,09448 |
| RMND5B | -0,8160346 | 0,00033044 | 0,01220705 | 566,245871 |
| LAMP1 | -0,7642063 | 0,00033411 | 0,01228799 | 2559,98053 |
| SAMD9 | 0,65406236 | 0,00033396 | 0,01228799 | 996,546523 |
| APOBEC3C | -0,7231053 | 0,00033783 | 0,01240235 | 755,312805 |
| EMID1 | -4,7617042 | 0,00033897 | 0,01242137 | 13,3998896 |
| SLC7A5 | -1,4160251 | 0,0003407 | 0,01246234 | 3646,75185 |
| FYB | -0,6390556 | 0,00034464 | 0,0125405 | 1853,02781 |
| ATP6AP2 | -0,8538059 | 0,00034443 | 0,0125405 | 419,634428 |
| FLT4 | 1,86387672 | 0,00034471 | 0,0125405 | 180,654471 |
| GDE1 | -1,0183342 | 0,00035584 | 0,01292191 | 294,579382 |
| SLC25A6 | -0,5953733 | 0,0003638 | 0,01318738 | 3170,90103 |
| ASB1 | -1,2141422 | 0,00036532 | 0,01321848 | 305,872161 |
| ZFP36L2 | -0,7622305 | 0,00036949 | 0,01334531 | 16427,8132 |
| SCML1 | 0,84244679 | 0,00037249 | 0,01340556 | 419,117866 |
| BCAT1 | -4,7029206 | 0,00037195 | 0,01340556 | 53,0180362 |
| GRIK2 | -4,1507149 | 0,00037446 | 0,01342839 | 3,00713259 |
| MGAT2 | -0,7168999 | 0,00037417 | 0,01342839 | 689,278538 |
| ITPRIPL2 | -3,1332873 | 0,00037872 | 0,01355718 | 59,961782 |
| RP11-517C16.2 | -4,505804 | 0,00038103 | 0,01361568 | 4,85862474 |
| MARK2 | -0,9341306 | 0,00038473 | 0,01372357 | 1172,75641 |
| EMB | -0,8808488 | 0,00038662 | 0,01376652 | 1161,46752 |
| MARCH6 | -0,6484561 | 0,00039474 | 0,01388349 | 1027,17393 |
| DTNBP1 | -0,9430134 | 0,00039396 | 0,01388349 | 375,045879 |
| ALDOA | -0,7342325 | 0,00039337 | 0,01388349 | 4420,59809 |
| FAM160A2 | -0,6412637 | 0,00039208 | 0,01388349 | 1345,92423 |
| GJD3 | -4,5088239 | 0,00039241 | 0,01388349 | 4,68606413 |
| SHISA5 | -0,6626193 | 0,00039412 | 0,01388349 | 1133,17504 |
| ZNF592 | -0,574008 | 0,00039253 | 0,01388349 | 3021,88498 |
| CD81 | -0,8443093 | 0,00039605 | 0,01389448 | 1630,51271 |
| ATP5G2 | -0,8394637 | 0,00039643 | 0,01389448 | 1652,71507 |
| IL18R1 | 0,98748997 | 0,00039811 | 0,01392917 | 390,234708 |
| LONRF2 | -4,5013927 | 0,00039989 | 0,01395801 | 19,4483456 |
| CBFA2T3 | -4,7745058 | 0,00040094 | 0,01395801 | 7,41823345 |
| MUL1 | -0,8579694 | 0,00040102 | 0,01395801 | 383,540219 |
| KIAA1328 | 0,82931189 | 0,00040247 | 0,01398438 | 998,824694 |
| ZFP36L1 | -0,7827587 | 0,0004061 | 0,01408606 | 5750,08585 |
| ABHD15 | -0,8394994 | 0,00040684 | 0,01408748 | 418,79576 |
| ECHS1 | -0,8535429 | 0,00040864 | 0,0141051 | 392,591325 |
| CNIH1 | -0,8074473 | 0,00040875 | 0,0141051 | 460,048425 |
| DOCK10 | 0,81270191 | 0,00041173 | 0,01417181 | 457,356389 |
| FYCO1 | 1,05022169 | 0,00041209 | 0,01417181 | 884,740598 |
| CTD-2626G11.2 | -0,9353643 | 0,00041281 | 0,01417227 | 321,093133 |
| MEX3B | -1,9278461 | 0,00041817 | 0,01433168 | 172,053613 |
| CASP1 | 1,58470192 | 0,00042131 | 0,01441491 | 638,568091 |
| INPP5D | 0,59328271 | 0,00042478 | 0,01450899 | 1576,96134 |
| ZG16B | -4,1693057 | 0,00042758 | 0,01455523 | 3,53116367 |
| ATP5A1 | -0,6232198 | 0,00042724 | 0,01455523 | 1121,90715 |
| DAPK3 | -0,9725032 | 0,00042856 | 0,01456379 | 288,578932 |
| PARP1 | -0,7961874 | 0,00043296 | 0,01468862 | 480,480962 |
| ADCK3 | 0,71921075 | 0,00043738 | 0,01481343 | 682,265512 |
| SLC39A1 | -0,9256316 | 0,00044334 | 0,01499023 | 313,296899 |
| RPL37 | 0,61223861 | 0,00044503 | 0,01502197 | 7488,57108 |
| SP110 | 0,70315255 | 0,00046147 | 0,01549897 | 726,644779 |
| RPIA | -1,3534943 | 0,00046072 | 0,01549897 | 471,819427 |
| H2AFZ | -0,8517494 | 0,00046075 | 0,01549897 | 1582,83014 |
| PQLC1 | -0,9841796 | 0,00046624 | 0,01563309 | 312,039585 |
| CTD-2207O23.3 | 0,81750959 | 0,00047098 | 0,01576564 | 547,080726 |
| AC004943.1 | -1,868801 | 0,00047301 | 0,01580725 | 97,4971909 |
| AURKAIP1 | -0,84276 | 0,00047415 | 0,01581902 | 545,032016 |
| TAL1 | -2,3035899 | 0,00048061 | 0,01600804 | 1,11095081 |
| ICAM3 | -0,6675149 | 0,00048359 | 0,0160809 | 2300,31584 |
| EOMES | -6,4450222 | 0,00048911 | 0,01623752 | 13,6084506 |
| RP11-546M21.6 | -3,6488811 | 0,00049049 | 0,01625661 | 2,29315718 |
| FABP5 | -1,513074 | 0,00049382 | 0,01633977 | 256,392736 |
| BOP1 | -1,0653383 | 0,00049546 | 0,01636715 | 246,646529 |
| FAM53C | -1,1498038 | 0,0004978 | 0,01641767 | 252,848596 |
| NCKAP5L | -1,0712968 | 0,00050042 | 0,01647701 | 229,813801 |
| CD83 | 1,07036969 | 0,00050507 | 0,01655864 | 775,489734 |
| SMCHD1 | 0,90832576 | 0,00050559 | 0,01655864 | 839,87155 |
| UBE2J1 | -0,6568047 | 0,00050619 | 0,01655864 | 868,038606 |
| FZR1 | -0,8076419 | 0,00050615 | 0,01655864 | 846,307924 |
| TAOK2 | -0,5928818 | 0,00050827 | 0,01659966 | 1586,18336 |
| MAN1A1 | -0,7099829 | 0,00051099 | 0,01663461 | 656,57811 |
| MYO7A | -6,0375331 | 0,00051039 | 0,01663461 | 356,431563 |
| CD28 | -0,5941211 | 0,00051906 | 0,01686955 | 6118,80343 |
| DUSP5 | -1,6675945 | 0,00051989 | 0,01686955 | 1039,02995 |
| SETDB2 | 0,79265787 | 0,00052828 | 0,01709286 | 604,424512 |
| SH3TC1 | -1,3867144 | 0,00052847 | 0,01709286 | 720,773879 |
| H1F0 | -1,5414329 | 0,00053418 | 0,01724971 | 166,489274 |
| UBE2A | -0,7596813 | 0,00053716 | 0,01731815 | 534,234131 |
| ATPIF1 | -0,5952261 | 0,00053882 | 0,0173441 | 1388,75945 |
| FAM43A | -1,4676824 | 0,00054891 | 0,01764037 | 706,182449 |
| SSBP4 | -1,1594648 | 0,00055677 | 0,01786463 | 447,753989 |
| RP11-862G15.1 | -2,1074403 | 0,00055933 | 0,01791824 | 1,03793793 |
| TOLLIP | -0,9707302 | 0,00056514 | 0,01799427 | 280,955654 |
| AC104024.1 | -3,6574303 | 0,00056429 | 0,01799427 | 2,35946635 |
| WDR86-AS1 | -4,4483289 | 0,00056528 | 0,01799427 | 5,4839923 |
| TESK1 | -1,1589576 | 0,00056426 | 0,01799427 | 274,559633 |
| C11orf96 | -4,7505157 | 0,00056788 | 0,01804837 | 14,9076725 |
| PCSK5 | 2,36689244 | 0,0005817 | 0,01845835 | 128,693952 |
| RP1-37N7.3 | -3,7458058 | 0,00058435 | 0,01851344 | 2,50256517 |
| SPG20 | 2,08726403 | 0,00059133 | 0,01870511 | 82,3650471 |
| CLEC2L | -6,0520981 | 0,00059441 | 0,01877291 | 12,1917367 |
| NLRP2 | 1,21449208 | 0,00059747 | 0,01883994 | 255,079677 |
| SIRPG | -0,618933 | 0,00060063 | 0,01890993 | 1129,47517 |
| CCDC64 | -0,5699963 | 0,00061026 | 0,01918306 | 1799,09949 |
| NEURL1B | -2,3448453 | 0,00063545 | 0,01991281 | 1,12694933 |
| AGPAT1 | -0,9486625 | 0,00063468 | 0,01991281 | 499,336827 |
| MAGED1 | -0,7131079 | 0,00063791 | 0,01995863 | 638,687782 |
| UBE2D3 | -0,62353 | 0,00064215 | 0,0200602 | 2046,76078 |
| ETF1 | -0,66613 | 0,00064374 | 0,02007866 | 787,271599 |
| LTB4R | -1,0172911 | 0,00064555 | 0,02010398 | 280,005333 |
| POLK | 0,86146283 | 0,00065474 | 0,02035857 | 354,191165 |
| CHPF | -2,2365848 | 0,00065917 | 0,02046469 | 220,643341 |
| PDK1 | 1,29426323 | 0,00066112 | 0,0204936 | 155,281957 |
| BMI1 | 1,06483223 | 0,00066454 | 0,02056791 | 223,181033 |
| CABIN1 | -0,7383557 | 0,00067358 | 0,0208159 | 526,063043 |
| YPEL5 | -0,595494 | 0,00067576 | 0,0208512 | 7564,03748 |
| NCR3LG1 | -1,4906518 | 0,00068588 | 0,02113102 | 127,623528 |
| C1orf86 | -0,8529192 | 0,00068716 | 0,02113811 | 701,190125 |
| ZNF609 | -0,8670892 | 0,00069766 | 0,02135612 | 447,938127 |
| TTC37 | 1,0401173 | 0,00069698 | 0,02135612 | 227,951639 |
| ENTPD6 | 0,80335009 | 0,00069667 | 0,02135612 | 414,210039 |
| SYT11 | -0,8476349 | 0,0006985 | 0,02135612 | 938,6369 |
| GAS5 | 0,55251952 | 0,00070663 | 0,02157215 | 2145,18742 |
| PIF1 | -4,6031798 | 0,00071155 | 0,02162372 | 9,13313697 |
| ANKRD13D | -0,7278725 | 0,00070965 | 0,02162372 | 1114,26047 |
| FNDC3A | 0,6294281 | 0,00071073 | 0,02162372 | 1395,60327 |
| KB-1460A1.3 | -3,6236529 | 0,00071813 | 0,02179095 | 2,25612122 |
| SH3BGRL3 | -0,916259 | 0,00072316 | 0,02191047 | 2478,68182 |
| GPR56 | -4,6416406 | 0,00072784 | 0,02201915 | 10,2454136 |
| CNPPD1 | -1,2465304 | 0,00073118 | 0,02208686 | 1118,31382 |
| PFKFB3 | -0,6482585 | 0,00073704 | 0,02223041 | 5582,10376 |
| RAN | -0,6227632 | 0,00074055 | 0,02230297 | 2268,38681 |
| RN7SL811P | -3,9711754 | 0,00075356 | 0,022661 | 2,9583358 |
| RAB27A | -1,1087273 | 0,00075643 | 0,02271309 | 454,152163 |
| MT-TY | 0,99512979 | 0,00076864 | 0,02304551 | 241,302074 |
| NDUFA13 | -0,6476095 | 0,00077184 | 0,02310689 | 1266,97346 |
| CASP8 | 0,76259495 | 0,00077525 | 0,02312294 | 1090,75405 |
| DHX58 | 1,56029536 | 0,00077582 | 0,02312294 | 115,363469 |
| DYRK1B | -0,7072102 | 0,00077441 | 0,02312294 | 690,917978 |
| AC074366.3 | -4,3470611 | 0,00078478 | 0,0233552 | 4,16081292 |
| CCDC101 | 0,87348465 | 0,00078831 | 0,02342559 | 342,21154 |
| TSPO | -1,5188124 | 0,00079618 | 0,02362466 | 344,484633 |
| SUSD4 | 1,59132395 | 0,00080018 | 0,0237084 | 189,717681 |
| RP11-261C10.5 | -4,0044638 | 0,00080883 | 0,02392951 | 2,9871645 |
| PLXDC1 | -1,6264976 | 0,00081124 | 0,02396549 | 180,659268 |
| RP11-65L19.4 | -3,3659265 | 0,00082315 | 0,02424615 | 2,09617839 |
| SNRPB | -0,7734336 | 0,00082276 | 0,02424615 | 1727,32384 |
| RIC8A | -1,1163004 | 0,00082949 | 0,02439711 | 609,697536 |
| AC013474.4 | -4,0586274 | 0,00083251 | 0,02445014 | 3,9237518 |
| INADL | 1,46538969 | 0,00083929 | 0,02461354 | 125,644746 |
| RORC | 1,55418425 | 0,00084897 | 0,02486098 | 121,348645 |
| TNIK | -0,5554715 | 0,0008503 | 0,02486394 | 3362,09603 |
| CNN2 | -1,3443758 | 0,00085528 | 0,02497322 | 3472,01705 |
| MIR146A | 1,44261783 | 0,00085727 | 0,02499513 | 124,402731 |
| EHD4 | -1,1003151 | 0,00086371 | 0,02514643 | 1046,10422 |
| RPS2P5 | -1,5362201 | 0,00087541 | 0,02545023 | 1518,02452 |
| ORAOV1 | 1,00338974 | 0,0008806 | 0,02554847 | 252,841864 |
| AKR1B1 | -1,064379 | 0,00088133 | 0,02554847 | 240,315828 |
| BSG | -1,0618307 | 0,00088609 | 0,02564946 | 994,993593 |
| MME | -4,0360736 | 0,00088959 | 0,02571383 | 4,03998983 |
| SEC61A1 | -0,5863753 | 0,00090204 | 0,02603608 | 1437,6868 |
| RAP2B | -1,5603644 | 0,00091567 | 0,02639078 | 856,412849 |
| ARPC1B | -0,8006997 | 0,00091695 | 0,02639078 | 2623,47912 |
| RP13-192B19.2 | -4,0385639 | 0,00092913 | 0,02670318 | 3,97741717 |
| LIME1 | -0,9221802 | 0,0009488 | 0,02719086 | 765,757006 |
| UBE2R2 | -0,6040224 | 0,0009476 | 0,02719086 | 1023,07625 |
| RING1 | -0,5908503 | 0,00095227 | 0,02719667 | 1098,09736 |
| TRBV5-7 | -4,1750315 | 0,00095403 | 0,02719667 | 4,06073996 |
| CLEC11A | -3,8444067 | 0,00095232 | 0,02719667 | 3,24693949 |
| CRCP | -0,8724308 | 0,00095441 | 0,02719667 | 327,007049 |
| TMEM178B | -4,5287594 | 0,00095727 | 0,02723958 | 10,4423816 |
| ARID5A | -0,6638146 | 0,00095937 | 0,02726079 | 7842,92445 |
| GDF7 | -3,8983752 | 0,00096107 | 0,02727041 | 31,6642866 |
| NDUFS8 | -0,9943239 | 0,00097203 | 0,02750404 | 251,403116 |
| MTA1 | -0,6114535 | 0,00097077 | 0,02750404 | 1153,6567 |
| APOL6 | 0,7581862 | 0,00097602 | 0,02757804 | 2546,24951 |
| S100A6 | 0,63449637 | 0,00098198 | 0,02770742 | 806,038087 |
| RP11-693N9.2 | 1,06770761 | 0,00098795 | 0,02779795 | 414,85002 |
| KIAA0247 | -0,7827276 | 0,00098658 | 0,02779795 | 384,665212 |
| CUTA | -0,5692304 | 0,00099871 | 0,02801306 | 1329,22692 |
| C17orf96 | -1,7216055 | 0,00099951 | 0,02801306 | 240,79138 |
| ZNF668 | -1,3083925 | 0,00099977 | 0,02801306 | 203,033536 |
| MKRN1 | -0,6387296 | 0,00100216 | 0,02804105 | 907,715612 |
| SPATA2 | -0,7205414 | 0,00100813 | 0,02816872 | 595,662714 |
| CTD-2538G9.6 | -3,3824959 | 0,00101061 | 0,02819885 | 2,17809992 |
| KCNQ3 | -4,4381583 | 0,00101611 | 0,0283131 | 7,6729396 |
| ENTPD1 | 1,21022792 | 0,0010194 | 0,0283656 | 573,072854 |
| PSMA7 | -0,7749734 | 0,00102168 | 0,02838959 | 1494,88638 |
| RP11-364C11.3 | -3,6960037 | 0,0010254 | 0,02843071 | 2,75648207 |
| STOM | -2,2460784 | 0,00102598 | 0,02843071 | 299,232561 |
| MIEN1 | -0,99472 | 0,00102758 | 0,02843589 | 235,643025 |
| ATP6AP1 | -0,8015051 | 0,00103143 | 0,02850307 | 370,73901 |
| PSMD9 | -0,7747757 | 0,00103735 | 0,02862758 | 773,550225 |
| PCNA | -0,6198214 | 0,00104439 | 0,02878219 | 864,418824 |
| CXorf57 | -1,2169367 | 0,00105799 | 0,0291171 | 175,65872 |
| TIPARP | -0,7423246 | 0,00106067 | 0,02915095 | 1484,05665 |
| RN7SL522P | -3,6375072 | 0,0010679 | 0,02930969 | 2,46021616 |
| XXYLT1 | -1,8566362 | 0,00107499 | 0,02946422 | 135,601878 |
| SELT | -0,7009073 | 0,00108392 | 0,02966839 | 555,776042 |
| NCOA1 | -0,5612689 | 0,00109214 | 0,02981364 | 2556,13378 |
| COBLL1 | -4,091861 | 0,00109219 | 0,02981364 | 4,52072032 |
| TIMD4 | 2,99797622 | 0,00110566 | 0,03014045 | 42,6000669 |
| NUDT16L1 | -0,7853695 | 0,00110963 | 0,03020782 | 609,008887 |
| CD6 | -0,8230176 | 0,00111125 | 0,0302111 | 5079,73688 |
| HDGFRP3 | -3,5252199 | 0,00112373 | 0,03050913 | 2,58212935 |
| RP11-474B16.1 | -3,2560311 | 0,00112882 | 0,03060587 | 2,01348324 |
| BCAP31 | -1,0235717 | 0,00113472 | 0,03072452 | 558,25916 |
| FAM78B | -4,2018626 | 0,00114076 | 0,03084662 | 21,1659863 |
| JOSD1 | -0,6812276 | 0,00114893 | 0,03100677 | 704,134763 |
| ATP5D | -0,6837858 | 0,00114977 | 0,03100677 | 814,209367 |
| CD2BP2 | -0,6397176 | 0,00115664 | 0,0311502 | 774,999458 |
| AC110771.1 | -4,0870048 | 0,00117001 | 0,03146828 | 3,77234864 |
| RYK | 0,91001463 | 0,00118749 | 0,0318116 | 276,943097 |
| RP11-536K7.5 | 2,93091323 | 0,00119068 | 0,0318116 | 43,7975911 |
| EDF1 | -0,5245035 | 0,00118463 | 0,0318116 | 2513,71885 |
| PDE4D | -1,8625808 | 0,00119052 | 0,0318116 | 607,123697 |
| PPP3CA | 1,13390614 | 0,00119066 | 0,0318116 | 188,100135 |
| IL1R2 | 3,60024245 | 0,00119593 | 0,03186693 | 92,5211401 |
| MT1JP | -3,6623912 | 0,00119909 | 0,03186693 | 3,17824848 |
| ATP9A | -5,169729 | 0,00119859 | 0,03186693 | 58,2229009 |
| SUFU | -1,0207906 | 0,00119512 | 0,03186693 | 217,806944 |
| RP5-1063M23.1 | -3,6784072 | 0,0012007 | 0,03186777 | 2,90099736 |
| AC005264.2 | -3,7032785 | 0,00120595 | 0,03196485 | 3,04663397 |
| SNX17 | -0,7024767 | 0,00121422 | 0,03214159 | 1263,55319 |
| HNRNPF | -0,5528763 | 0,00123817 | 0,03273263 | 3956,92379 |
| NFE2L3 | 1,69912416 | 0,00124366 | 0,03283465 | 218,484659 |
| RP11-330A16.1 | -4,308283 | 0,00124809 | 0,03290827 | 12,8688242 |
| ZBTB47 | -4,4100009 | 0,00125852 | 0,03313991 | 7,69974902 |
| TMBIM6 | -0,5349979 | 0,00126406 | 0,03324223 | 1747,32029 |
| PRDX5 | -0,8504763 | 0,00126618 | 0,03325463 | 397,398333 |
| MDH2 | -0,698744 | 0,00127298 | 0,03338862 | 541,608021 |
| TPI1 | -0,7526097 | 0,0012746 | 0,03338862 | 1303,5682 |
| SYTL3 | 0,87502931 | 0,00128899 | 0,03372155 | 1033,16935 |
| CEP120 | 0,57733121 | 0,00129875 | 0,03389637 | 1457,74344 |
| GNB2 | -0,8806981 | 0,00129904 | 0,03389637 | 1963,25319 |
| FANK1 | 2,71488527 | 0,00130259 | 0,03394343 | 49,2741247 |
| KRTAP16-1 | -3,9017846 | 0,00130422 | 0,03394343 | 3,94093596 |
| RP11-169E6.1 | -3,7893834 | 0,00131963 | 0,03430023 | 3,53703064 |
| VPS54 | 0,64409488 | 0,00132316 | 0,03434745 | 716,057727 |
| AC109925.1 | -3,5903043 | 0,00133236 | 0,03454178 | 2,69961933 |
| ADAMTS7P2 | -3,3661062 | 0,00134327 | 0,03468699 | 2,3305849 |
| UBL5 | -0,615872 | 0,00134201 | 0,03468699 | 816,244945 |
| SPPL2B | -1,0167156 | 0,00134239 | 0,03468699 | 1019,96668 |
| GNAT2 | -3,5040895 | 0,00134658 | 0,03468699 | 2,65600076 |
| LGR6 | -4,325812 | 0,00134534 | 0,03468699 | 8,58917578 |
| SLC4A5 | 1,15157519 | 0,00136489 | 0,03511359 | 219,382561 |
| GATA3 | -1,2379587 | 0,00137099 | 0,03522543 | 1842,52564 |
| H1FX | -0,8962983 | 0,00137427 | 0,03526478 | 3121,5787 |
| HSPA6 | -3,7073013 | 0,00137752 | 0,03530311 | 3,21692122 |
| MERTK | -4,7807338 | 0,00138984 | 0,03551938 | 43,6992897 |
| SLC10A3 | -1,1124398 | 0,00139126 | 0,03551938 | 508,573129 |
| MDK | -4,4702537 | 0,00138821 | 0,03551938 | 6,38440313 |
| GADD45A | -1,4071659 | 0,00139729 | 0,03562825 | 787,75906 |
| YWHAQ | -0,6181479 | 0,00139948 | 0,03563902 | 775,289176 |
| NAA60 | 1,86919117 | 0,0014042 | 0,03571385 | 120,21393 |
| KCNG1 | -4,1539125 | 0,00141468 | 0,03589506 | 5,22056585 |
| QSOX2 | -0,9756261 | 0,00141489 | 0,03589506 | 1473,12895 |
| GPSM3 | -0,9134984 | 0,00142712 | 0,03615961 | 2483,42901 |
| MAPRE1 | -0,6496133 | 0,00142906 | 0,03616327 | 2194,03188 |
| HPGDS | -3,976374 | 0,00143161 | 0,03618231 | 4,5349751 |
| NFIA | -4,6118614 | 0,00143519 | 0,03619964 | 26,7587384 |
| SLC25A39 | -0,6419058 | 0,00143589 | 0,03619964 | 691,611816 |
| RNF11 | -0,717613 | 0,00144744 | 0,03639945 | 495,696764 |
| CD5 | -0,8697421 | 0,00144575 | 0,03639945 | 7074,14813 |
| RP11-87G24.6 | -3,9532004 | 0,00145833 | 0,03662749 | 4,3312425 |
| LRRC24 | -4,2702915 | 0,00146905 | 0,03685085 | 5,93961453 |
| SNORD62A | -3,8573765 | 0,00147873 | 0,0369519 | 4,06892719 |
| SNORD62B | -3,8573765 | 0,00147873 | 0,0369519 | 4,06892719 |
| RP4-761J14.8 | -4,1379731 | 0,00148043 | 0,0369519 | 3,39971082 |
| HIATL1 | -0,6589938 | 0,00147954 | 0,0369519 | 653,146037 |
| SH3GLB1 | -0,8953778 | 0,00148317 | 0,03697458 | 544,906074 |
| HMGN4 | -0,7809993 | 0,00148584 | 0,03699528 | 390,884878 |
| HIGD2A | -0,6599235 | 0,00149012 | 0,03705602 | 625,275604 |
| CSTB | -0,8985241 | 0,00149598 | 0,03715563 | 535,995619 |
| AC092798.2 | -3,9198809 | 0,00151796 | 0,03760882 | 4,35875165 |
| ZMAT3 | -1,4345792 | 0,00151679 | 0,03760882 | 324,694764 |
| MS4A6A | -4,1355194 | 0,00152277 | 0,03768148 | 17,0703697 |
| MOB4 | -0,8762192 | 0,00152657 | 0,03772913 | 292,700367 |
| GTPBP1 | -0,9172268 | 0,00153109 | 0,03779445 | 671,96034 |
| NDUFS7 | -1,0851249 | 0,00153821 | 0,03792378 | 675,04839 |
| FMR1-AS1 | -3,2151779 | 0,0015489 | 0,03814059 | 2,19491764 |
| EP400NL | -1,2434042 | 0,00158516 | 0,03891828 | 220,842938 |
| USF2 | -0,5136824 | 0,00158407 | 0,03891828 | 2819,33029 |
| CLASP2 | 0,89961675 | 0,00158629 | 0,03891828 | 277,050423 |
| FAM107B | -0,5358185 | 0,00159957 | 0,03919627 | 1483,31842 |
| ZNF512B | -0,6842109 | 0,00160534 | 0,039242 | 1020,79202 |
| SLC30A9 | 0,95973325 | 0,00160404 | 0,039242 | 232,935338 |
| C20orf24 | -0,6729897 | 0,00160782 | 0,03925506 | 571,103889 |
| FAM168B | -0,5248417 | 0,00161756 | 0,03944485 | 2138,13189 |
| ODF2L | 0,76359923 | 0,00163848 | 0,03986674 | 391,049664 |
| SPATA2L | -1,0649097 | 0,00163882 | 0,03986674 | 186,729558 |
| RP5-1028K7.2 | -4,9077875 | 0,00164408 | 0,03994634 | 87,7047526 |
| TRGC2 | -5,0078043 | 0,00167068 | 0,04050884 | 21,9743579 |
| AGPAT2 | -1,1114586 | 0,00167126 | 0,04050884 | 246,20376 |
| RAB35 | -0,5799375 | 0,00167589 | 0,04053355 | 1053,83181 |
| AC091633.3 | -4,2723333 | 0,00167748 | 0,04053355 | 4,0909715 |
| WIZ | -0,6606343 | 0,00167832 | 0,04053355 | 671,097527 |
| ADAMTS7P4 | -4,3214027 | 0,00168858 | 0,04073241 | 8,05779845 |
| TRBV3-1 | -0,9129421 | 0,00170834 | 0,04115965 | 255,422465 |
| RN7SKP70 | -3,7284122 | 0,00172391 | 0,04146681 | 3,4779739 |
| ASNA1 | -0,917869 | 0,00172521 | 0,04146681 | 423,51977 |
| MYO1E | -4,24242 | 0,00173854 | 0,04173741 | 8,6324388 |
| FSIP2 | 1,08781309 | 0,00174915 | 0,04194217 | 184,877371 |
| CTD-2562J15.4 | -3,8633896 | 0,00175611 | 0,04202786 | 4,19931317 |
| CMTM1 | -4,1996656 | 0,00175691 | 0,04202786 | 6,83018502 |
| AC020910.2 | -3,9505229 | 0,00175997 | 0,0420512 | 4,85421542 |
| RP13-93L13.1 | -3,6449577 | 0,00176922 | 0,04222196 | 3,3643454 |
| NDUFB2 | -0,8135906 | 0,00177446 | 0,04225966 | 560,709991 |
| TMEM200A | -4,7303814 | 0,001775 | 0,04225966 | 27,8074122 |
| LINC00865 | -4,0288165 | 0,00178443 | 0,04243392 | 5,36997947 |
| MT-TE | 1,2794992 | 0,00178903 | 0,04243497 | 136,206225 |
| MMP17 | -4,2097541 | 0,00179243 | 0,04243497 | 8,23651013 |
| MRPL14 | -1,1509589 | 0,00179198 | 0,04243497 | 168,72673 |
| DCAF13 | -0,9615561 | 0,00179291 | 0,04243497 | 242,543627 |
| LINC00862 | -3,0780902 | 0,00180365 | 0,04263907 | 1,94548331 |
| KIAA0319L | -0,6511955 | 0,00180673 | 0,04266181 | 662,8551 |
| AC144449.1 | -4,2693244 | 0,00181393 | 0,04276297 | 5,6214495 |
| ZNF292 | 0,80924038 | 0,00181527 | 0,04276297 | 322,096511 |
| PMCHL2 | -3,0388593 | 0,00182753 | 0,04295126 | 1,97773306 |
| RP11-73M14.1 | -3,6723122 | 0,00182699 | 0,04295126 | 3,50327934 |
| CACNA1I | -0,9323239 | 0,00184229 | 0,04319715 | 253,337877 |
| ZC3H7A | 0,59547705 | 0,0018413 | 0,04319715 | 852,425447 |
| RP11-431M7.3 | -4,0580875 | 0,00185434 | 0,04342908 | 5,14301955 |
| AC090505.4 | -3,6295811 | 0,00186329 | 0,04358797 | 2,99664664 |
| LPAL2 | 1,82216819 | 0,00186981 | 0,04363906 | 166,735353 |
| TRPS1 | -0,7391605 | 0,00186884 | 0,04363906 | 435,836072 |
| KLF8 | 1,94051254 | 0,00187955 | 0,04381165 | 101,728362 |
| DYNLRB1 | -0,5698155 | 0,00188156 | 0,04381165 | 1078,24582 |
| ACADVL | -0,5123823 | 0,00188685 | 0,04388388 | 2017,64913 |
| SYCP1 | -4,1505734 | 0,0018958 | 0,04404114 | 5,74345071 |
| SIPA1L2 | -2,2458103 | 0,00190781 | 0,04426919 | 77,5665578 |
| GUK1 | -0,6872099 | 0,00192101 | 0,04446028 | 2300,90394 |
| STK11IP | -0,9035958 | 0,00191961 | 0,04446028 | 278,582683 |
| CNTRL | 0,52255827 | 0,00192268 | 0,04446028 | 1884,99076 |
| IRF2BPL | -0,5378815 | 0,00193105 | 0,04451539 | 1438,32716 |
| CCL3 | -4,255304 | 0,00193161 | 0,04451539 | 4,51872196 |
| PSMD8 | -0,7108384 | 0,0019317 | 0,04451539 | 956,878035 |
| RP3-339A18.6 | -3,776322 | 0,0019617 | 0,04515499 | 3,63271638 |
| ARAP3 | 2,62672062 | 0,0019652 | 0,04518396 | 77,5741057 |
| CD226 | -1,019149 | 0,00197769 | 0,04541909 | 259,275821 |
| PPP6R1 | -0,5324698 | 0,00199773 | 0,04582714 | 1383,08349 |
| RP11-353K11.1 | -3,5467704 | 0,00201008 | 0,0460578 | 3,17131624 |
| DMC1 | -3,4904552 | 0,00201274 | 0,04606639 | 2,89822238 |
| AC012065.7 | -4,2209113 | 0,00202322 | 0,0462536 | 11,3359331 |
| SRRT | -0,5515799 | 0,00202935 | 0,04634115 | 1176,36785 |
| RNASET2 | -0,52152 | 0,00203299 | 0,04637154 | 3993,4846 |
| TLR5 | 1,62596996 | 0,00203608 | 0,04638933 | 103,420551 |
| ANXA7 | -0,5656946 | 0,00206566 | 0,04697272 | 996,688749 |
| PSMA3 | 0,64474972 | 0,00206635 | 0,04697272 | 585,809459 |
| GRAMD4 | -1,284194 | 0,00206929 | 0,04698642 | 383,064305 |
| CAMK2N2 | -4,6826728 | 0,00207454 | 0,04705258 | 4,91710869 |
| AC018445.1 | -3,7643049 | 0,00209485 | 0,04745979 | 4,11965621 |
| BRWD1 | 0,646018 | 0,00210462 | 0,04762745 | 612,801987 |
| ACAP3 | -0,7871651 | 0,00210793 | 0,04764862 | 369,064474 |
| CCDC88B | -0,5024561 | 0,00212916 | 0,04802074 | 4327,51096 |
| KHSRP | -0,5679676 | 0,00212873 | 0,04802074 | 1470,44079 |
| IER5L | -1,4406251 | 0,002135 | 0,04809849 | 248,027297 |
| TNFRSF13C | 1,80601937 | 0,00213978 | 0,04815223 | 85,9088276 |
| PDE4DIP | 0,62800882 | 0,00214917 | 0,04830944 | 642,878893 |
| GNAS | -0,5285966 | 0,00215416 | 0,04836754 | 6918,76188 |
| SCAND1 | -0,7388584 | 0,00216397 | 0,04853381 | 1007,52694 |
| RP11-932O9.9 | -4,4077363 | 0,00216925 | 0,048598 | 5,80963748 |
| JMJD6 | -0,5822733 | 0,00217598 | 0,04869455 | 1010,83291 |
| C8orf82 | -1,1359059 | 0,00218055 | 0,04874259 | 168,672776 |
| ZNF780A | 0,75534194 | 0,00218921 | 0,04888173 | 448,44835 |
| EMC10 | -0,5571665 | 0,00219629 | 0,04898563 | 2220,18071 |
| SPINT2 | -0,8538126 | 0,00220401 | 0,04910338 | 272,818964 |
| MTHFD2 | -0,6775978 | 0,00222645 | 0,04954838 | 510,387582 |
