# Supplementary material for: The impact of HIV infection on the frequencies, function, spatial localization and heterogeneity of T follicular regulatory cells (TFRs) within human lymph nodes
Source: BMC Immunol. 2022 Jul 1;23:34. doi: 10.1186/s12865-022-00508-1 (PMC9250173; doi:10.1186/s12865-022-00508-1)
Supplement: Supplementary file 7 — Additional file7. List of differentially accessible genes. [file 12865_2022_508_MOESM7_ESM.docx]

**Additional file 7. List of differentially accessible genes.**

| **log2 change in expression** | **ext_gene** | **Concordance between differential expression and accessibility** | **Proportion of concordant peaks** |
| --- | --- | --- | --- |
| 2,191478691 | GBP5 | No | 0,25 |
| 2,191478691 | GBP5 | Yes | 0,75 |
| -1,720993329 | ITM2A | Yes | 0,818181818 |
| -1,720993329 | ITM2A | No | 0,181818182 |
| 1,804360844 | FCRL3 | Yes | 1 |
| -1,569621482 | TOX | Yes | 0,731343284 |
| -1,569621482 | TOX | No | 0,268656716 |
| -1,930776509 | KCNK5 | Yes | 1 |
| -2,836832927 | IGFBP4 | Yes | 1 |
| -1,467011197 | NFATC2 | Yes | 0,882352941 |
| -1,467011197 | NFATC2 | No | 0,117647059 |
| 2,498521543 | IL2RA | Yes | 1 |
| -2,534648576 | TOX2 | No | 0,1 |
| -2,534648576 | TOX2 | Yes | 0,9 |
| 1,794158593 | HAPLN3 | Yes | 1 |
| -1,77551892 | CXCR5 | Yes | 0,8 |
| -1,77551892 | CXCR5 | No | 0,2 |
| -1,741214719 | NCDN | Yes | 0,846153846 |
| -1,741214719 | NCDN | No | 0,153846154 |
| 2,989211834 | TTN | No | 0,6 |
| 2,989211834 | TTN | Yes | 0,4 |
| -3,810158017 | PDCD1 | Yes | 1 |
| -2,281027627 | COTL1 | Yes | 0,857142857 |
| -2,281027627 | COTL1 | No | 0,142857143 |
| -1,238484408 | ICOS | No | 0,189189189 |
| -1,238484408 | ICOS | Yes | 0,810810811 |
| -1,547682886 | RHOB | Yes | 1 |
| 1,726317291 | TMEM63A | Yes | 0,75 |
| 1,726317291 | TMEM63A | No | 0,25 |
| -1,348889247 | RNF19A | Yes | 0,9 |
| -1,348889247 | RNF19A | No | 0,1 |
| 3,197952034 | SELP | Yes | 1 |
| -2,193726738 | TNFSF8 | Yes | 0,777777778 |
| -2,193726738 | TNFSF8 | No | 0,222222222 |
| -1,648111354 | ST8SIA1 | Yes | 0,636363636 |
| -1,648111354 | ST8SIA1 | No | 0,363636364 |
| -1,482165544 | CD82 | No | 0,272727273 |
| -1,482165544 | CD82 | Yes | 0,727272727 |
| 1,81152896 | TXK | No | 0,333333333 |
| 1,81152896 | TXK | Yes | 0,666666667 |
| -1,544230859 | POMT1 | Yes | 0,666666667 |
| -1,544230859 | POMT1 | No | 0,333333333 |
| -1,730524961 | TCF7 | Yes | 0,857142857 |
| -1,730524961 | TCF7 | No | 0,142857143 |
| 2,042664492 | ADTRP | No | 0,714285714 |
| 2,042664492 | ADTRP | Yes | 0,285714286 |
| 1,856254725 | FGF23 | Yes | 1 |
| 1,66391387 | PARP15 | Yes | 1 |
| -1,659903159 | GSE1 | Yes | 0,7 |
| -1,659903159 | GSE1 | No | 0,3 |
| 2,238187953 | NR3C2 | Yes | 0,857142857 |
| 2,238187953 | NR3C2 | No | 0,142857143 |
| -1,13541576 | ITPKB | Yes | 0,766666667 |
| -1,13541576 | ITPKB | No | 0,233333333 |
| 1,331062649 | MAP3K1 | No | 0,238095238 |
| 1,331062649 | MAP3K1 | Yes | 0,761904762 |
| -1,307916545 | C16orf54 | No | 0,25 |
| -1,307916545 | C16orf54 | Yes | 0,75 |
| 1,784920041 | PLCL1 | Yes | 0,928571429 |
| 1,784920041 | PLCL1 | No | 0,071428571 |
| 2,574267263 | FOXP3 | Yes | 1 |
| 1,571083302 | SLC12A6 | Yes | 1 |
| 2,673892875 | RTKN2 | Yes | 0,555555556 |
| 2,673892875 | RTKN2 | No | 0,444444444 |
| 2,767974988 | ATP1B1 | Yes | 0,75 |
| 2,767974988 | ATP1B1 | No | 0,25 |
| 1,680415338 | EGR1 | No | 0,4 |
| 1,680415338 | EGR1 | Yes | 0,6 |
| 1,273086859 | AHNAK | No | 0,444444444 |
| 1,273086859 | AHNAK | Yes | 0,555555556 |
| 3,498735005 | IL1R1 | Yes | 0,6 |
| 3,498735005 | IL1R1 | No | 0,4 |
| -1,805862949 | PLXND1 | Yes | 0,8 |
| -1,805862949 | PLXND1 | No | 0,2 |
| -1,688473919 | SH2D1A | Yes | 0,939393939 |
| -1,688473919 | SH2D1A | No | 0,060606061 |
| 1,576037366 | F5 | Yes | 1 |
| -1,614586242 | TRAT1 | Yes | 0,8 |
| -1,614586242 | TRAT1 | No | 0,2 |
| -1,412957696 | NFATC1 | No | 0,137931034 |
| -1,412957696 | NFATC1 | Yes | 0,862068966 |
| -1,044709727 | PPP1CC | Yes | 0,714285714 |
| -1,044709727 | PPP1CC | No | 0,285714286 |
| 1,517407165 | SAMHD1 | Yes | 1 |
| 1,137839376 | RASGRP2 | Yes | 1 |
| -1,8534858 | FKBP5 | Yes | 0,666666667 |
| -1,8534858 | FKBP5 | No | 0,333333333 |
| 1,957582583 | IKZF2 | Yes | 0,882352941 |
| 1,957582583 | IKZF2 | No | 0,117647059 |
| -1,365471968 | MXD4 | No | 0,666666667 |
| -1,365471968 | MXD4 | Yes | 0,333333333 |
| 2,190091488 | SPON1 | Yes | 0,727272727 |
| 2,190091488 | SPON1 | No | 0,272727273 |
| 1,659167619 | TRPM2 | Yes | 1 |
| 1,687261091 | FLT3LG | Yes | 1 |
| -2,617510074 | SMCO4 | Yes | 1 |
| 1,40337131 | MYO15B | Yes | 1 |
| 1,079822073 | SLFN5 | Yes | 0,75 |
| 1,079822073 | SLFN5 | No | 0,25 |
| 1,809974623 | KIAA0040 | Yes | 0,727272727 |
| 1,809974623 | KIAA0040 | No | 0,272727273 |
| 1,216666494 | BIRC3 | No | 0,25 |
| 1,216666494 | BIRC3 | Yes | 0,75 |
| -7,230956062 | GNG4 | Yes | 1 |
| -1,035462897 | TC2N | No | 0,6 |
| -1,035462897 | TC2N | Yes | 0,4 |
| 1,047448352 | CAST | Yes | 0,8 |
| 1,047448352 | CAST | No | 0,2 |
| -1,086033878 | NIN | Yes | 0,714285714 |
| -1,086033878 | NIN | No | 0,285714286 |
| -1,480410417 | FBXO32 | Yes | 0,888888889 |
| -1,480410417 | FBXO32 | No | 0,111111111 |
| 1,516871846 | PDE8A | Yes | 1 |
| -8,653863201 | CXCL13 | No | 0,181818182 |
| -8,653863201 | CXCL13 | Yes | 0,818181818 |
| -1,300100541 | SLC25A46 | No | 0,333333333 |
| -1,300100541 | SLC25A46 | Yes | 0,666666667 |
| -1,572456557 | MT1E | Yes | 1 |
| -0,871465384 | MCL1 | Yes | 0,666666667 |
| -0,871465384 | MCL1 | No | 0,333333333 |
| 1,03323438 | GOLGA8A | Yes | 1 |
| -2,045125665 | TENM1 | Yes | 1 |
| 1,101052202 | OXNAD1 | Yes | 0,764705882 |
| 1,101052202 | OXNAD1 | No | 0,235294118 |
| 1,669040227 | THEM4 | Yes | 0,8 |
| 1,669040227 | THEM4 | No | 0,2 |
| 1,424725536 | SGTB | Yes | 1 |
| -0,892897131 | ITGB2 | No | 0,6 |
| -0,892897131 | ITGB2 | Yes | 0,4 |
| -0,945813254 | TRERF1 | No | 0,133333333 |
| -0,945813254 | TRERF1 | Yes | 0,866666667 |
| -1,627561043 | TRIM8 | Yes | 0,75 |
| -1,627561043 | TRIM8 | No | 0,25 |
| 1,171353066 | VPS13C | Yes | 1 |
| 1,460424406 | RORA | No | 0,25 |
| 1,460424406 | RORA | Yes | 0,75 |
| -1,302165251 | MAP3K8 | No | 0,6 |
| -1,302165251 | MAP3K8 | Yes | 0,4 |
| 1,018184354 | GOLGA8B | Yes | 1 |
| 0,98330216 | HS3ST3B1 | Yes | 1 |
| -0,891177795 | HNRNPLL | Yes | 0,80952381 |
| -0,891177795 | HNRNPLL | No | 0,19047619 |
| -7,035411711 | NTRK3 | Yes | 1 |
| -1,209005272 | ANKRD13A | No | 0,333333333 |
| -1,209005272 | ANKRD13A | Yes | 0,666666667 |
| -2,935631707 | F2R | Yes | 0,833333333 |
| -2,935631707 | F2R | No | 0,166666667 |
| -1,045490448 | STX11 | Yes | 0,846153846 |
| -1,045490448 | STX11 | No | 0,153846154 |
| -2,380965873 | THEMIS | Yes | 0,634146341 |
| -2,380965873 | THEMIS | No | 0,365853659 |
| -3,966520905 | CNKSR3 | Yes | 0,666666667 |
| -3,966520905 | CNKSR3 | No | 0,333333333 |
| -2,810739148 | DUSP6 | Yes | 0,8 |
| -2,810739148 | DUSP6 | No | 0,2 |
| -1,984255809 | KIAA1671 | Yes | 0,8 |
| -1,984255809 | KIAA1671 | No | 0,2 |
| -6,22576693 | SHISA2 | Yes | 0,909090909 |
| -6,22576693 | SHISA2 | No | 0,090909091 |
| 1,605189125 | CARD16 | Yes | 1 |
| -1,097290111 | ZBTB10 | Yes | 0,7 |
| -1,097290111 | ZBTB10 | No | 0,3 |
| 1,192033253 | DGKH | Yes | 0,5 |
| 1,192033253 | DGKH | No | 0,5 |
| 1,652226241 | ZNF652 | Yes | 1 |
| -1,255528909 | NR3C1 | Yes | 0,92 |
| -1,255528909 | NR3C1 | No | 0,08 |
| 1,202429645 | MLKL | Yes | 1 |
| 2,278332129 | TLDC2 | Yes | 1 |
| -0,841970077 | GNA13 | No | 0,714285714 |
| -0,841970077 | GNA13 | Yes | 0,285714286 |
| -0,943046998 | ICA1 | No | 0,318181818 |
| -0,943046998 | ICA1 | Yes | 0,681818182 |
| 1,32306483 | RGS1 | Yes | 0,653846154 |
| 1,32306483 | RGS1 | No | 0,346153846 |
| -0,84434665 | SRGN | Yes | 0,692307692 |
| -0,84434665 | SRGN | No | 0,307692308 |
| -1,279060447 | THADA | Yes | 0,8 |
| -1,279060447 | THADA | No | 0,2 |
| 1,246040757 | CASK | Yes | 0,714285714 |
| 1,246040757 | CASK | No | 0,285714286 |
| -1,122250206 | PTPRCAP | Yes | 0,333333333 |
| -1,122250206 | PTPRCAP | No | 0,666666667 |
| -0,806727402 | SH3KBP1 | Yes | 0,740740741 |
| -0,806727402 | SH3KBP1 | No | 0,259259259 |
| 1,086564449 | ITGB7 | No | 0,333333333 |
| 1,086564449 | ITGB7 | Yes | 0,666666667 |
| 0,891593036 | RPL9 | Yes | 1 |
| -6,08922985 | TNFSF9 | No | 0,375 |
| -6,08922985 | TNFSF9 | Yes | 0,625 |
| -6,030393284 | FCRL6 | Yes | 1 |
| 1,107223929 | KLHL2 | Yes | 1 |
| -4,760898421 | CRTAM | Yes | 0,866666667 |
| -4,760898421 | CRTAM | No | 0,133333333 |
| 2,19385853 | LAYN | Yes | 1 |
| 0,759497659 | TMSB10 | No | 0,428571429 |
| 0,759497659 | TMSB10 | Yes | 0,571428571 |
| -0,81612278 | IKZF3 | Yes | 0,833333333 |
| -0,81612278 | IKZF3 | No | 0,166666667 |
| 1,562744533 | CD55 | Yes | 1 |
| -5,800393825 | GZMH | No | 0,5 |
| -5,800393825 | GZMH | Yes | 0,5 |
| 1,203502896 | ZNF274 | Yes | 1 |
| -5,9054301 | PVALB | Yes | 1 |
| -0,990295747 | LRMP | No | 0,2 |
| -0,990295747 | LRMP | Yes | 0,8 |
| 1,130041519 | MLLT3 | No | 0,333333333 |
| 1,130041519 | MLLT3 | Yes | 0,666666667 |
| -0,743447306 | SIK1 | No | 0,5 |
| -0,743447306 | SIK1 | Yes | 0,5 |
| 1,540785127 | MIAT | No | 0,222222222 |
| 1,540785127 | MIAT | Yes | 0,777777778 |
| 1,170719339 | ALMS1 | Yes | 1 |
| -5,666778071 | FZD7 | Yes | 0,5 |
| -5,666778071 | FZD7 | No | 0,5 |
| -1,199541161 | KIAA1324 | Yes | 1 |
| 1,208470888 | CCDC66 | Yes | 1 |
| 1,178510968 | VAV3 | Yes | 0,428571429 |
| 1,178510968 | VAV3 | No | 0,571428571 |
| -0,798972194 | ITGAL | No | 0,666666667 |
| -0,798972194 | ITGAL | Yes | 0,333333333 |
| 1,361374583 | ITGA6 | Yes | 1 |
| 2,017610653 | CSF2RB | Yes | 1 |
| 1,02898402 | IL10RA | No | 0,285714286 |
| 1,02898402 | IL10RA | Yes | 0,714285714 |
| 2,25581249 | PCDH1 | Yes | 1 |
| -2,117365817 | MAP7 | Yes | 0,666666667 |
| -2,117365817 | MAP7 | No | 0,333333333 |
| -1,457395175 | NR4A3 | No | 0,777777778 |
| -1,457395175 | NR4A3 | Yes | 0,222222222 |
| -0,986507504 | GALNT2 | Yes | 0,875 |
| -0,986507504 | GALNT2 | No | 0,125 |
| -0,795367101 | CAPZB | No | 0,571428571 |
| -0,795367101 | CAPZB | Yes | 0,428571429 |
| -0,866826552 | IRF2BP2 | No | 0,6 |
| -0,866826552 | IRF2BP2 | Yes | 0,4 |
| -5,02578321 | MYO6 | Yes | 1 |
| -0,887969632 | SLC9A3R1 | Yes | 0,4 |
| -0,887969632 | SLC9A3R1 | No | 0,6 |
| -5,946272652 | PEG10 | No | 0,333333333 |
| -5,946272652 | PEG10 | Yes | 0,666666667 |
| -1,08697009 | DHRS7 | Yes | 0,777777778 |
| -1,08697009 | DHRS7 | No | 0,222222222 |
| -1,79640207 | CD40LG | Yes | 1 |
| 1,708443777 | CISH | Yes | 1 |
| -3,765949382 | FGFR2 | Yes | 1 |
| -5,859744848 | CLCN4 | Yes | 0,875 |
| -5,859744848 | CLCN4 | No | 0,125 |
| -1,157702884 | MID1IP1 | Yes | 0,666666667 |
| -1,157702884 | MID1IP1 | No | 0,333333333 |
| 4,404358299 | SOCS2 | Yes | 1 |
| 1,143526634 | FAM184A | Yes | 0,333333333 |
| 1,143526634 | FAM184A | No | 0,666666667 |
| -0,837256423 | IDH2 | No | 0,454545455 |
| -0,837256423 | IDH2 | Yes | 0,545454545 |
| -1,472016342 | GZMM | Yes | 0,666666667 |
| -1,472016342 | GZMM | No | 0,333333333 |
| 1,841726654 | SETD7 | No | 0,25 |
| 1,841726654 | SETD7 | Yes | 0,75 |
| -1,130790213 | GIMAP6 | Yes | 1 |
| -5,707186398 | CAV1 | Yes | 0,8 |
| -5,707186398 | CAV1 | No | 0,2 |
| 6,940119036 | UQCR11 | Yes | 1 |
| -1,448751611 | ARAP2 | Yes | 0,789473684 |
| -1,448751611 | ARAP2 | No | 0,210526316 |
| -1,876283461 | POU2AF1 | Yes | 1 |
| -0,717780322 | ZNF331 | No | 0,5 |
| -0,717780322 | ZNF331 | Yes | 0,5 |
| -5,67506927 | INHBB | No | 0,166666667 |
| -5,67506927 | INHBB | Yes | 0,833333333 |
| -0,961359741 | PTPN11 | No | 0,142857143 |
| -0,961359741 | PTPN11 | Yes | 0,857142857 |
| 1,047740455 | STAM | Yes | 0,818181818 |
| 1,047740455 | STAM | No | 0,181818182 |
| -0,932329992 | CD84 | Yes | 0,818181818 |
| -0,932329992 | CD84 | No | 0,181818182 |
| -1,179092141 | ZNF518B | No | 0,5 |
| -1,179092141 | ZNF518B | Yes | 0,5 |
| 1,232460222 | RASSF3 | Yes | 1 |
| -5,383200263 | IL21 | Yes | 0,941176471 |
| -5,383200263 | IL21 | No | 0,058823529 |
| -0,753871524 | GYPC | Yes | 0,272727273 |
| -0,753871524 | GYPC | No | 0,727272727 |
| -0,877366669 | TOB2 | No | 0,75 |
| -0,877366669 | TOB2 | Yes | 0,25 |
| -1,274141467 | INSIG1 | No | 0,625 |
| -1,274141467 | INSIG1 | Yes | 0,375 |
| 1,166496113 | LRRC37B | Yes | 1 |
| -0,758722389 | GAPDH | No | 0,6 |
| -0,758722389 | GAPDH | Yes | 0,4 |
| -5,374463872 | ASCL2 | Yes | 1 |
| -1,042986269 | SEMA4D | Yes | 0,666666667 |
| -1,042986269 | SEMA4D | No | 0,333333333 |
| -0,740825054 | SIK3 | No | 0,1 |
| -0,740825054 | SIK3 | Yes | 0,9 |
| 0,972156192 | ERAP2 | Yes | 0,714285714 |
| 0,972156192 | ERAP2 | No | 0,285714286 |
| 1,52642457 | KLF11 | Yes | 0,6 |
| 1,52642457 | KLF11 | No | 0,4 |
| 1,573588583 | GBP4 | Yes | 0,5 |
| 1,573588583 | GBP4 | No | 0,5 |
| -0,659547152 | PPP1R16B | Yes | 0,714285714 |
| -0,659547152 | PPP1R16B | No | 0,285714286 |
| -0,671629633 | ZAP70 | No | 0,363636364 |
| -0,671629633 | ZAP70 | Yes | 0,636363636 |
| 1,324342053 | UAP1L1 | Yes | 1 |
| -0,791033941 | TSPAN14 | Yes | 0,6875 |
| -0,791033941 | TSPAN14 | No | 0,3125 |
| -1,982021254 | KSR2 | Yes | 1 |
| 1,666113598 | UPP1 | Yes | 1 |
| -5,321120687 | ZNF703 | Yes | 0,785714286 |
| -5,321120687 | ZNF703 | No | 0,214285714 |
| 0,786091998 | STK38 | Yes | 1 |
| -1,290277223 | ASAP1 | Yes | 1 |
| 0,989637596 | SYTL3 | Yes | 0,571428571 |
| 0,989637596 | SYTL3 | No | 0,428571429 |
| 0,947371792 | TPR | Yes | 1 |
| 2,379286769 | GPA33 | No | 0,333333333 |
| 2,379286769 | GPA33 | Yes | 0,666666667 |
| -0,81559017 | DR1 | Yes | 1 |
| 0,865889919 | MPHOSPH8 | No | 0,5 |
| 0,865889919 | MPHOSPH8 | Yes | 0,5 |
| -2,461190178 | CHGB | No | 0,625 |
| -2,461190178 | CHGB | Yes | 0,375 |
| 1,536586291 | CSF1 | Yes | 1 |
| -1,007231222 | CD4 | Yes | 0,5 |
| -1,007231222 | CD4 | No | 0,5 |
| 0,698104692 | TRIM22 | Yes | 1 |
| -4,575446296 | INSM1 | Yes | 1 |
| 0,753107962 | RASA3 | No | 0,538461538 |
| 0,753107962 | RASA3 | Yes | 0,461538462 |
| 0,76873121 | SAMD9 | Yes | 0,75 |
| 0,76873121 | SAMD9 | No | 0,25 |
| 2,07507222 | TNFRSF13B | No | 0,5 |
| 2,07507222 | TNFRSF13B | Yes | 0,5 |
| 1,022924753 | SMCHD1 | Yes | 0,5 |
| 1,022924753 | SMCHD1 | No | 0,5 |
| 0,73839412 | SNX9 | No | 0,277777778 |
| 0,73839412 | SNX9 | Yes | 0,722222222 |
| 0,825399787 | MTPAP | Yes | 1 |
| -1,441615757 | RASSF2 | Yes | 1 |
| 1,63692371 | GPR155 | Yes | 0,875 |
| 1,63692371 | GPR155 | No | 0,125 |
| 0,70800897 | INPP5D | Yes | 0,857142857 |
| 0,70800897 | INPP5D | No | 0,142857143 |
| -5,345285532 | LINC00593 | Yes | 0,875 |
| -5,345285532 | LINC00593 | No | 0,125 |
| 1,34887301 | MUC20 | No | 0,75 |
| 1,34887301 | MUC20 | Yes | 0,25 |
| 0,768600234 | SNHG1 | Yes | 1 |
| -4,117034843 | HEYL | No | 0,25 |
| -4,117034843 | HEYL | Yes | 0,75 |
| -1,003136047 | RILPL2 | Yes | 0,666666667 |
| -1,003136047 | RILPL2 | No | 0,333333333 |
| 0,985276153 | LEF1 | Yes | 0,666666667 |
| 0,985276153 | LEF1 | No | 0,333333333 |
| -0,941209529 | DNAAF2 | Yes | 0,666666667 |
| -0,941209529 | DNAAF2 | No | 0,333333333 |
| -0,851830942 | USP36 | Yes | 0,8 |
| -0,851830942 | USP36 | No | 0,2 |
| -0,791235153 | ATP6V0E1 | Yes | 0,5 |
| -0,791235153 | ATP6V0E1 | No | 0,5 |
| 2,062042707 | CCR8 | Yes | 1 |
| 0,727033314 | RPL37 | Yes | 0,5 |
| 0,727033314 | RPL37 | No | 0,5 |
| -5,184045619 | LDHD | Yes | 0,833333333 |
| -5,184045619 | LDHD | No | 0,166666667 |
| -4,587229715 | PRR5L | Yes | 0,923076923 |
| -4,587229715 | PRR5L | No | 0,076923077 |
| -5,243406445 | GPR153 | Yes | 0,666666667 |
| -5,243406445 | GPR153 | No | 0,333333333 |
| -0,765619738 | TKT | Yes | 0,5 |
| -0,765619738 | TKT | No | 0,5 |
| -1,186086169 | CD200 | No | 0,222222222 |
| -1,186086169 | CD200 | Yes | 0,777777778 |
| -5,150397201 | GAL3ST2 | Yes | 1 |
| -0,907986052 | PPP2CA | No | 0,5 |
| -0,907986052 | PPP2CA | Yes | 0,5 |
| 2,295149034 | GOLGA7B | No | 0,4 |
| 2,295149034 | GOLGA7B | Yes | 0,6 |
| -0,82304737 | SPCS2 | Yes | 1 |
| 0,769752674 | EPB41 | Yes | 0,444444444 |
| 0,769752674 | EPB41 | No | 0,555555556 |
| -5,343024545 | HAL | Yes | 1 |
| 0,66727221 | GAS5 | Yes | 1 |
| 0,855867085 | ERCC5 | Yes | 0,666666667 |
| 0,855867085 | ERCC5 | No | 0,333333333 |
| 0,772754755 | TPT1 | Yes | 1 |
| -1,762334151 | BLK | Yes | 0,8 |
| -1,762334151 | BLK | No | 0,2 |
| 0,913586302 | PELI1 | Yes | 0,714285714 |
| 0,913586302 | PELI1 | No | 0,285714286 |
| 0,962116843 | ZNF33A | Yes | 0,666666667 |
| 0,962116843 | ZNF33A | No | 0,333333333 |
| -1,263558868 | UBE2E3 | Yes | 0,642857143 |
| -1,263558868 | UBE2E3 | No | 0,357142857 |
| 0,976950925 | MALAT1 | Yes | 0,8 |
| 0,976950925 | MALAT1 | No | 0,2 |
| 0,848741372 | FAS | Yes | 1 |
| 1,311387762 | TRABD2A | Yes | 0,833333333 |
| 1,311387762 | TRABD2A | No | 0,166666667 |
| 0,624701092 | RPS15A | No | 0,5 |
| 0,624701092 | RPS15A | Yes | 0,5 |
| -1,334125405 | SLC36A4 | No | 0,5 |
| -1,334125405 | SLC36A4 | Yes | 0,5 |
| -0,627585095 | PPP2R5C | No | 0,176470588 |
| -0,627585095 | PPP2R5C | Yes | 0,823529412 |
| 1,229916758 | NEDD4 | No | 0,25 |
| 1,229916758 | NEDD4 | Yes | 0,75 |
| 1,066076349 | RNPC3 | No | 0,666666667 |
| 1,066076349 | RNPC3 | Yes | 0,333333333 |
| 1,10183357 | IL18R1 | Yes | 0,666666667 |
| 1,10183357 | IL18R1 | No | 0,333333333 |
| -3,967653536 | SNORA66 | No | 0,75 |
| -3,967653536 | SNORA66 | Yes | 0,25 |
| -1,112856278 | CORO1B | Yes | 0,333333333 |
| -1,112856278 | CORO1B | No | 0,666666667 |
| -1,052814916 | ZBTB7B | No | 0,833333333 |
| -1,052814916 | ZBTB7B | Yes | 0,166666667 |
| -4,366034764 | HEY1 | Yes | 1 |
| -0,688944075 | ACTB | No | 0,571428571 |
| -0,688944075 | ACTB | Yes | 0,428571429 |
| 0,927168952 | DOCK10 | No | 0,428571429 |
| 0,927168952 | DOCK10 | Yes | 0,571428571 |
| -5,219568957 | NKG7 | Yes | 1 |
| -3,408250906 | TMEM63C | Yes | 1 |
| -0,891095448 | CTSB | Yes | 0,833333333 |
| -0,891095448 | CTSB | No | 0,166666667 |
| 0,917773432 | ENTPD6 | Yes | 1 |
| -0,819333086 | MARK2 | No | 0,2 |
| -0,819333086 | MARK2 | Yes | 0,8 |
| -4,579272795 | EMID1 | Yes | 1 |
| -0,830068981 | CST7 | No | 0,3 |
| -0,830068981 | CST7 | Yes | 0,7 |
| -5,041237366 | TEAD1 | Yes | 1 |
| -0,96662935 | PPP1CA | Yes | 0,5 |
| -0,96662935 | PPP1CA | No | 0,5 |
| 0,744134881 | FNDC3A | Yes | 1 |
| -0,959167341 | SAE1 | No | 0,75 |
| -0,959167341 | SAE1 | Yes | 0,25 |
| -0,990823937 | UBE2S | No | 0,5 |
| -0,990823937 | UBE2S | Yes | 0,5 |
| 2,495398484 | LRRC32 | Yes | 1 |
| 1,178861251 | BMI1 | Yes | 1 |
| 0,872937451 | APOL6 | Yes | 1 |
| -0,669029734 | JARID2 | Yes | 0,743589744 |
| -0,669029734 | JARID2 | No | 0,256410256 |
| -1,676724664 | CTTN | Yes | 1 |
| -3,940390334 | PCDHGB3 | Yes | 1 |
| 1,976434045 | FLT4 | No | 0,75 |
| 1,976434045 | FLT4 | Yes | 0,25 |
| -2,505694094 | GFOD1 | Yes | 0,9375 |
| -2,505694094 | GFOD1 | No | 0,0625 |
| -4,789215932 | SLC47A2 | Yes | 1 |
| 0,749130825 | S100A6 | Yes | 1 |
| -4,880041445 | HTRA1 | Yes | 1 |
| -4,706034406 | PDGFD | Yes | 0,5 |
| -4,706034406 | PDGFD | No | 0,5 |
| 1,073816357 | SLC30A9 | Yes | 1 |
| -0,647418464 | ZFP36L2 | Yes | 0,454545455 |
| -0,647418464 | ZFP36L2 | No | 0,545454545 |
| 0,817744734 | SP110 | Yes | 1 |
| -0,904025863 | BAK1 | Yes | 0,096774194 |
| -0,904025863 | BAK1 | No | 0,903225806 |
| -1,048585672 | CASP9 | Yes | 0,5 |
| -1,048585672 | CASP9 | No | 0,5 |
| -1,757812201 | NMB | No | 0,25 |
| -1,757812201 | NMB | Yes | 0,75 |
| 0,960274819 | KCNQ1OT1 | Yes | 1 |
| 0,637302873 | CNTRL | Yes | 1 |
| -4,850193418 | ADAMTS7 | Yes | 1 |
| -0,567367394 | RAC2 | No | 0,8 |
| -0,567367394 | RAC2 | Yes | 0,2 |
| -4,509998747 | CCL5 | No | 0,333333333 |
| -4,509998747 | CCL5 | Yes | 0,666666667 |
| 0,728713454 | ANKRD12 | No | 0,714285714 |
| 0,728713454 | ANKRD12 | Yes | 0,285714286 |
| 1,154164504 | TTC37 | Yes | 1 |
| -1,759250444 | SLC9A9 | Yes | 0,705882353 |
| -1,759250444 | SLC9A9 | No | 0,294117647 |
| 1,187439958 | INPP5F | Yes | 0,833333333 |
| 1,187439958 | INPP5F | No | 0,166666667 |
| 0,975774923 | POLK | Yes | 1 |
| 1,18488408 | CD83 | Yes | 0,375 |
| 1,18488408 | CD83 | No | 0,625 |
| 1,228275616 | CLNK | No | 0,352941176 |
| 1,228275616 | CLNK | Yes | 0,647058824 |
| -1,230280676 | FAM110A | Yes | 0,375 |
| -1,230280676 | FAM110A | No | 0,625 |
| -4,242162002 | LINC00892 | Yes | 0,818181818 |
| -4,242162002 | LINC00892 | No | 0,181818182 |
| -0,558901628 | JAK3 | No | 0,5 |
| -0,558901628 | JAK3 | Yes | 0,5 |
| 6,069961076 | CEACAM4 | Yes | 1 |
| 0,733636411 | TAB2 | Yes | 1 |
| -0,588095314 | UBTF | Yes | 0,333333333 |
| -0,588095314 | UBTF | No | 0,666666667 |
| 0,877272785 | CASP8 | No | 0,428571429 |
| 0,877272785 | CASP8 | Yes | 0,571428571 |
| -0,791660081 | ADRM1 | Yes | 0,5 |
| -0,791660081 | ADRM1 | No | 0,5 |
| 1,265525833 | SLC4A5 | Yes | 1 |
| -0,908071253 | COA1 | Yes | 0,6 |
| -0,908071253 | COA1 | No | 0,4 |
| -1,238632548 | RPIA | No | 0,5 |
| -1,238632548 | RPIA | Yes | 0,5 |
| -0,847589928 | DZIP3 | Yes | 1 |
| -0,850440276 | GOLGA7 | Yes | 0,6 |
| -0,850440276 | GOLGA7 | No | 0,4 |
| -0,766031859 | EMB | No | 0,75 |
| -0,766031859 | EMB | Yes | 0,25 |
| -1,001421568 | ASF1A | Yes | 0,9 |
| -1,001421568 | ASF1A | No | 0,1 |
| -1,140237847 | TP53INP2 | No | 0,666666667 |
| -1,140237847 | TP53INP2 | Yes | 0,333333333 |
| -4,370372961 | LONRF2 | Yes | 1 |
| -3,846011786 | PTPN13 | Yes | 1 |
| -1,146262861 | STK39 | Yes | 0,793103448 |
| -1,146262861 | STK39 | No | 0,206896552 |
| -0,772159703 | ULK1 | No | 0,5 |
| -0,772159703 | ULK1 | Yes | 0,5 |
| -1,301195881 | SLC7A5 | Yes | 0,888888889 |
| -1,301195881 | SLC7A5 | No | 0,111111111 |
| 1,013830668 | CLASP2 | No | 0,666666667 |
| 1,013830668 | CLASP2 | Yes | 0,333333333 |
| -1,398035834 | FABP5 | No | 0,333333333 |
| -1,398035834 | FABP5 | Yes | 0,666666667 |
| 0,71013387 | ZC3H7A | Yes | 0,833333333 |
| 0,71013387 | ZC3H7A | No | 0,166666667 |
| 0,544099184 | RPS27A | Yes | 0,75 |
| 0,544099184 | RPS27A | No | 0,25 |
| 1,922068019 | ARHGAP5 | Yes | 1 |
| 0,878033129 | ODF2L | Yes | 1 |
| -0,696939145 | CAPNS1 | No | 0,666666667 |
| -0,696939145 | CAPNS1 | Yes | 0,333333333 |
| -1,389729107 | GADD45G | Yes | 0,666666667 |
| -1,389729107 | GADD45G | No | 0,333333333 |
| -1,737472654 | ZNF827 | No | 0,5 |
| -1,737472654 | ZNF827 | Yes | 0,5 |
| -4,707862203 | CRMP1 | Yes | 1 |
| -2,050349323 | DNAJB5 | No | 0,75 |
| -2,050349323 | DNAJB5 | Yes | 0,25 |
| 1,699056069 | CASP1 | Yes | 1 |
| -1,739968499 | PHEX | Yes | 0,875 |
| -1,739968499 | PHEX | No | 0,125 |
| -0,638415636 | PGAM1 | No | 0,25 |
| -0,638415636 | PGAM1 | Yes | 0,75 |
| -1,00675755 | GPR18 | Yes | 0,875 |
| -1,00675755 | GPR18 | No | 0,125 |
| 0,658279353 | RANBP9 | Yes | 0,888888889 |
| 0,658279353 | RANBP9 | No | 0,111111111 |
| 0,758704848 | VPS54 | Yes | 0,625 |
| 0,758704848 | VPS54 | No | 0,375 |
| 0,923613144 | ZNF292 | Yes | 0,444444444 |
| 0,923613144 | ZNF292 | No | 0,555555556 |
| -4,581218725 | BCAT1 | Yes | 0,882352941 |
| -4,581218725 | BCAT1 | No | 0,117647059 |
| 0,742568695 | PDE4DIP | Yes | 0,666666667 |
| 0,742568695 | PDE4DIP | No | 0,333333333 |
| -0,619423173 | ALDOA | No | 0,666666667 |
| -0,619423173 | ALDOA | Yes | 0,333333333 |
| 1,32968793 | PKD2 | Yes | 1 |
| -2,529692259 | ANXA1 | Yes | 1 |
| 0,723941135 | BMPR2 | Yes | 0,5 |
| 0,723941135 | BMPR2 | No | 0,5 |
| -0,985485447 | EHD4 | No | 0,8 |
| -0,985485447 | EHD4 | Yes | 0,2 |
| -4,568313672 | CBFA2T3 | Yes | 0,8 |
| -4,568313672 | CBFA2T3 | No | 0,2 |
| -1,552688298 | DUSP5 | No | 0,5 |
| -1,552688298 | DUSP5 | Yes | 0,5 |
| 2,743673019 | SLC14A1 | Yes | 0,875 |
| 2,743673019 | SLC14A1 | No | 0,125 |
| 0,692051961 | CEP120 | Yes | 0,8 |
| 0,692051961 | CEP120 | No | 0,2 |
| -0,764034461 | IL6R | No | 0,214285714 |
| -0,764034461 | IL6R | Yes | 0,785714286 |
| -0,779019426 | NDRG3 | Yes | 0,5 |
| -0,779019426 | NDRG3 | No | 0,5 |
| 0,97299023 | CLUHP3 | Yes | 1 |
| -0,770705577 | MBP | No | 0,192307692 |
| -0,770705577 | MBP | Yes | 0,807692308 |
| 1,083576367 | GPRASP1 | Yes | 1 |
| -1,734963873 | CPT1A | Yes | 1 |
| -0,610994451 | HMCES | Yes | 0,75 |
| -0,610994451 | HMCES | No | 0,25 |
| -3,505978031 | PTPN14 | Yes | 1 |
| -0,743094439 | MGAT4B | Yes | 0,333333333 |
| -0,743094439 | MGAT4B | No | 0,666666667 |
| -3,015994241 | ITPRIPL2 | Yes | 1 |
| 0,673842539 | ZNF275 | Yes | 0,5 |
| 0,673842539 | ZNF275 | No | 0,5 |
| -1,229553358 | CNN2 | No | 0,5 |
| -1,229553358 | CNN2 | Yes | 0,5 |
| 1,673062745 | DHX58 | Yes | 1 |
| -1,812588087 | MEX3B | Yes | 1 |
| 1,347474633 | SLC39A10 | No | 0,166666667 |
| 1,347474633 | SLC39A10 | Yes | 0,833333333 |
| 0,842944599 | ZC3H12D | No | 0,307692308 |
| 0,842944599 | ZC3H12D | Yes | 0,692307692 |
| 1,133610998 | ITGB3BP | Yes | 1 |
| -5,918912013 | MYO7A | Yes | 1 |
| 1,024254154 | RYK | Yes | 0,428571429 |
| 1,024254154 | RYK | No | 0,571428571 |
| 1,036496078 | FOXN3 | Yes | 0,909090909 |
| 1,036496078 | FOXN3 | No | 0,090909091 |
| -4,80339077 | ANKRD34B | Yes | 1 |
| -1,195103997 | LAT | No | 0,222222222 |
| -1,195103997 | LAT | Yes | 0,777777778 |
| -0,729716697 | AGFG1 | Yes | 1 |
| -0,737931446 | LASP1 | No | 0,5 |
| -0,737931446 | LASP1 | Yes | 0,5 |
| -0,572849674 | CIB1 | Yes | 0,8 |
| -0,572849674 | CIB1 | No | 0,2 |
| -0,672259462 | CDIPT | Yes | 1 |
| 0,573779523 | CTLA4 | Yes | 0,727272727 |
| 0,573779523 | CTLA4 | No | 0,272727273 |
| -3,930159491 | ZG16B | Yes | 1 |
| -1,509221984 | BHLHE40 | No | 0,111111111 |
| -1,509221984 | BHLHE40 | Yes | 0,888888889 |
| -4,245799139 | GJD3 | No | 0,666666667 |
| -4,245799139 | GJD3 | Yes | 0,333333333 |
| -0,710768647 | RELB | No | 0,5 |
| -0,710768647 | RELB | Yes | 0,5 |
| 1,407644227 | PDK1 | Yes | 1 |
| 0,646834873 | XIST | Yes | 1 |
| -4,416309251 | PIF1 | Yes | 0,5 |
| -4,416309251 | PIF1 | No | 0,5 |
| 0,996142407 | USP15 | Yes | 1 |
| 0,560655539 | TRAF3IP3 | No | 0,333333333 |
| 0,560655539 | TRAF3IP3 | Yes | 0,666666667 |
| -0,658634765 | ADSL | Yes | 0,875 |
| -0,658634765 | ADSL | No | 0,125 |
| 0,810572964 | EPG5 | No | 0,333333333 |
| 0,810572964 | EPG5 | Yes | 0,666666667 |
| -6,20067193 | EOMES | No | 0,571428571 |
| -6,20067193 | EOMES | Yes | 0,428571429 |
| -1,044601523 | SSBP4 | No | 0,666666667 |
| -1,044601523 | SSBP4 | Yes | 0,333333333 |
| -0,650951422 | DENND2D | No | 0,75 |
| -0,650951422 | DENND2D | Yes | 0,25 |
| -1,445489034 | RAP2B | No | 0,615384615 |
| -1,445489034 | RAP2B | Yes | 0,384615385 |
| -0,692827234 | FZR1 | No | 0,5 |
| -0,692827234 | FZR1 | Yes | 0,5 |
| -1,336147241 | APBA2 | No | 0,083333333 |
| -1,336147241 | APBA2 | Yes | 0,916666667 |
| -0,625945119 | TPD52L2 | No | 0,5 |
| -0,625945119 | TPD52L2 | Yes | 0,5 |
| -4,270464732 | KCNQ3 | Yes | 1 |
| -0,680571036 | CMIP | No | 0,333333333 |
| -0,680571036 | CMIP | Yes | 0,666666667 |
| 1,252233365 | SAMD9L | Yes | 1 |
| 3,700609323 | IL1R2 | Yes | 1 |
| -5,807378147 | CLEC2L | Yes | 1 |
| -4,541914681 | C11orf96 | Yes | 1 |
| 1,779589467 | PRDM1 | Yes | 0,740740741 |
| 1,779589467 | PRDM1 | No | 0,259259259 |
| -0,660162434 | PSMA7 | Yes | 0,5 |
| -0,660162434 | PSMA7 | No | 0,5 |
| 1,247703114 | PPP3CA | No | 0,466666667 |
| 1,247703114 | PPP3CA | Yes | 0,533333333 |
| -0,700583573 | REPIN1 | Yes | 1 |
| 0,676400812 | NSMF | No | 0,6 |
| 0,676400812 | NSMF | Yes | 0,4 |
| 0,52497056 | SP100 | Yes | 0,666666667 |
| 0,52497056 | SP100 | No | 0,333333333 |
| 0,743207641 | CDC14A | Yes | 0,722222222 |
| 0,743207641 | CDC14A | No | 0,277777778 |
| 0,517258835 | RPS21 | Yes | 1 |
| -0,94699946 | BSG | Yes | 0,666666667 |
| -0,94699946 | BSG | No | 0,333333333 |
| -0,901901695 | SPPL2B | No | 0,666666667 |
| -0,901901695 | SPPL2B | Yes | 0,333333333 |
| 3,231469289 | DPP4 | Yes | 0,941176471 |
| 3,231469289 | DPP4 | No | 0,058823529 |
| 0,522456836 | ABCC1 | Yes | 0,857142857 |
| 0,522456836 | ABCC1 | No | 0,142857143 |
| -4,357756663 | TMEM178B | Yes | 1 |
| -0,656968181 | HIF1A | Yes | 0,75 |
| -0,656968181 | HIF1A | No | 0,25 |
| -0,780547209 | SH3GLB1 | No | 0,2 |
| -0,780547209 | SH3GLB1 | Yes | 0,8 |
| -2,123821194 | SIPA1L2 | Yes | 0,894736842 |
| -2,123821194 | SIPA1L2 | No | 0,105263158 |
| -2,130885321 | STOM | Yes | 0,666666667 |
| -2,130885321 | STOM | No | 0,333333333 |
| -1,099165139 | ASB1 | No | 0,5 |
| -1,099165139 | ASB1 | Yes | 0,5 |
| -4,066741618 | FAM78B | Yes | 1 |
| 0,516773607 | MACF1 | No | 0,307692308 |
| 0,516773607 | MACF1 | Yes | 0,692307692 |
| 1,312828898 | MFGE8 | Yes | 0,666666667 |
| 1,312828898 | MFGE8 | No | 0,333333333 |
| -1,292284679 | GADD45A | No | 0,714285714 |
| -1,292284679 | GADD45A | Yes | 0,285714286 |
| -0,997584088 | SLC10A3 | Yes | 1 |
| -3,775239202 | GDF7 | No | 0,5 |
| -3,775239202 | GDF7 | Yes | 0,5 |
| -1,271718954 | SH3TC1 | Yes | 0,833333333 |
| -1,271718954 | SH3TC1 | No | 0,166666667 |
| -0,726898336 | ARL6IP1 | No | 0,5 |
| -0,726898336 | ARL6IP1 | Yes | 0,5 |
| 2,478543852 | PCSK5 | Yes | 1 |
| 0,943980926 | KIAA1328 | Yes | 1 |
| -0,685076899 | NR4A2 | Yes | 0,272727273 |
| -0,685076899 | NR4A2 | No | 0,727272727 |
| 1,16484746 | FYCO1 | Yes | 1 |
| -1,319709851 | ZMAT3 | Yes | 0,666666667 |
| -1,319709851 | ZMAT3 | No | 0,333333333 |
| -0,657737848 | RBM42 | Yes | 0,5 |
| -0,657737848 | RBM42 | No | 0,5 |
| 0,844705572 | HABP4 | No | 0,5 |
| 0,844705572 | HABP4 | Yes | 0,5 |
| -0,497330638 | IKZF1 | Yes | 0,592592593 |
| -0,497330638 | IKZF1 | No | 0,407407407 |
| -0,802391099 | GTPBP1 | No | 0,666666667 |
| -0,802391099 | GTPBP1 | Yes | 0,333333333 |
| -0,620398252 | SREBF2 | No | 0,333333333 |
| -0,620398252 | SREBF2 | Yes | 0,666666667 |
| 0,570687206 | IRF1 | No | 0,235294118 |
| 0,570687206 | IRF1 | Yes | 0,764705882 |
| 0,622728335 | STIM2 | No | 0,181818182 |
| 0,622728335 | STIM2 | Yes | 0,818181818 |
| -4,18618636 | WDR86-AS1 | Yes | 1 |
| 1,704515381 | SUSD4 | Yes | 1 |
| -0,923089111 | REEP4 | No | 0,333333333 |
| -0,923089111 | REEP4 | Yes | 0,666666667 |
| -0,625869567 | CHST2 | No | 0,8 |
| -0,625869567 | CHST2 | Yes | 0,2 |
| -0,635045252 | TMEM50A | Yes | 1 |
| 0,782551027 | EZH1 | Yes | 1 |
| -0,860802507 | QSOX2 | Yes | 0,6 |
| -0,860802507 | QSOX2 | No | 0,4 |
| -0,949508144 | AKR1B1 | Yes | 1 |
| -0,786437411 | LRRC8D | No | 0,25 |
| -0,786437411 | LRRC8D | Yes | 0,75 |
| -0,584802841 | WDR82 | No | 0,666666667 |
| -0,584802841 | WDR82 | Yes | 0,333333333 |
| -4,217638622 | ZBTB47 | No | 0,666666667 |
| -4,217638622 | ZBTB47 | Yes | 0,333333333 |
| 0,841871906 | AAK1 | Yes | 0,875 |
| 0,841871906 | AAK1 | No | 0,125 |
| -0,680728789 | VDAC1 | No | 0,285714286 |
| -0,680728789 | VDAC1 | Yes | 0,714285714 |
| -0,649363282 | PITPNC1 | No | 0,333333333 |
| -0,649363282 | PITPNC1 | Yes | 0,666666667 |
| 3,90526979 | RNF175 | Yes | 1 |
| 0,860155973 | SNED1 | No | 0,285714286 |
| 0,860155973 | SNED1 | Yes | 0,714285714 |
| 1,982883342 | DHRS3 | No | 0,5 |
| 1,982883342 | DHRS3 | Yes | 0,5 |
| -0,739594609 | GIMAP4 | No | 0,5 |
| -0,739594609 | GIMAP4 | Yes | 0,5 |
| 1,428883889 | CCDC141 | Yes | 0,666666667 |
| 1,428883889 | CCDC141 | No | 0,333333333 |
| -5,0441965 | ATP9A | Yes | 1 |
| -0,741632357 | PPP5C | No | 0,5 |
| -0,741632357 | PPP5C | Yes | 0,5 |
| -0,754929084 | CD5 | Yes | 0,5625 |
| -0,754929084 | CD5 | No | 0,4375 |
| 0,503873788 | NFKBIA | No | 0,363636364 |
| 0,503873788 | NFKBIA | Yes | 0,636363636 |
| -0,801444325 | SH3BGRL3 | Yes | 0,4 |
| -0,801444325 | SH3BGRL3 | No | 0,6 |
| 0,589934414 | S100A4 | Yes | 1 |
| 1,094780743 | RRM1 | No | 0,5 |
| 1,094780743 | RRM1 | Yes | 0,5 |
| -3,778331556 | MME | No | 0,666666667 |
| -3,778331556 | MME | Yes | 0,333333333 |
| -0,708203532 | CD6 | Yes | 0,882352941 |
| -0,708203532 | CD6 | No | 0,117647059 |
| 0,701112735 | ZNF280D | Yes | 0,4 |
| 0,701112735 | ZNF280D | No | 0,6 |
| -0,507957327 | RAN | No | 0,666666667 |
| -0,507957327 | RAN | Yes | 0,333333333 |
| -0,581740736 | SDCBP | No | 0,666666667 |
| -0,581740736 | SDCBP | Yes | 0,333333333 |
| -0,615887282 | SGPP2 | Yes | 1 |
| -0,718578154 | SERP1 | Yes | 0,75 |
| -0,718578154 | SERP1 | No | 0,25 |
| -4,138235493 | LGR6 | No | 0,666666667 |
| -4,138235493 | LGR6 | Yes | 0,333333333 |
| -0,645643036 | LTBP3 | Yes | 0,6 |
| -0,645643036 | LTBP3 | No | 0,4 |
| -1,169270946 | GRAMD4 | No | 0,2 |
| -1,169270946 | GRAMD4 | Yes | 0,8 |
| 0,573980734 | FNBP4 | Yes | 0,666666667 |
| 0,573980734 | FNBP4 | No | 0,333333333 |
| -4,652734176 | MERTK | Yes | 1 |
| 0,552263772 | MALT1 | Yes | 0,583333333 |
| 0,552263772 | MALT1 | No | 0,416666667 |
| -0,727928038 | AURKAIP1 | Yes | 0,5 |
| -0,727928038 | AURKAIP1 | No | 0,5 |
| -3,991551515 | MS4A6A | Yes | 1 |
| -0,807361357 | LIME1 | Yes | 0,666666667 |
| -0,807361357 | LIME1 | No | 0,333333333 |
| 1,133923159 | LZTS3 | Yes | 1 |
| -4,484980932 | NFIA | Yes | 0,933333333 |
| -4,484980932 | NFIA | No | 0,066666667 |
| 0,706366836 | RPS3A | Yes | 1 |
| -0,5087249 | UBE2D3 | No | 0,625 |
| -0,5087249 | UBE2D3 | Yes | 0,375 |
| -3,975977205 | KCNG1 | Yes | 1 |
| -0,569429008 | ZNF512B | Yes | 1 |
| 1,508518418 | RPS6KA2 | Yes | 0,5 |
| 1,508518418 | RPS6KA2 | No | 0,5 |
| -4,057572406 | MYO1E | Yes | 0,8 |
| -4,057572406 | MYO1E | No | 0,2 |
| -3,609792964 | CLEC11A | Yes | 1 |
| 1,449834773 | ANKRD36BP2 | Yes | 0,75 |
| 1,449834773 | ANKRD36BP2 | No | 0,25 |
| 1,324631382 | ENTPD1 | Yes | 1 |
| -0,480682153 | YPEL5 | No | 0,166666667 |
| -0,480682153 | YPEL5 | Yes | 0,833333333 |
| -4,020915271 | MMP17 | Yes | 0,75 |
| -4,020915271 | MMP17 | No | 0,25 |
| 0,546086379 | PHF3 | No | 0,5 |
| 0,546086379 | PHF3 | Yes | 0,5 |
| -1,036041093 | MRPL14 | Yes | 0,333333333 |
| -1,036041093 | MRPL14 | No | 0,666666667 |
| -0,670558077 | NUDT16L1 | Yes | 0,571428571 |
| -0,670558077 | NUDT16L1 | No | 0,428571429 |
| 0,51061732 | SIGIRR | Yes | 0,666666667 |
| 0,51061732 | SIGIRR | No | 0,333333333 |
| 1,452476558 | LFNG | Yes | 0,8 |
| 1,452476558 | LFNG | No | 0,2 |
| -0,904360877 | CD226 | No | 0,2 |
| -0,904360877 | CD226 | Yes | 0,8 |
| 1,812671709 | NFE2L3 | Yes | 0,6 |
| 1,812671709 | NFE2L3 | No | 0,4 |
| 1,398196561 | ADAM8 | Yes | 0,4 |
| 1,398196561 | ADAM8 | No | 0,6 |
| 3,996630081 | COL5A1 | Yes | 1 |
| -1,235169283 | SIAH2 | Yes | 0,75 |
| -1,235169283 | SIAH2 | No | 0,25 |
| -0,804123704 | HGSNAT | No | 0,25 |
| -0,804123704 | HGSNAT | Yes | 0,75 |
| -3,753643899 | HPGDS | Yes | 1 |
| -0,561392738 | KDM6B | Yes | 0,166666667 |
| -0,561392738 | KDM6B | No | 0,833333333 |
| -3,663225224 | KRTAP16-1 | Yes | 1 |
| -0,752308218 | ZNF609 | Yes | 0,888888889 |
| -0,752308218 | ZNF609 | No | 0,111111111 |
| 1,403835111 | PIBF1 | Yes | 1 |
| -0,765882466 | GNB2 | No | 0,666666667 |
| -0,765882466 | GNB2 | Yes | 0,333333333 |
| 0,760623888 | BRWD1 | Yes | 1 |
| -0,946610853 | MAPRE2 | Yes | 0,466666667 |
| -0,946610853 | MAPRE2 | No | 0,533333333 |
| -4,551618403 | TMEM200A | Yes | 0,7 |
| -4,551618403 | TMEM200A | No | 0,3 |
| -2,097822544 | NEURL1B | Yes | 1 |
| -1,35277579 | FAM43A | No | 0,666666667 |
| -1,35277579 | FAM43A | Yes | 0,333333333 |
| 1,019637241 | ZNF138 | Yes | 1 |
| 0,731090321 | MDN1 | Yes | 1 |
| 2,734060936 | ARAP3 | Yes | 0,75 |
| 2,734060936 | ARAP3 | No | 0,25 |
| 0,771285713 | TTF1 | Yes | 1 |
| 1,043587209 | ABCC5 | Yes | 1 |
| 0,553783947 | INPP4A | Yes | 0,666666667 |
| 0,553783947 | INPP4A | No | 0,333333333 |
| -1,325739038 | IER5L | No | 0,5 |
| -1,325739038 | IER5L | Yes | 0,5 |
| -0,533441704 | PFKFB3 | Yes | 0,4 |
| -0,533441704 | PFKFB3 | No | 0,6 |
| 1,657796225 | RBMS3 | Yes | 1 |
| -0,472756574 | WIPF1 | No | 0,7 |
| -0,472756574 | WIPF1 | Yes | 0,3 |
| 2,051675729 | KLF8 | No | 0,333333333 |
| 2,051675729 | KLF8 | Yes | 0,666666667 |
| -0,882383938 | GAS6 | Yes | 1 |
| 1,142139094 | PLEKHA1 | Yes | 1 |
| -0,572398767 | GUK1 | No | 0,8 |
| -0,572398767 | GUK1 | Yes | 0,2 |
| 1,660886924 | HDAC9 | No | 0,473684211 |
| 1,660886924 | HDAC9 | Yes | 0,526315789 |
| -0,613059212 | ANKRD13D | Yes | 0,333333333 |
| -0,613059212 | ANKRD13D | No | 0,666666667 |
| -1,146017937 | BCL6 | Yes | 0,434782609 |
| -1,146017937 | BCL6 | No | 0,565217391 |
| -0,899845016 | LIMS1 | No | 0,235294118 |
| -0,899845016 | LIMS1 | Yes | 0,764705882 |
| -1,052097247 | SESN2 | No | 0,666666667 |
| -1,052097247 | SESN2 | Yes | 0,333333333 |
| 0,574489438 | MED23 | No | 0,5 |
| 0,574489438 | MED23 | Yes | 0,5 |
| -0,559743583 | PLCL2 | Yes | 1 |
| 0,624815781 | ASXL2 | Yes | 0,8 |
| 0,624815781 | ASXL2 | No | 0,2 |
| 1,575790416 | CCDC122 | Yes | 0,333333333 |
| 1,575790416 | CCDC122 | No | 0,666666667 |
| -0,664483121 | PARVG | Yes | 0,5 |
| -0,664483121 | PARVG | No | 0,5 |
| -3,882642646 | GRIK2 | Yes | 0,857142857 |
| -3,882642646 | GRIK2 | No | 0,142857143 |
| -0,734971664 | USP38 | No | 0,75 |
| -0,734971664 | USP38 | Yes | 0,25 |
| 0,45587061 | RPS13 | Yes | 1 |
| -0,729484568 | CD81 | Yes | 1 |
| -0,572283592 | LRRC41 | Yes | 0,5 |
| -0,572283592 | LRRC41 | No | 0,5 |
| -3,805557287 | CA8 | Yes | 0,9375 |
| -3,805557287 | CA8 | No | 0,0625 |
| 1,070595441 | APOL3 | Yes | 1 |
| -0,498238076 | PSTPIP1 | Yes | 0,545454545 |
| -0,498238076 | PSTPIP1 | No | 0,454545455 |
| 0,481771119 | SF3B1 | Yes | 0,666666667 |
| 0,481771119 | SF3B1 | No | 0,333333333 |
| 0,9567365 | ZNF815P | Yes | 0,75 |
| 0,9567365 | ZNF815P | No | 0,25 |
| -1,428076754 | PLIN2 | Yes | 0,5 |
| -1,428076754 | PLIN2 | No | 0,5 |
| -0,62748554 | TIPARP | Yes | 0,833333333 |
| -0,62748554 | TIPARP | No | 0,166666667 |
