# Supplementary material for: The impact of HIV infection on the frequencies, function, spatial localization and heterogeneity of T follicular regulatory cells (TFRs) within human lymph nodes
Source: BMC Immunol. 2022 Jul 1;23:34. doi: 10.1186/s12865-022-00508-1 (PMC9250173; doi:10.1186/s12865-022-00508-1)
Supplement: Supplementary file 8 — Additional file8. TFRs subpopulation display a CD27hiCD62L+ phenotype. [file 12865_2022_508_MOESM8_ESM.docx]

**Additional file 8. TFRs subpopulation display a CD27^hi^CD62L^+^ phenotype**


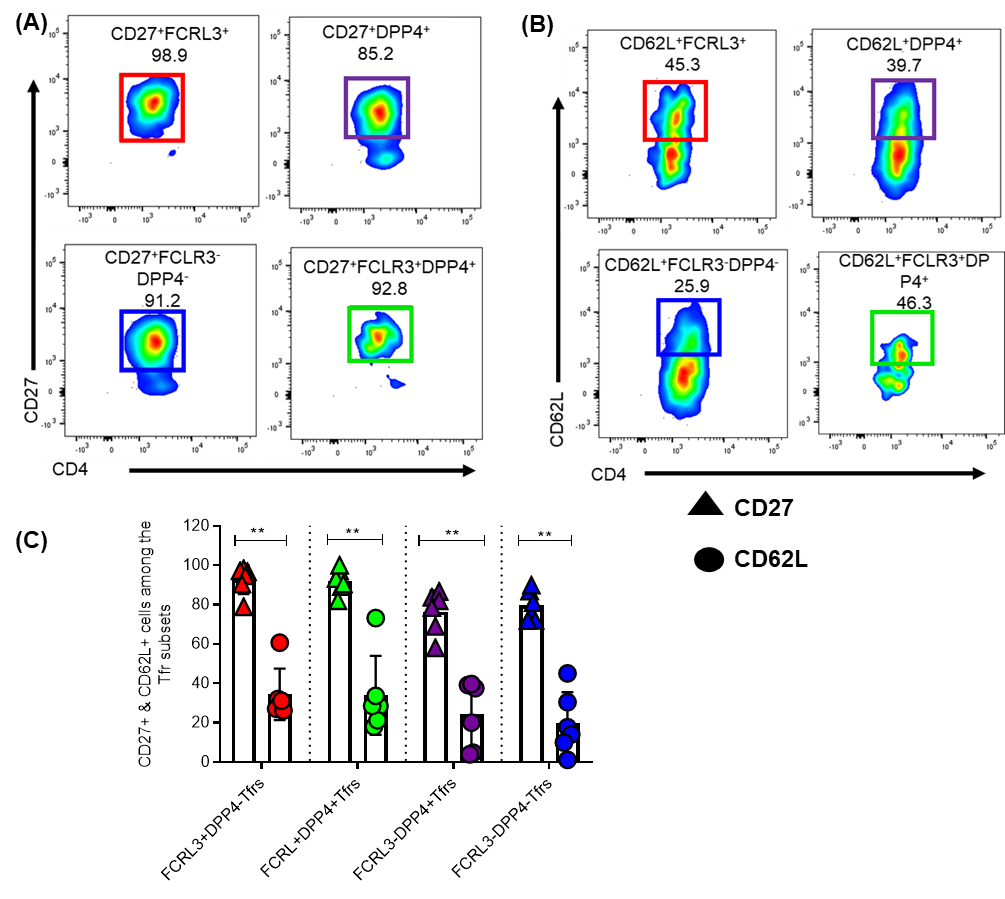


**Additional file 8. TFR subpopulations display a CD27^hi^CD62L^+^ phenotype**. Flow cytometry representative plots demonstrating **(A)** CD27^+^ and **(B)** CD62L^+^ cells and **(C)** aggregate data following gating on the four TFR subsets.
