# Supplementary material for: The impact of HIV infection on the frequencies, function, spatial localization and heterogeneity of T follicular regulatory cells (TFRs) within human lymph nodes
Source: BMC Immunol. 2022 Jul 1;23:34. doi: 10.1186/s12865-022-00508-1 (PMC9250173; doi:10.1186/s12865-022-00508-1)
Supplement: Supplementary file 9 — Additional file9. HIV-infection does not modulate DPP4 and FCRL3 expression. [file 12865_2022_508_MOESM9_ESM.docx]

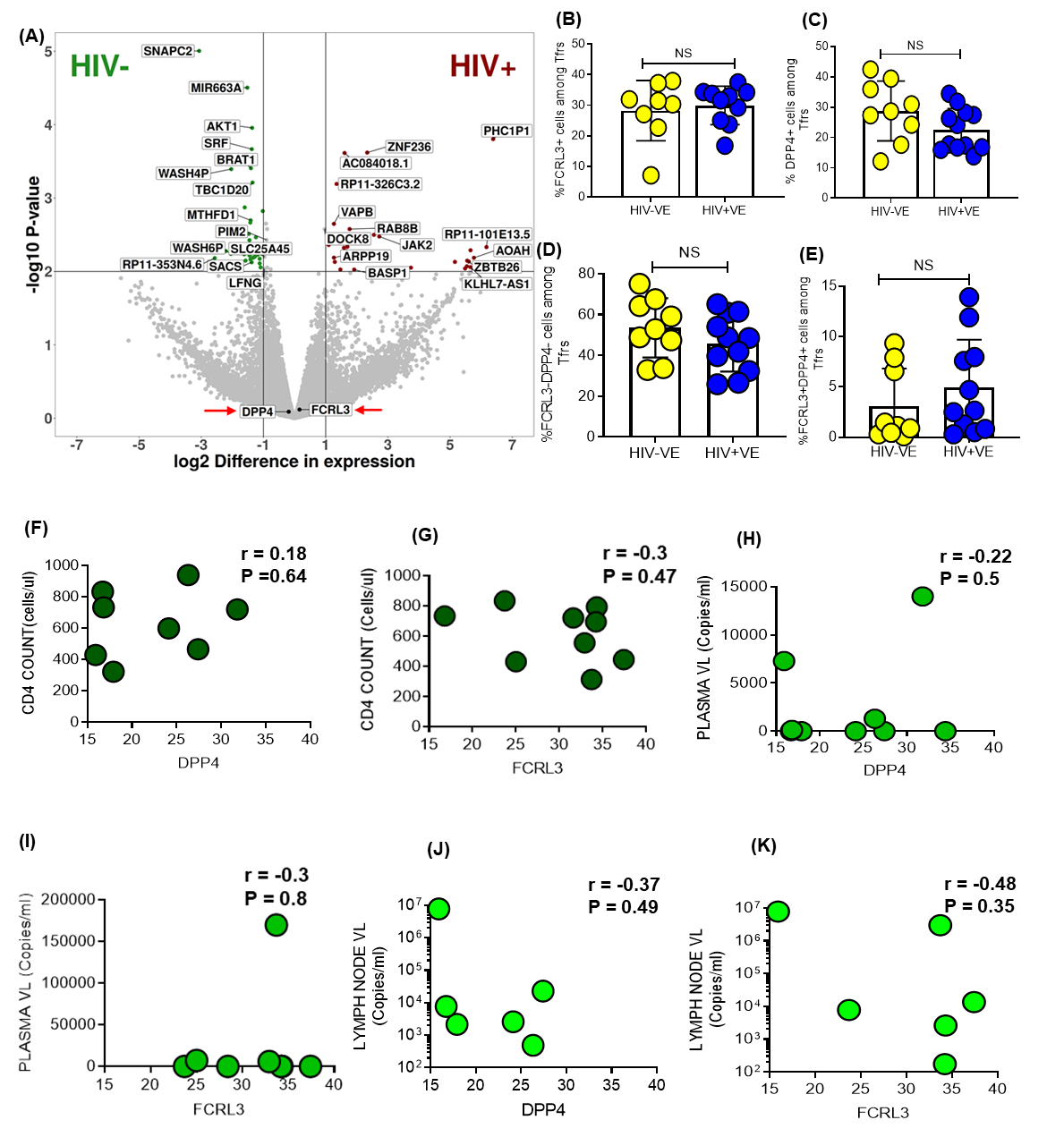


**Additional file 9. HIV-infection does not modulate DPP4 and FCRL3 expression**

**Additional file 9. HIV-infection does not modulate DPP4 and FCRL3 expression**. (**A**) Volcano plot of differentially expressed genes in TFHs from HIV-infected and HIV-uninfected donors. Summary plots demonstrating percentage of (**B**) FCLR3^+^DPP4^-^, (**C)** DPP4^+^FCLR3^-^, (**D)** DPP4^-^FCLR3^-^ and (**E)** DPP4^+^FCLR3^+^ cells among TFR in 12 HIV-infected and 9 healthy donors. (**F-K**) Correlation between absolute CD4 counts, viral loads (LN and plasma), DPP4 and FCRL3-expressing TFRs. Spearman’s rank correlation P and rho (r) values are reported.
